# FOXC1-mediated LINC00301 facilitates tumor progression and triggers an immune-suppressing microenvironment in non-small cell lung cancer by regulating the HIF1α pathway

**DOI:** 10.1186/s13073-020-00773-y

**Published:** 2020-09-02

**Authors:** Cheng-Cao Sun, Wei Zhu, Shu-Jun Li, Wei Hu, Jian Zhang, Yue Zhuo, Han Zhang, Juan Wang, Yu Zhang, Shao-Xin Huang, Qi-Qiang He, De-Jia Li

**Affiliations:** 1 Department of Preventive Medicine, School of Health Sciences, Wuhan University, No.115 Donghu Road, Wuchang District, Wuhan, 430071 Hubei People’s Republic of China; 2 Department of Molecular and Cellular Oncology, The University of Texas MD Anderson Cancer Center, Houston, TX 77030 USA; 3 Wuhan Hospital for the Prevention and Treatment of Occupational Diseases, Wuhan, 430022 Hubei People’s Republic of China; 4 School of Basic Medicine, Jiujiang University, Jiujiang, 332005 Jiangxi People’s Republic of China

**Keywords:** LINC00301, ELL protein-associated factor 2 (EAF2), Non-small cell lung cancer (NSCLC), Enhancer of zeste homolog 2 (EZH2), Regulatory T cells (Treg), Hypoxia-inducible factor 1 α (HIF1α), miR-1276, Tumorigenesis

## Abstract

**Background:**

Long non-coding RNAs (lncRNAs) are extensively intricate in the tumorigenesis and metastasis of various cancer types. Nevertheless, the detailed molecular mechanisms of lncRNA in non-small cell lung cancer (NSCLC) still remain mainly undetermined.

**Methods:**

qPCR was performed to verify LINC00301 expression in NSCLC clinical specimens or cell lines. Fluorescence in situ hybridization (FISH) was conducted to identify the localization of LINC00301 in NSCLC cells. Chromatin immunoprecipitation (ChIP) was subjected to validate the binding activity between FOXC1 and *LINC00301* promoters. RNA immunoprecipitation (RIP) was performed to explore the binding activity between LINC00301 and EZH2. RNA pull-down followed by dot-blot, protein domain mapping, and RNA electrophoresis mobility shift assay (EMSA) were conducted to identify the detailed binding regions between LINC00301 and EZH2. Alpha assay was conducted to quantitatively assess the interaction between LINC00301 and EZH2.

**Results:**

LINC00301 is highly expressed in NSCLC and closely corelated to its prognosis by analyzing the relationship between differentially expressed lncRNAs and prognosis in NSCLC samples. in vitro and in vivo experiments revealed that LINC00301 facilitates cell proliferation, releases NSCLC cell cycle arrest, promotes cell migration and invasion, and suppresses cell apoptosis in NSCLC. In addition, LINC00301 increases regulatory T cell (Treg) while decreases CD8^+^ T cell population in LA-4/SLN-205-derived tumors through targeting TGF-β. The transcription factor FOXC1 mediates LINC00301 expression in NSCLC. Bioinformatics prediction and in vitro experiments indicated that LINC00301 (83–123 nucleotide [nt]) can directly bind to the enhancer of zeste homolog 2 (EZH2) (612–727 amino acid [aa]) to promote H3K27me3 at the *ELL protein*-*associated factor 2* (*EAF2*) promoter. EAF2 directly binds and stabilizes von Hippel–Lindau protein (pVHL), so downregulated EAF2 augments hypoxia-inducible factor 1 α (HIF1α) expression by regulating pVHL in NSCLC cells. Moreover, we also found that LINC00301 could function as a competing endogenous RNA (ceRNA) against miR-1276 to expedite HIF1α expression in the cytoplasm of NSCLC cells.

**Conclusions:**

In summary, our present research revealed the oncogenic roles of LINC00301 in clinical specimens as well as cellular and animal experiments, illustrating the potential roles and mechanisms of the FOXC1/LINC00301/EZH2/EAF2/pVHL/HIF1α and FOXC1/LINC00301/miR-1276/HIF1α pathways, which provides novel insights and potential theraputic targets to NSCLC.

## Background

Non-small cell lung cancer (NSCLC) remains one of the principal triggers of deaths from cancers worldwide [1]. Therefore, illumination of the mechanisms that underlie tumorigenesis and advancement and improvement in the diagnostics and treatments of NSCLC is in urgent need. Although dysregulations on various tumor-suppressive genes and oncogenes have been elaborated in NSCLC [2–4], the detailed molecular mechanisms contributing to NSCLC pathogenesis stay to be well illuminated. Consequently, a better understanding of the mechanism of NSCLC tumorigenesis is crucial for the advancement of diagnostic markers and aids innovatively efficient therapies for NSCLC patients.

Long non-coding RNAs (lncRNAs), > 200 nucleotides (nt) in length [5], take part in pathological and physiological processes for various categories of human cancers [6–8]. Increasing evidence indicates that lncRNAs can be essential participants in cancer biology, especially resulting in the dysregulation of gene products which results in the progression of human cancers [9–12]. LncRNAs can also be deemed as prognostic or diagnostic markers based on their clinical significance in tumor outcomes [13–15].

Several lncRNAs, such as LINC00473 [16], MIR22HG [17], linc00460 [18], NEAT1 [19], LINC00511 [20], and XIST [21, 22] are related to NSCLC tumorigenesis. LINC00473, mainly located in the nucleus, is highly expressed in NSCLC, and its improved expression correlates with a worse prognosis [16]. Su et al. revealed that silencing the MIR22HG initiates cell death or survival signaling through targeting the p21, MET, and YBX1in NSCLC [17]. XIST was reported to endorse TGF-β-stimulated epithelial-mesenchymal transition (EMT) via targeting miR-367/miR-141-ZEB2 signaling in NSCLC [21]. However, the detailed molecular mechanisms of lncRNA in NSCLC remain to be deeply elucidated.

Long intergenic non-coding RNA 00301 (LINC00301, NR_026946), also called C11orf64 or NCRNA00301, is located in 11q12.2 with 7 exons and 864 bp in length. It is a freshly categorized lncRNA that is increasingly expressed by 5.29-fold in human lung cancer [23]. However, its biological functions and fundamental molecular mechanisms in NSCLC oncogenesis are completely undefined. Herein, we proposed to clarify the potential molecular mechanisms of LINC00301 in NSCLC tumorigenesis. Intriguingly, we noticed that LINC00301 is highly expressed in both tissues and cell lines of NSCLC and is mediated by TF (transcriptional factor) FOXC1. Furthermore, LINC00301 is primarily distributed in the nucleus, with several occurring in the cytoplasm. It can facilitate cell growth, inhibit cell cycle arrest and apoptosis, promote migration and invasion, and increase regulatory T cell (Treg) while decrease CD8^+^ T cell infiltration in NSCLC tumors by modulating the LINC00301/enhancer of zeste homolog 2 (EZH2)/ELL protein-associated factor 2 (EAF2)/von Hippel–Lindau (pVHL)/hypoxia-inducible factor 1 subunit alpha (HIF1α) axis in the nucleus and the LINC00301/miR-1276/HIF1α pathway in the cytoplasm.

## Methods

### Clinical tissue samples

Fresh and formalin-fixed, paraffin-embedded (FFPE), tumorous, and adjacent normal lung tissue samples were from NSCLC patients. Selective surgery was conducted on those NSCLC patients at Zhongnan Hospital of Wuhan University (Wuhan, People’s Republic of China). In sum, 120 paired fresh NSCLC tumorous and adjacent tissues (> 5 cm away from the tumor) were freshly frozen by liquid nitrogen (LN) and deposited at − 80 °C fridge. Moreover, 120 cases of archived, FFPE NSCLC tumorous, and paired adjacent normal lung tissue samples were gathered and utilized on the clinicopathological and prognostic exploration for LINC00301. None of those patients were subject to any preoperative chemo-/radiotherapy. The usage of NSCLC tumorous and adjacent normal lung tissues in this research has been supported by the ethics committee at Zhongnan Hospital of Wuhan University. All of the NSCLC patients have signed informed consent prior to utilizing the clinical resources for investigation aims.

### Survival analysis by Kaplan-Meier plotter

Kaplan-Meier plotter (http://kmplot.com/analysis/) was subjected to evaluate the impacts of 54,000 genes on survival in 21 cancer types by analyzing RNA-seq and gene chip data deposited in databases including the European Genome-phenome Archive (EGA), Gene Expression Omnibus (GEO) and The Cancer Genome Atlas (TCGA) [24]. The relationship between LINC00301, EAF2 and HIF1A expression and prognosis in 2437 lung tumors was analyzed by Kaplan-Meier plotter. Detail process for this online tool was as follows (take HIF1A as a example): input HIF1A at“Affy id/ Gene symbol” dialog box, choose “Auto select best cutoff” at “Split patients” dialog box, then select “OS (*n*=1927)”, “FP (n=982)” or “PPS (*n*=344)”, respectively at “Survival” dialog box, next, choose “all probe sets per gene” at “Probe set options” dialog box, and tick “Plot beeswarm graph of probe distribution” and “Show probe expression in normal tissue”, then tick “Dataset: each seperately” at “Use selected cohort” dialog box. Then, tick all the optional items at “Include in multivariate” dialog box, and choose “multivariate” at “Cox regression” dialog box. Lastly, click “Draw Kaplan-Meier plot”.

### Online databases used in this study

LINC00301 expression in NSCLC cell lines was assembled by Cancer Cell Line Encyclopedia (CCLE) (www.broadinstitute.org/ccle). Prediction of CpG islands in LINC00301 promoter region was conducted through the MethPrimer online software (http://www.urogene.org/cgi-bin/methprimer/methprimer.cgi) and DBCAT (http://dbcat.cgm.ntu.edu.tw/). Bioinformatics predicts the binding potential of LINC00301 to EZH2 protein. RNAfold web server (http://rna.tbi.univie.ac.at/cgi-bin/RNAWebSuite/RNAfold.cgi) was used to predict the RNA structure of LINC00301. catRAPID (http://s.tartaglialab.com/page/catrapid_group) and RPISeq (http://pridb.gdcb.iastate.edu/RPISeq/) were subjected to predict the binding potential of LINC00301 to EZH2 protein.

### DNA methylation analysis by bioinformatic tools

DNA methylation analysis of lung adenocarcinoma (LUAD) and squamous cell carcinoma (LUSC) samples was conducted using the SMART App (http://www.bioinfo-zs.com/smartapp/) [25] that is based on TCGA Pan-Cancer cohort of UCSC Xena public data hub (https://xenabrowser.net). Detail process for this online tool was as follows inputing LINC00301 at“Quick Start (Input a Gene Symbol)” dialog box, and then clicking “Go”. Next, ticking “Click to check CpG-aggregated methylation”, selecting “CpG aggregation: All”, “Aggregationn Method: Mean”, and “Methylation Value: M-value”and click “Plot”, and then click “Plot”. Next, clicking “Download data” to do download the data and isolate LUAD (normal (*n* =30); tumor (*n* =458)) and LUSC (normal (*n* =41); tumor (*n*= 364))data to be subjected to Graphpad Prism 8 (Graphpad software, San Diego, USA) for stastactis and generating images. 

### Cell culture and transfection

Ten human NSCLC cell lines (H1299, 95D, SK-MES-1, H460, H520, SK-LU-1, H1975, A549, H157, and SPC-A-1) and 2 normal lung epithelial cell lines, that is, 16HBE and BEAS-2B, were bought from the Institute of Biochemistry and Cell Biology (IBCB) of the Chinese Academy of Sciences (CAS) (Shanghai, China). Mouse NSCLC cell lines (LA-4 and KLN 205) and the normal mouse lung epithelial cell line MLE-12 were purchased from American Type Culture Collection (ATCC). H1299, 95D, SK-MES-1, H460, H520, SK-LU-1, H1975, A549, H157, and SPC-A-1 were cultured in RPMI 1640 (Gibco, Grand Island, NY, USA) medium supplemented with 10% fetal bovine serum (FBS), 100 U/ml penicillin, and 100 mg/ml streptomycin (Gibco); 16HBE cells were cultured in α-MEM medium plus 0.5 mg/mL human fibronectin, 1 mg/mL bovine serum albumin (BSA) and fraction V, and 3 mg/mL PureCol; BEAS-2B cells were cultured in BEBM medium supplemented with 0.01 mg/mL fibronectin, 0.03 mg/mL bovine collagen type I, and 0.01 mg/mL BSA; LA-4 cells were cultured in Ham’s F12K medium with 2 mM L-glutamine adjusted to contain 1.5 g/L sodium bicarbonate (SB), 15% FBS; KLN 205 cells were cultured in EMEM medium supplemented with 10% FBS; MLE-12 cells were cultured in HITES medium supplemented with 2% FBS in humidified air at 37 °C with 5% CO_2_. pLenti-CMV-LINC00301, pLenti-CMV-EAF2, pLenti-CMV-HIF1A, pLenti-CMV-LUC-LINC00301, sh-LIN00301#1, sh-LIN00301#2, and sh-LIN00301#3 were constructed by ourselves (primers are listed in Additional file 1: Table S1). DNMT1, DNMT3A, DNMT3B, HADC1, EZH2, EAF2, VHL, and FOXC1 siRNAs were purchased from Dharmacon (Dharmacon, Horizon Discovery Group Co.). Complete medium without antibiotics was applied to culturing the HEK-293FT cells at least 24 h before transfection. Cells were washed with cold 1× phosphate-buffered saline (PBS) (pH 7.4) and then transfected with pLenti-CMV-LINC00301, pLenti-CMV-EAF2, pLenti-CMV-HIF1A, pLenti-CMV-LUC-LINC00301, sh-LIN00301#1, sh-LIN00301#2, and sh-LIN00301#3, coupled with VSVG and psPAX2 lentivirus package plasmids using polyethylenimine (PEI) (764965, MilliporeSigma) cellular transfection reagent, after transfection for 24 hrs, replaced with complete medium and culture for 48 hrs, and then the supernatant containing lentivirus with related vector was collected. A549 and SPC-A-1 cells were infected with the concentrated supernatant containing lentivirus with 10 μg/mL polybrene (TR1003G, MilliporeSigma) for 24 hrs, and then 2 μg/ml puromycin (AAJ67236XF, Alfa Aesar) was added to select for 3–5 days.

### TGF-β1 ELISA analysis

TGF-β1 ELISA analysis was conducted followed the manuals of TGF beta-1 Mouse ELISA Kit (BMS608-4, Invitrogen). Briefly, 1 × 10^5^ cells (MLE-12, LA-4, MLN-205) were seeded in the 6-well plate for 48 h, then 20 μl × 3 supernatants per group were subjected for the standard ELISA protocol, and the absorbance of each microwell on a spectrophotometer was read using 450 nm on the EnSpire Multimode Plate Reader (PerkinElmer).

### Western blot assay

Western blot assay was performed following the protocol illustrated previously [14, 26]. In detail, cells were collected and lysed in radio immunoprecipitation assay (RIPA) buffer (50 mM Tris pH 7.4, 150 mM NaCl, 1 mM EDTA, 1% Nonidet P-40, 0.5% sodium deoxycholine, and 1 mM NaF) which was complemented with 1 mM Na_3_VO_4_, 1 mM PMSF, 1 mM proteinase inhibitor, and 1 mM phosphatase inhibitor immediately prior to utilizing. The following primary antibodies were applied: rabbit anti-EZH2 (5246S, 1: 1000 dilution, Cell Signaling Technology (CST), USA), rabbit anti-EAF2 (14159S, 1: 1000 dilution, CST, USA), rabbit anti-VHL (68547S, 1: 1,000 dilution, CST, USA), rabbit anti-HIF-1α (36169S, 1: 1,000 dilution, CST, USA), and rabbit anti-β-actin (4970 L, 1: 1,000 dilution, CST, USA).

### RNA isolation and quantitative real-time polymerase chain reaction (qRT-PCR)

RNA isolation and qRT-PCR were conducted utilizing the method described previously [19, 27]. β-actin and U1 were applied as the endogenous controls. The qualified expression was assessed employing the 2^−ΔΔCt^ formula. Statistical analysis was conducted using the fold change. The primer sequences utilized in this research are displayed in Additional file 1: Table S1.

### Colony formation assay

Colony formation assay was performed following the method described previously [19]. In detail, 5,000 (A549 and SPC-A-1) or 20,000 (A549, 95D, H1299, SPC-A-1) cells per well were seeded and cultured in 24- or 6-well plates for 14 days. Then, cells were washed with 1 × PBS, fixed with 4% formaldehyde, stained with 0.1% crystal violet, and recorded by EOS 90D (Canon, Japan).

### Methylation and deacetylation analysis

5-AZA (5-azacytidine) was bought from Sigma (A2385, Sigma) and dissolved in DMSO at 200 mM and stored at − 20 °C. The stock solutions were further diluted in 1 × PBS (1–20 μM; < 0.1% DMSO in the final concentration) for cell culture experiments. TSA (trichostatin A) was bought from Sigma (T8552, Sigma) and dissolved in DMSO at 100 mM and stored at − 20 °C. The stock solutions were further diluted in 1 × PBS (1–5 μM; < 0.1% DMSO in the final concentration) for cell culture experiments. To test the effects of methylation and deacetylation on LINC00301 expression, NSCLC cells were incubated in 6-well plates in the presence of 5-AZA (5 μM) or TSA (300 mM) or DMSO for 48 hrs, followed by cold 1 × PBS washes for twice, and subjected to RNA isolation, purification, and qRT-PCR analysis.

### BALB/c nude mice xenograft model

Four- to six-week-old and 16–20 g male BALB/c athymic nude mice were bought from Hubei Research Center of Laboratory Animal (Wuhan, China). All of the animal researches were conducted complying with Guidance for the Care and Use of Laboratory Animals of Wuhan University. To construct a lung cancer xenograft model, 2 × 10^6^ sh-NC or sh-1, pLenti-CMV-vector, or pLenti-CMV-LINC00301 A549/SPC-A-1, cells were suspended in 100 μL 1× PBS (pH 7.4) and subcutaneously (SubQ) injected into the flanks of 24 nude mice (*n* = 6 each). The tumor size was examined by calculating the length (*L*) and width (*W*) with calipers every 4 days, and the tumor volume was analyzed utilizing the method: (*L* × *W*^2^)/2. Mice were to proceed with general anesthesia and then killed by cervical dislocation in day 36, and the tumors were isolated and applied to FFPE or snap-frozen for protein and RNA analysis.

### RNA immunoprecipitation (RIP) assay

RIP assay was performed as described previously [28]. In detail, 1 × 10^7^ A549 and SPC-A-1 cells were washed with cold 1 × PBS for twice and then collected and lysed in 1 mL ice-cold polysomal lysis buffer (10 mM HEPES pH 7.0, 5 mM MgCl_2_, 1 mM dithiothreitol (DTT), 100 mM KCl, 0.5% NP-40, supplemented with protease inhibitor cocktail (B14002, Bimake) and protector RNase inhibitor (3335399001, Roche). Turbo DNase (200 U) (AM2238, Invitrogen) was then referred to the lysate and incubated on 4 °C with rotation for 30 min. Then, cell lysate was diluted in the NT2 buffer (50 mM Tris-HCl pH 7.4, 1 mM MgCl_2_, 150 mM NaCl, 0.05% NP-40) and 50 μL of the diluted mixtures were spared as the input for further PCR analysis. Next, 50 μL protein G magnetic beads (161-4023, Bio-Rad) were washed twice by cold NT2 buffer, and pre-blocked by 1× PBS plus 5 mg/mL BSA, after washing for twice by NT2 buffer, and the pre-blocked beads were incubated with 5 μg of rabbit IgG (2729S, CST), EZH2 (5246S, CST), SUZ12 (3737S, CST), WDR5(13105S, CST), or LSD1 (2184S, CST) antibodies with rotation at room temperature (RT) for 1 hrs. Subsequently, 500 μL supernatant was mixed with antibody-binding beads with rotation at 4 °C overnight. The RNA/antibody/protein complex was washed six times (1 mL NT2 buffer with 5 min for each wash) supplemented with protease inhibitor cocktail and protector RNase inhibitor. The RNA was extricated using acid phenol to chloroform, pH 4.5 (with IAA, 125:24:1) (AM9722, Invitrogen), following the manufacturer’s procedures and exposed to qPCR analysis.

### RNA pull-down assays

LINC00301 was constructed into the pGEM-3Z vector (P2151, Promega) using KpnI (R3142, New England Biolabs) and NheI (R3131, New England Biolabs) restriction enzyme cutting site. Then, pGEM-3Z-LINC00301 was cut into liner DNA using KpnI and NheI restriction enzyme separately. Sense and antisense of LINC00301 RNA were transcribed using MEGAscript™ T7 Transcription Kit (AM1334, Invitrogen, USA) and MEGAscript™ SP6 Transcription Kit (AM1330, Invitrogen, USA) in vitro, respectively. The transcribed LINC00301 was purified by RNA Clean & Concentrator-5 (R1013, Zymo Research, USA). Purified RNAs were biotin-labeled with the Pierce™ Biotin 3′ End DNA Labeling Kit (89818, Thermo Scientific, USA). Positive/negative control and biotinylated RNAs were mixed and incubated with A549 or SPC-A-1 cell lysates at NT2 buffer. Subsequently, the preclear magnetic beads were added to each binding reaction and incubated with rotation at RT. Ultimately, the magnetic beads were washed twice by NT2 buffer and digested by proteinase K (P8107, Promega), and then protein/RNA was isolated for western blot and qRT-PCR assay.

### RNA fluorescence in situ hybridization (FISH)

RNA FISH assay was conducted using locked nucleic acid (LNA) FISH technology following the manufacturer’s procedure (Exiqon) with minor adjustments. In detail, A549 or SPC-A-1 cells with related treatment were fixed using 4% formaldehyde/5% acetic acid for 15 min followed by twice washes with cold 1 × PBS. Then, the fixed cells were additionally handled by 1% pepsin (10108057001, Roche) and followed by dehydration using 70%, 90%, and 100% ethanol. Next, cells underwent air-dried treatment and were proceeded to incubation with 40 nM LNA FISH probe in hybridization buffer (10% formamide and 100 mg/mL dextran sulfate in 2 × saline-sodium citrates (SSC)) at 80 °C for 2 min. Subsequently, the hybridized mixtures were subjected to 55 °C of incubation for 2 hrs and washed for twice with 0.1 × SSC at 65 °C followed by dehydration via 70%, 90%, and 100% ethanol. Then, the slides were air-dried and mounted with ProLong™ Gold Antifade Mountant with DAPI (P10144, Invitrogen, USA) for detection. LNA FISH probes targeting LINC00301 were designed by Qiagen’s online design software.

### In vitro RNA pull-down coupled with dot blot assay

The in vitro binding activities of LINC00301 with recombinant proteins (EZH2) and the followed purification of protein-binding LINC00301 sequences were conducted as follows. Firstly, in vitro transcribed biotinylated LINC00301 was incubated with several recombinant His-EZH2 proteins in RNA-protein binding buffer [50 mM Tris-HCl pH 7.9, 10 mM β-ME, 10% Glycerol, 5 mM MgCl_2_, 100 mM KCl, and 0.1% NP-40]. Then, the reactions were subjected to ultraviolet (UV) irradiation (150 mJ/cm^2^) to crosslink the RNA-protein complexes. Followed by UV irradiation, the RNAs were in part digested by RNase I (AM2294, Ambion, USA) at 1:50 and 1: 500 dilutions for 5 min each, letting the small fragment continue attaching with related protein. RNA-protein complexes of interest were then subjected to purification by his tag magnetic beads, and the purified RNA-protein complexes were incubated with proteinase K (P8107, Promega), which eliminated protein but left the complete RNAs. The recovered RNAs were subjected to hybridization with BrightStar-Plus Positively Charged Nylon Membrane (AM10100, Ambition, USA) pre-spotted with 41-mer antisense DNA oligonucleotides tiling along LINC00301 (sequences listed at Additional file 1: Table S1) at 37 °C overnight. Then, NT2 buffer was used to wash the hybridized membranes as defined in the progression of 37 °C, 50 °C, and 65 °C. Finally, the protein-binding RNA sequences were visualized by exposure to Streptavidin-HRP signals. The antisense oligonucleotides according to the LINC00301 were spotted on nylon membrane as the following orders (left to right in each row): A1 is related to the oligonucleotide sequences nt 1-41 of LINC00301, A2 is homologous to oligonucleotide sequences nt 42-82 of LINC00301, and so on, until the end of the LINC00301 sequences.

### RNA electrophoretic mobility shift assay (EMSA)

The RNA EMSA assay was conducted with recombinant His-EZH2 protein that purified from A549 and SPC-A-1 cells with synthesized biotinylated RNA oligonucleotides corresponding to nt. 83-143 (Sigma), respectively. pLenti-CMV-EZH2-His vector was transfected into A549 and SPC-A-1 cells, and then His-EZH2 proteins were isolated from A549 and SPC-A-1 cell lysate. Then, 1 μg recombinant His-EZH2 protein was incubated with 0.035 pmol pre-heated biotinylated LINC00301 RNA probes in RNA-protein binding buffer for 1 h at RT. After incubation, 2 μL of Novex Hi-Density Tris/Borate/EDTA (TBE) Sample Buffer (LC6678, Invitrogen) was added to the reaction mixtures, which were immediately separated on a 6% retardation gel (EC6365BOX, Invitrogen) (pre-run in 0.5 × TBE running buffer at 100 V at 4 °C for 1 h) in 0.5 × TBE running buffer at 100 V at 4 °C for 40 min. Then, RNA-protein complex was transferred to BrightStar-Plus Positively Charged Nylon Membrane (AM10100, Ambition, USA) in 0.5 × TBE transfer buffer and was exposed to UV irradiation (150 mJ/cm^2^) to crosslink RNA-protein complexes into membranes. Then, the membranes were incubated with HRP-conjugated streptavidin at 1:300 dilution (N100, Invitrogen) and visualized with Pierce™ ECL Western Blotting Substrate (32,106, Ambition, USA). For cold RNA competition, 0.035 pM biotinylated RNA probes were initially mixed with 7 pM cold unlabeled RNA competitors, and then the EMSA was conducted as described above.

### Alpha assay

Alpha binding assay was conducted to ascertain KD for the LINC00301 and EZH2 interaction. Briefly, the KD was established by competitive experiments in which unlabeled LINC00301 was titrated (2-fold dilutions) from 10 μM to 0.1 nM. Subsequently, the streptavidin donor beads and Anti-6xHis AlphaLISA Acceptor beads (AL178C, PerkinElmer) were used in these assays. The 96-well plate was read on the EnSpire Multimode Plate Reader (PerkinElmer). The competition-inhibition curves were evaluated based on alpha signal readings through matching to a “log (inhibitor) vs. response-variable slope (two parameters)” model (GraphPad Prism 8 software).

### Flow cytometry

#### Cell cycle

Cells were harvested 48 h after transfection, followed by fixing in 70% ethanol, washed twice with cold 1 × PBS, and then labeled with FxCycle™ PI/RNase Staining Solution (F10797, Invitrogen) for 30 min in the dark. Subsequently, samples were detected on a FACSalibur flow cytometer (Becton-Dickinson, FL, NJ, USA), and the percentages of cells within each phase of the cell cycle were analyzed using FCS Express 7 software (De Novo Software).

#### Cell apoptosis

Cell apoptosis was determined by FITC Annexin V Apoptosis Detection Kit I (556547, Becton-Dickinson, FL, NJ, USA) according to the manufacturer’s procedure. Cells were washed twice with cold 1 × PBS and resuspended with staining buffer (420201, BioLegend). Five microliters of annexin V-FITC and 5 μL propidium iodide (PI) were co-added into a total of 100 μL cell suspension. The mixtures were incubated for 15 min at RT in the dark and then subjected to flow cytometry analysis (FACSCalibur, Becton-Dickinson, USA).

#### Regulatory T cells

Cells were washed twice with DPBS (21-031-CM, Corning) plus 2% FBS (16000044, Gibco) and pro-blocked with TruStain FcX™ (anti-mouse CD16/32) antibody (101319, BioLegend) for 10 min on ice. Then, 1 × 10^6^ cells per condition were stained with the proper antibodies diluted in cell staining buffer (420201, BioLegend, USA) for 25 min at RT. Corresponding fluorescence minus 1 staining to each condition was presented as a control. Mouse tumors (isolated from sh-1/sh-NC, pLenti-CMV-LINC00301/pLenti-CMV-vector-transfected LA-4 or KLN 205 cells) were disassociated as every single cell adopting the gentleMACS Dissociator (130-093-235, Miltenyi Biotec) using the special mouse Tumor Dissociation kit (130-096-730, Miltenyi Biotec). After lysing red blood cells by RBC Lysis Buffer (420301, BioLegend) for 10 min on ice, single-cell suspensions were subjected to blockage with TruStain FcX™ (anti-mouse CD16/32) antibody (101319, BioLegend) for 10 min on ice and followed by incubating with the related antibodies for 25 min at RT. Mouse antibodies were bought from BioLegend CD3 (100203, BioLegend), CD4(100407, BioLegend), and CD25(101915, BioLegend). Zombie Violet fixable viability dyes (423113, BioLegend) were used to differentiate live/dead cells. Flow cytometry was conducted on an LSRII (BD Biosciences), and the data were analyzed by FlowJo (TreeStar).

### CyTOF run and sample normalization

#### Sample run and normalization

LA-4 and KLN 205 cells were transfected with sh-1/sh-NC, pLenti-CMV-LINC00301/pLenti-CMV-vector, and then transplanted to C57BL/6 J mice. After the tumor size reached to 400 mm^3^, tumors were isolated from tumor-burden mice. Tumors were disposed of as described before to get single-cell suspensions, followed by staining with cisplatin (201194, Fluidigm), blocked with TruStain FcX™ (anti-mouse CD16/32) antibody (101,319, BioLegend) for 10 min on ice, and incubation of primary surface antibodies (Abs) (Additional file 1: Table S2) for 30 min at RT at dark. Subsequently, after intracellular Ag staining was done using Fix/Perm buffer and 2 hrs of staining at 4 °C, the secondary surface antibody stains were done for another 30 min. Then, cells were washed twice with washing buffer and resuspended to incubate in Intercalator (Cell-ID Intercalator-Ir, 201192A, Fluidigm). Abs are listed in Additional file 1: Table S2 and were from Fluidigm unless noted otherwise. Samples were collected on a Helios mass cytometer (Fluidigm), with samples resuspended with equilibration beads spiked into each sample to allow for signal normalization. Samples were subject to normalization using NormalizerR2013b_MacOSX, downloaded from the Nolan laboratory GitHub page (https://github.com/nolanlab). Normalized data underwent traditional Boolean gating in FlowJo (TreeStar), to identify singlets (191Ir+ 193Ir+) that were feasible (195Pt−).

#### Data analysis using viSNE method

Algorithm settings: Manually gated singlet (191Ir+ 193Ir+) viable (195Pt−) events were imported into Cytobank (https://www.cytobank.org) and then performed viSNE analysis. viSNE clustering analysis was conducted on 25 of 50 possible parameters, based on Abs listed in Additional file 1: Table S2. Equivalent event sampling was chosen, using 15,000 events per individual (this value based on the lowest common denominator across all samples), for a total of 125,000 events across all 12 virus-infected samples.

### Chromatin Immunoprecipitation assay (ChIP)

A549 and SPC-A-1 cells were subjected to formaldehyde incubation for 10 min to contribute to DNA-protein crosslinks. Then, cell lysates underwent sonication to get chromatin fragments of 200–300 bp and followed by immunoprecipitation with EZH2-specific antibody (5246S, CST) or IgG (2729S, CST) as a control. Subsequently, the precipitated chromatin DNA was recovered and followed by qPCR analysis.

### RNAScope

Detection of LINC00301 levels using RNAScope® probe (designed by Advanced Cell Diagnostics) and image quantification were conducted following previously described by an RNAScope® 2.5 High Definition Assay kit (Advanced Cell Diagnostics) based on the manufacturer’s procedures.

### Immunohistochemistry (IHC)

IHC for tumor tissues was conducted as described before [29–31]. Briefly, 4-μm tumor sections were subjected to incubation with anti-Ki67 (9445S, CST) or anti-HIF1α (36169S, CST) at 1:100 dilution overnight at 4 °C. Subsequently, the sections were conjugated by SignalStain® Boost IHC Detection Reagent (HRP, Rabbit, 8114S, CST) at RT for 2 hrs, followed by covering with DAB (SK-4100, Vector Laboratories, Burlingame, CA), and then slides were mounted using Vectashield mounting medium (H-1900, Vector Laboratories, Burlingame, CA). Finally, all fields were detected under Olympus 600 auto-biochemical analyzer in light microscopy (Olympus, Tokyo, Japan).

### Statistical analysis

Survival analysis on NSCLC clinical specimens was conducted by Kaplan-Meier estimate. Student’s *t*-test (two-tailed), chi-squared *t*-test, one-way ANOVA, and Mann-Whitney *U* test were subjected to analyze the in vitro and in vivo data by SPSS 23.0 software. *p* < 0.05 was deemed to be significant.

## Results

### LINC00301 is highly expressed in NSCLC and implies a poor prognosis

**Fig. 1:**
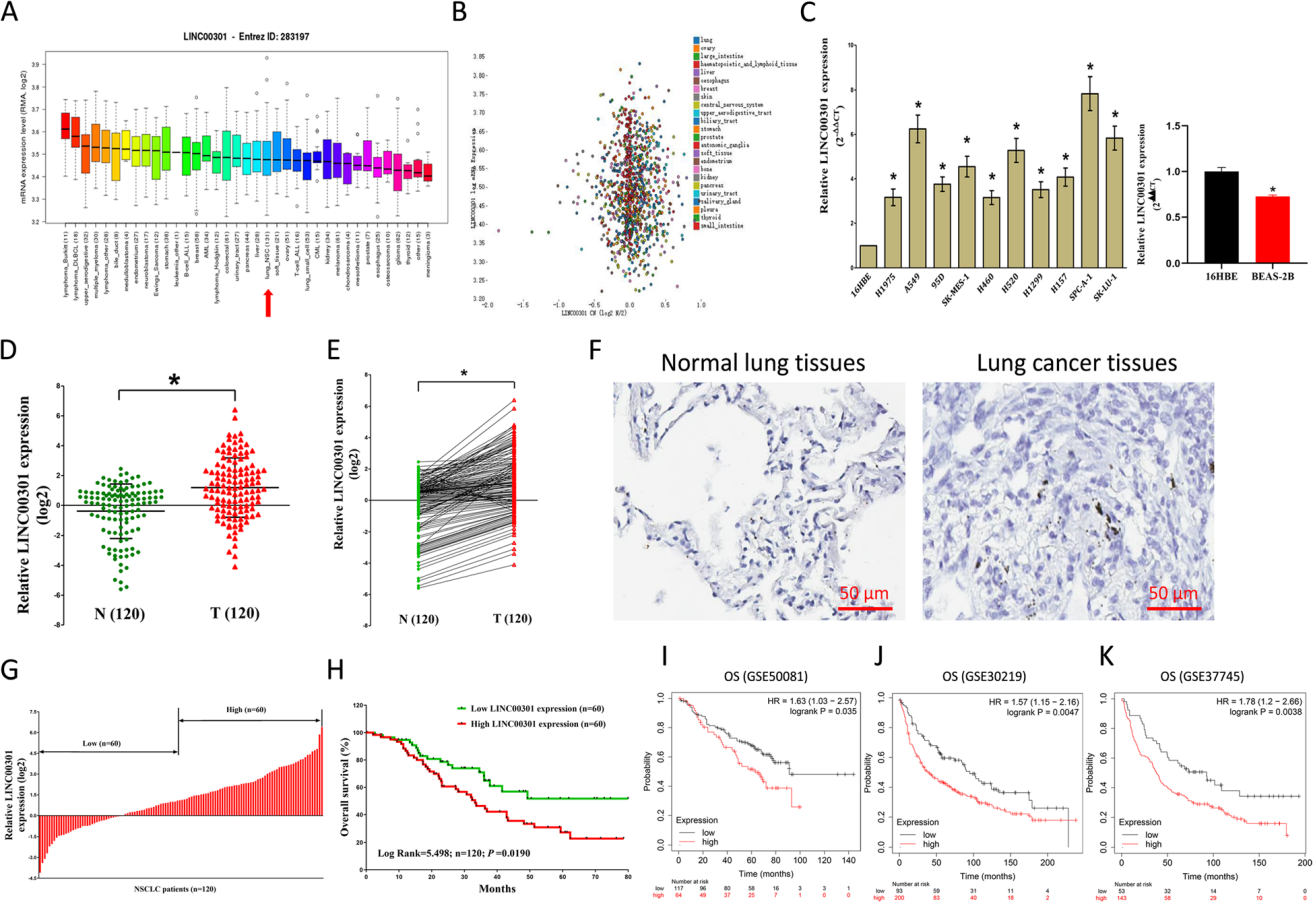
LINC00301 is up-regulated in both human NSCLC tumorous tissues and cell lines and is beneficial to NSCLC prognosis. **a**, **b** Exploring LINC00301 expression in NSCLC cell lines by assembling the Cancer Cell Line Encyclopedia (CCLE) (www.broadinstitute.org/ccle). **c** LINC00301 expression in NSCLC cell lines and 16HBE/BEAS-2B cells. **d**, **e** LINC00301 expression in NSCLC tumor tissues and adjacent normal lung tissues. **g** According to LINC00301 expression, 120 NSCLC patients were divided into high (*n* = 60) and low (*n* = 60) LINC00301 groups. **h** Kaplan-Meier survival analysis showed that high expression of LINC00301 was correlated with worse prognosis in NSCLC patients. *p < 0.05, means ± SD was shown. Statistical analysis was performed by Student’s *t*-test and the log-rank test. **i**, **j** Correlation between the LINC00301 RNA level and the survival of NSCLC patients from 2437 lung tumors using Kaplan-Meier plotter datasets (2015 version) (http://kmplot.com/analysis/index.php?p=service&cancer=lung)

We expanded the detailed annotative process of preclinical human cancer models via compiling the Cancer Cell Line Encyclopedia (CCLE) (www.broadinstitute.org/ccle), demonstrating that LINC00301 is exceedingly upregulated in cell lines of NSCLC (Fig. 1a, b). We then found that LINC00301 is higher expressed in NSCLC cell lines, including SPC-A-1, H460, SK-MES-1, 95D, A549, H157, H1299, SK-LU-1, H520, and H1975, compared with 16HBE and BEAS-2B (normal lung epithelial cells) (Fig. 1c). We examined LINC00301 expression in NSCLC via qRT-PCR and discovered that LINC00301 is significantly higher expressed in 120 NSCLC tissues compared to their counterparts (*p* < 0.05; Fig. 1d–f). Subsequently, the 120 patients with NSCLC were classified into the high group (*n* = 60) and the low group (*n* = 60) based on the mean value of LINC00301 levels (Fig. 1g). Additionally, to evaluate the clinical implication of LINC00301, we assessed the association between its expression and related clinical-pathological parameters. The results indicated that LINC00301 are astonishingly connected with lymph node metastasis (*p* < 0.0001), TNM stage (*p* = 0.0142), and tumor size (*p* = 0.0360) in NSCLC. However, LINC00301 is not correlated to extra clinical features, including age (*p* = 0.5764), gender (*p* = 0.3479), differentiation (*p* = 0.8549), smoking history (*p* = 0.5361), and histological tumor type (*p* = 0.2105) in NSCLC (Additional file 1: Table S3). Furthermore, multivariate cox regression analysis showed that higher LINC00301 expression (*n* = 60), advanced stage, and positive lymph node metastasis are unbiased prognosticators of overall survival (OS) of NSCLC patients (Additional file 1: Table S4). In addition, Kaplan–Meier analysis revealed that high LINC00301 levels are correlated to worse OS (*p* = 0.0190, log-rank test, Fig. 1h). To further investigate the essential effectiveness of LINC00301 on the survival of NSCLC patients, we evaluated the correlation between LINC00301 expression and the survival of NSCLC patients from 2437 lung cancers using Kaplan-Meier plotter (http://kmplot.com/analysis/index.php?p=service&cancer=lung) [24]. Results showed that low LINC00301 expression in NSCLC patients is significantly correlated with improved OS (Fig. 1i–k). Taken together, our data validated that higher LINC00301 expression correlates with worse prognosis, and increased LINC00301 expression may be critical in the tumorigenesis and progression of NSCLC patients.

### LINC00301 accelerates cell growth and migration/invasion and inhibits cell cycle arrest/cell apoptosis in vitro

**Fig. 2:**
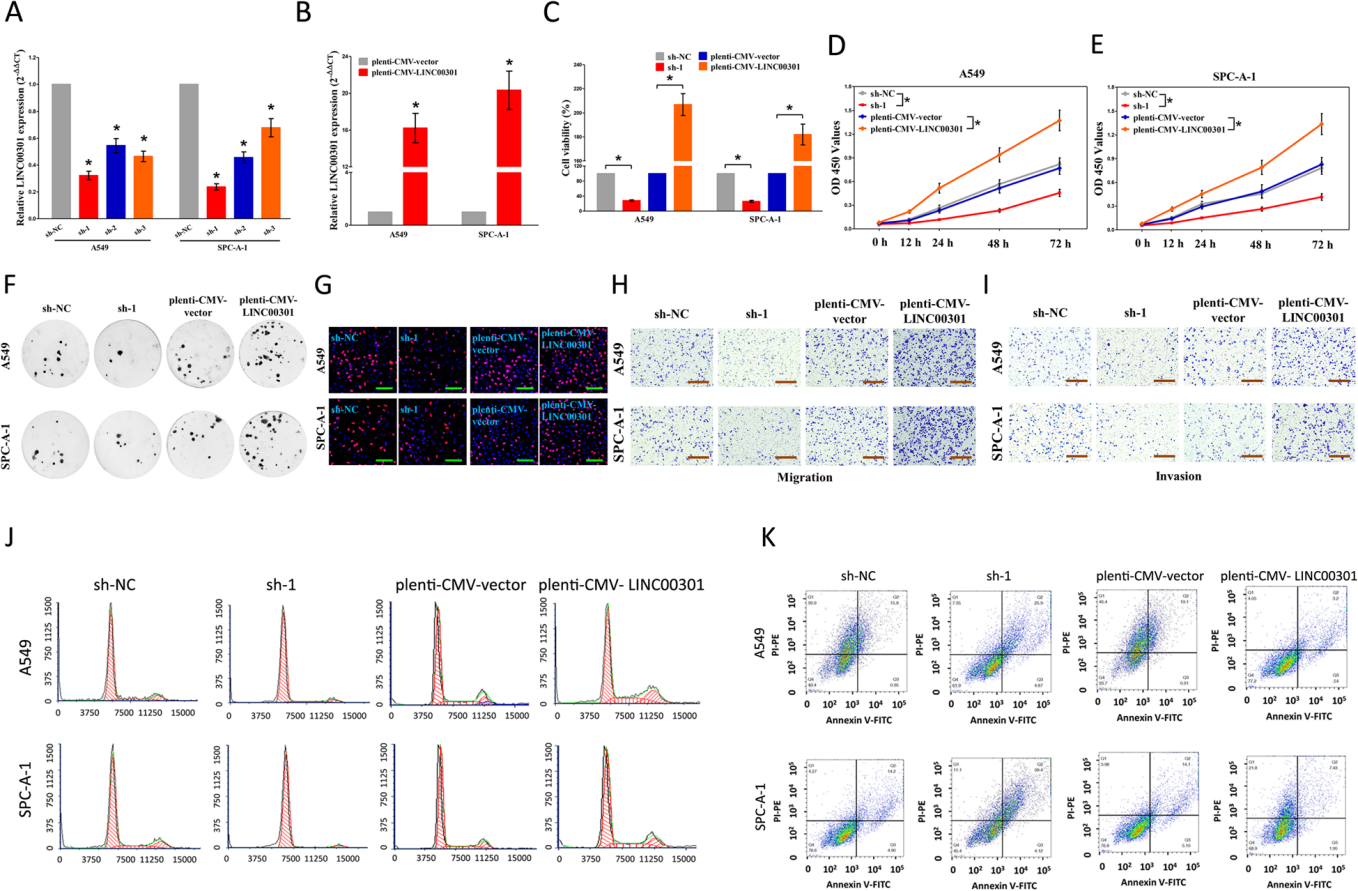
LINC00301’s effect on NSCLC cell proliferation, migration and invasion, cell cycle, and cell apoptosis in vitro. **a**, **b** The efficiency of LINC00301 overexpressed and knockout vector transfection. **c**–**e** Trypan blue staining was used to test LINC00301 on NSCLC cell vitality. And CCK8 assay indicated LINC00301 on NSCLC cell proliferation. **f** Colony formation assay (seeded at 24-well plate) indicated LINC00301 on NSCLC cell proliferation. **g** BrdU staining assay indicated LINC00301 on NSCLC cell proliferation. Bar = 100 μm. **h**, **i** Representative images of transwell migration/invasion assay for LINC00301’s role in NSCLC cell migration and invasion ability. **j**, **k** Representative images for flow cytometry analysis of A549 and SPC-A-1 cells after transfection. Cell cycle analysis discovered that LINC00301 has affected the A549 and SPC-A-1 cells proliferation (**j**), and cell apoptosis analysis showed that LINC00301 has affected the cell apoptosis of A549 and SPC-A-1 cells (**k**). *p < 0.05, means ± SD was shown. Statistical analysis was performed by Student’s *t*-test analysis

Subsequently, we explored the role of LINC00301 in NSCLC cell growth. A549, SPC-A-1, 95D, and H1299 cells were transfected with sh-NC or sh-LINC00301, i.e., sh-1, sh-2, and sh-3. The qPCR results revealed sh-1 is the most efficient shRNA in LINC00301 knockdown (KD) (Fig. 2a, Additional file 1: Figs. S1A and S2A), and thus, it was selected to represent LINC00301 KD. In addition, A549, SPC-A-1, 95D, and H1299 cells were transfected with pLenti-CMV-control and pLenti-CMV-LINC00301 (LINC00301 overexpression (OE)). The qPCR results indicated that LINC00301 OE significantly increases LINC00301 expression (Fig. 2b, Additional file 1: Fig. S1B and S2B). Further, KD/OE and rescue experiments for the *LINC00301* gene were conducted in four NSCLC cell lines (A549, SPC-A-1, 95D, and H1299 cells) to confirm the efficiency for LINC00301 KD/OE vectors (Additional file 1: Figs. S1A-B).

To identify whether LINC00301 facilitates cell growth in A549, SPC-A-1, 95D or H1299 cells, colony formation assays, CCK8, trypan blue staining, and BrdU staining were performed (Fig. 2c–g, Additional file 1: Figs. S2C-F). The results demonstrated that silencing LINC00301 induced a shrink of A549, SPC-A-1, 95D, and H1299 cell growth compared to their counterparts (Fig. 2c–g, Additional file 1: Figs. S2C-F). However, LINC00301 OE significantly increased A549, SPC-A-1, 95D, and H1299 cell growth than their counterparts (Fig. 2c–g, Additional file 1: Figs. S2C-F). These findings demonstrated LINC00301 considerably accelerates NSCLC cell growth.

Furthermore, we investigated the effectiveness of LINC00301 in A549, SPC-A-1, 95D, and H1299 cell migration and invasion. Silencing LINC00301 suppressed cell growth, migration, and invasion, in contrast to the sh-NC group (Fig. 2h, i, Additional file 1: Figs. S2G-H). In detail, LINC00301 KD suppressed 65–70% of the migratory activity of A549, SPC-A-1, 95D, and H1299 cells and reduced 47–56% of their inhibitory activity (Fig. 2h, i, Additional file 1: Figs. S2G-H). However, LINC00301 OE significantly increased A549, SPC-A-1, 95D, and H1299 cell migration and invasion than their counterparts (Fig. 2h, i, Additional file 1: Figs. S2G-H). In summary, the results indicated that LINC00301 markedly accelerates cell migration and invasion motility in NSCLC.

We further tested the function of LINC00301 in the cell cycle and apoptosis of NSCLC cells. Our results showed that silencing LINC00301 facilitates cell cycle arrest and apoptosis, whereas LINC00301 OE represses these processes in A549, SPC-A-1, 95D, and H1299 cells (Fig. 2j, k, Additional file 1: Figs. S2I-J). These findings confirmed that LINC00301 could significantly impede cell cycle arrest and apoptosis in NSCLC.

### LINC00301 facilitates tumor growth and accumulates Treg infiltration in vivo

**Fig. 3:**
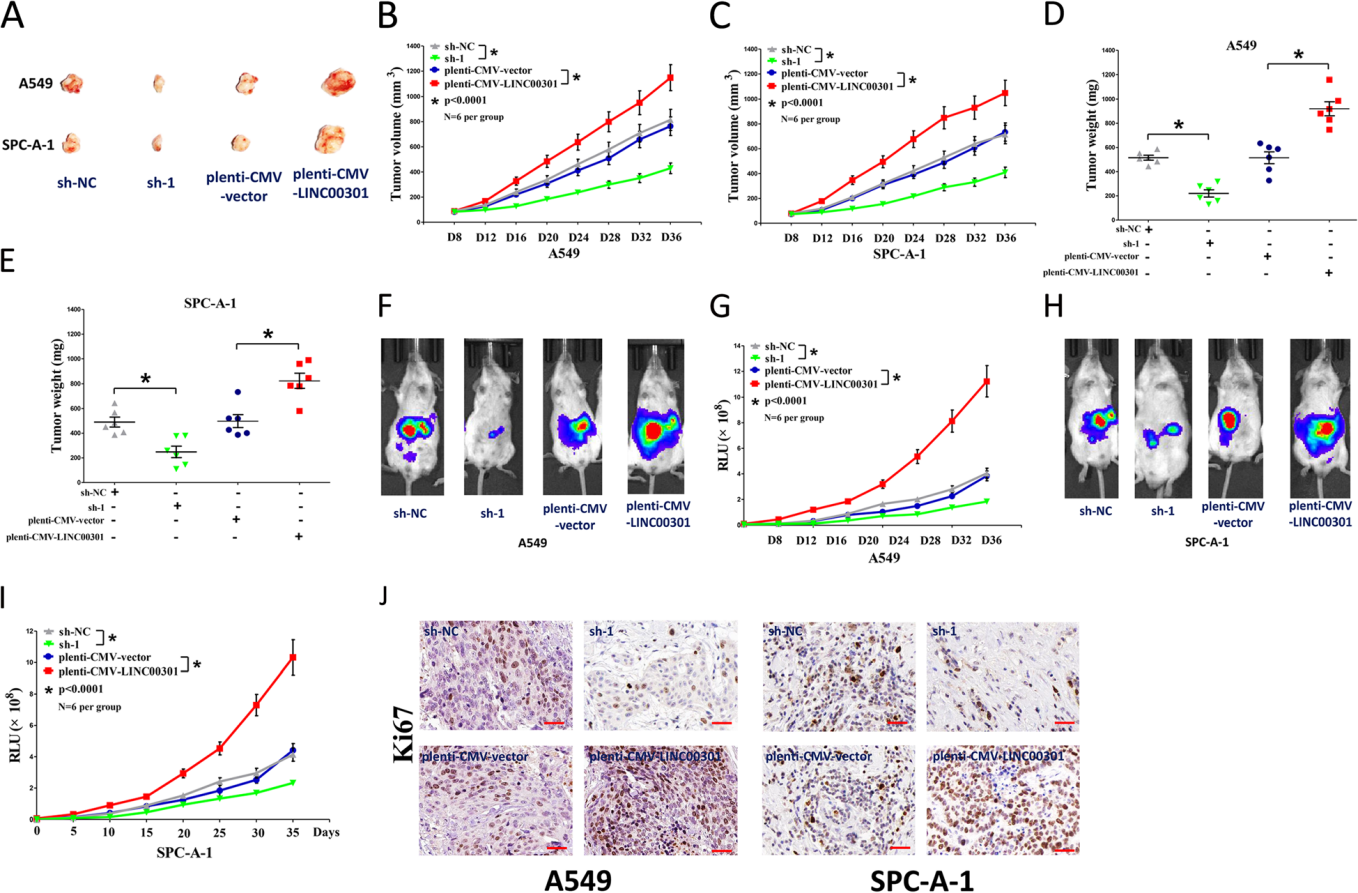
LINC00301 promotes tumor growth in vivo. **a** Representative images tumors separated from nude mice. **b**, **c** Tumor volume was measured in nude mice. **d**, **e** Tumor weight was examined in nude mice. Each group contained six mice (*n* = 6). **f–i** Representative bioluminescence imaging (BLI) images and quantification of BLI in the tumor regions for nude mice. Data were shown as the mean ± SD; * *p* < 0.05, in comparison to the sh-NC or pLenti-CMV-control group. **j** Representative images of Ki-67 staining in tumors isolated from the nude mice. Bar = 50 μm. Assays were conducted in triplicate. *p < 0.05, means ± SD was shown. Statistical analysis was subjected to Student’s *t*-test

To identify the oncogenic roles of LINC00301 in vivo, we established the BALB/c nude mice xenograft model with A549 and SPC-A-1 cells. Tumor volume and weight in LINC00301 KD nude mice were significantly repressed (~ 40% reduction in tumor weight for A549 cells and ~ 37% decrease in tumor weight for SPC-A-1 cells) compared to those of sh-NC treated mice (Fig. 3a–e). By contrast, tumor volume and weight of LINC00301 OE nude mice were noticeably increased (~ 2.5-fold increase in tumor weight for A549 cells and ~2.8-fold increase in tumor weight for SPC-A-1 cells) compared to pLenti-CMV-NC treated mice (Fig. 3a–e). To deeply study the validity of LINC00301 on tumorigenesis in vivo, BALB/c nude mice were injected with A549 and SPC-A-1 cells with stable transfection of pLenti-CMV-LUC-NC, pLenti-CMV-LUC-LINC00301, pLKO.1-DEST-LUC-NC, and pLKO.1-DEST-LUC-sh-LINC00301. LINC00301 KD significantly repressed tumor growth in vivo, and LINC00301 OE markedly facilitated tumor growth in vivo (Fig. 3f–i), suggesting that LINC00301 strikingly accelerated the A549 and SPC-A-1 cells tumorigenicity in the nude mice. Additionally, IHC conducted in tumors isolated from nude mice revealed that the number of Ki-67-positive cells was more or less in the LINC00301 OE- or KD-treated groups, respectively, than their counterparts (Fig. 3j). These results indicated that LINC00301 markedly accelerated tumorigenic capacity in mouse models of human NSCLC.

**Fig. 4:**
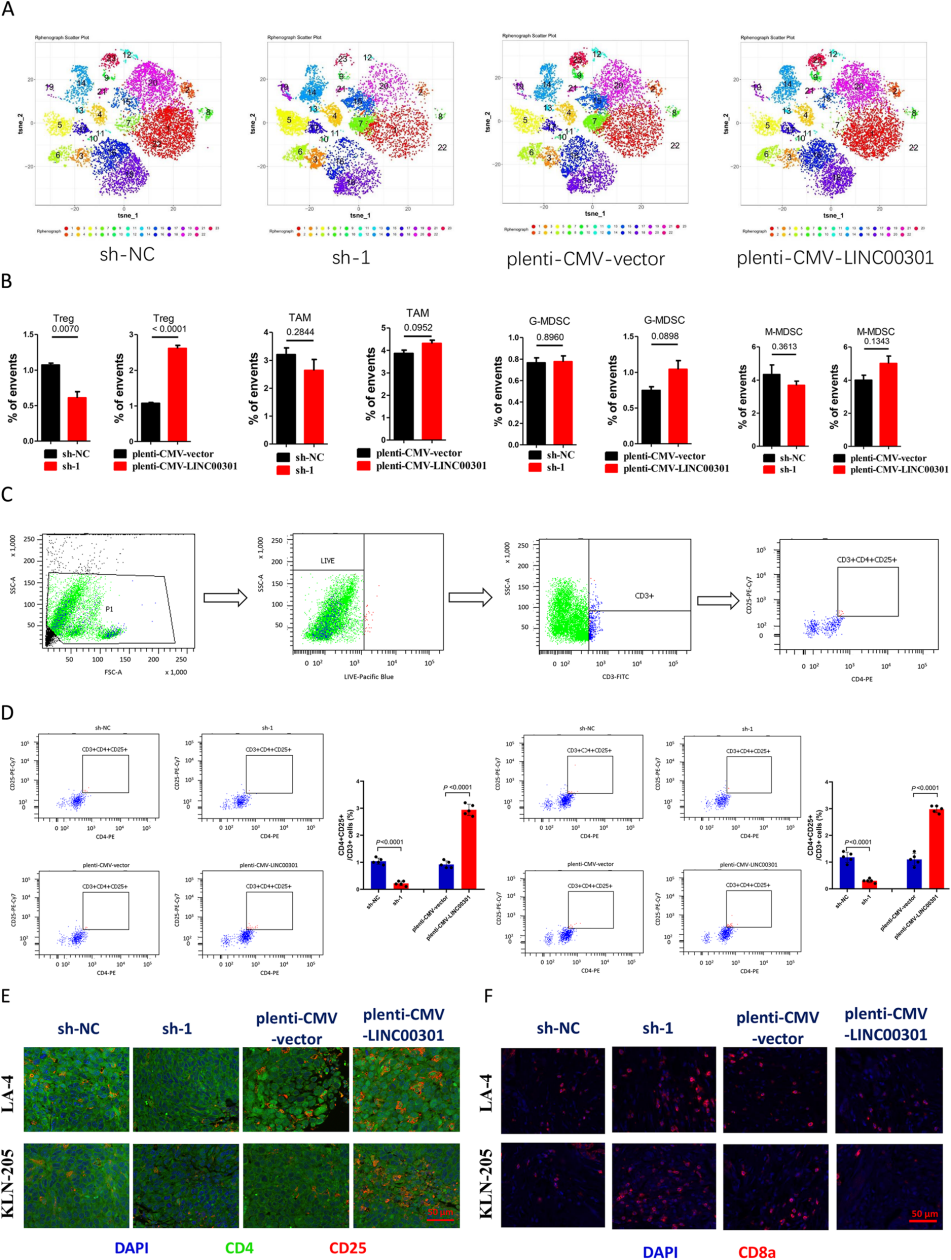
LINC00301 facilitates regulatory T cells infiltration in tumors isolated from NSCLC cell lines planted C57BL/6 J mice. **a**, **b** Representing images for CyTOF analysis of tumors by PhenoGraph analysis, identifying 22 clusters, colored by cluster identification numbers, and plotted by tSNE1 and tSNE2, clarified various clusters with obvious alterations between groups. **c** Flow cytometry gating method used in the analysis of CD3^+^/CD4^+^/CD25^+^ regulatory T cells infiltration was presented. Shown is a pLenti-CMV-vector tumor cell preparation. Following a preliminary assessment of a lymphocyte gate according to forward versus side scatter, feasible cells (FITC positive) were identified as CD3^+^ cells. The resulting CD3^+^ population was following gated according to positivity for CD4 ^+^/CD25^+^. The frequencies of cells in these populations were normalized by background values and then submitted and evaluated directly. **d** Flow cytometry measurement of percentage of CD3^+^/CD4^+^/CD25^+^ Treg cells. **e** Representative images of CD4 and CD25 of tumors isolated from sh-NC, sh-1, pLenti-CMV-vector, or pLenti-CMV-LINC00301 treated LA-4 and KLN 205 cells planting in C57BL/6 J mice (*n* = 5 animals for each group). **f** Representative images of CD8 of tumors isolated from sh-NC, sh-1, pLenti-CMV-vector, or pLenti-CMV-LINC00301-treated LA-4 and KLN 205 cells planting in C57BL/6 J mice (*n* = 5 animals for each group). Scale bar = 50 μm, *p* values were established by unpaired two-tailed Student’s *t*-test

Tumor immune environment is widely involved in tumor growth and development of different cancer types, including NSCLC. In the last 10 years, the introduction of immune checkpoint inhibitors (ICI) into the treatment of NSCLC has transformed the therapeutic landscape in this recalcitrant disease. In our study, we found that LINC00301 is higher expressed and exerted oncogenic role in NSCLC. So, we further tried to figure out whether LINC00301 might be used to identify patients that most likely to respond to ICIs. Hence, we examined the roles of LINC00301 OE and KD of LA-4 and KLN 205 cells on immune cell infiltration in tumors isolated from C57BL/6 J mice. First, tumors were isolated and digested into single cells and then applied for cytometry by time of flight (CyTOF) analysis using 22 types of antibodies, including CD45, CD3, CD4, CD25, FOXP3, CD19, CD11b, Ly-6G/6C, CD56, and Arg-1. And our data indicated that the LINC00301 OE group showed higher CD4^+^CD25^+^ Treg cell infiltration, but lower CD8^+^ T cell infiltration, while LINC00301 KD group showed lower CD4^+^CD25^+^ Treg cell infiltration, but higher CD8^+^ T cell infiltration. These findings suggested LINC00301 might be a useful biomarker for recognizing patients that most expected to respond to ICIs. Those results were confirmed by immunofluorescence staining (Fig. 4a, b, Additional file 1: Figs. S3A-B). However, LINC00301 did not seem to influence myeloid-derived suppressor cells (MDSCs; both granulocytic and monocytic) and tumor-associated macrophages (TAMs; Fig. 4a, b). To further confirm these results, we performed flow cytometry using single cells isolated from mice burden tumors and immunofluorescence (IF) staining using isolated tumors. The results demonstrated that LINC00301 KD repressed Treg but facilitated CD8^+^ T cell infiltration while LINC00301 OE accumulated Treg but suppressed CD8^+^ T cell infiltration in LA-4/KLN-205 burdened tumors in C57BL/6 J mice (Fig. 4c–f), suggesting LINC00301 exerts an immune-suppressive role in NSCLC by recruiting Treg cells.

Transforming growth factor-beta 1(TGF-β1), encoded by the *TGFB1* gene, is an essential pleiotropic, immunoregulatory cytokine. It could use distinctive signaling mechanisms in lymphocytes to modify T cell homeostasis, regulatory T cell (Treg), and effector T cell function and also involve in tumorigenesis. It is well known that TGF-β drives the development of CD4^+^Foxp3^+^ Tregs [32]. To identify how LINC00301 regulates CD4^+^Foxp3^+^ Tregs, we first examined TGF-β1 levels in the culture supernatant of NSCLC cells and normal lung epithelial cells, and the results showed a relative TGF-β1 level (ELISA) in LA-4 and KLN-205 cells than that of in MLE-12 cells (Additional file 1: Fig. S3C), and also LA-4 and KLN-205 cells showed a relatively higher TGF-β1 mRNA level than that of in MLE-12 cells (Additional file 1: Fig. S3D). Furthermore, TGF-β1 level was also shown to be highly expressed in LINC00301 OE-treated LA-4 and KLN-205 cells than that of counterparts (Additional file 1: Figs. S3E-F), while it was lowly expressed in LINC00301 KD-treated LA-4 and KLN-205 cells than that of counterparts (Additional file 1: Figs. S3D-E). Hence, we concluded that LINC00301 facilitated lung tumor secreting TGF-β1 to drive Treg cell infiltration and then repressed CD8^+^ T cell amount in the tumor microenvironment (TME).

### Methylation and deacetylation are not involved in LINC00301 upregulation in NSCLC

**Fig. 5:**
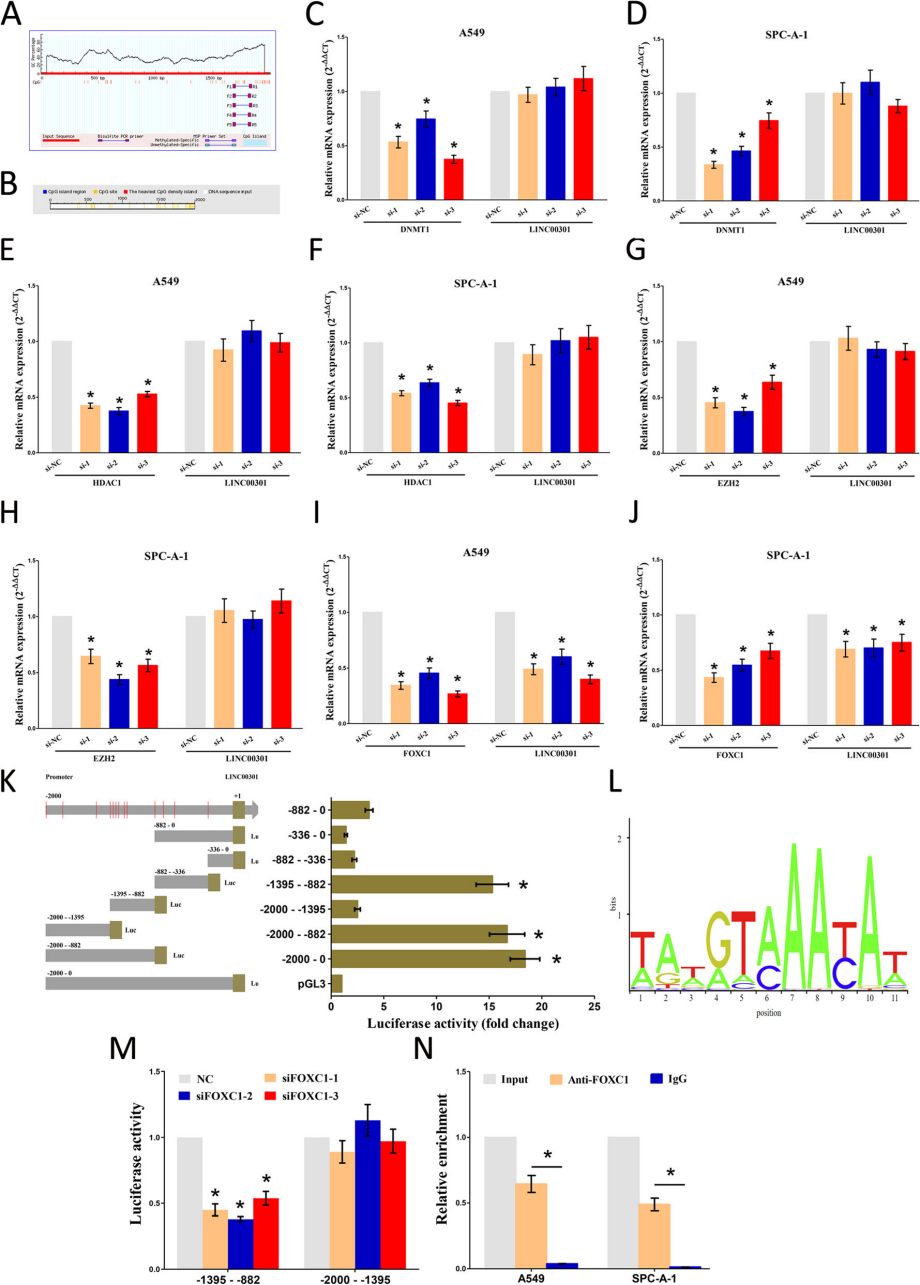
It is transcription factor FOXC1, not methylation nor deacetylation, regulates the expression of LINC00301. **a**, **b** Prediction of CpG islands in *LINC00301* promoter region by analyzing the sequences of *LINC00301* promoter through the MethPrimer online software (http://www.urogene.org/cgi-bin/methprimer/methprimer.cgi) and DBCAT (http://dbcat.cgm.ntu.edu.tw/). **c**, **d** The role of silencing DNMT1 on the expression of LINC00301 in A549 and SPC-A-1 cells. **e-f** The role of silencing HDAC1 on the expression of LINC00301 in A549 and SPC-A-1 cells. **g**, **h** The role of silencing EZH2 on the expression of LINC00301 in A549 and SPC-A-1 cells. **i**, **j** The role of silencing FOXC1 on the expression of LINC00301 in A549 and SPC-A-1 cells. **k** Serial truncations of *LINC00301* promoter were cloned into pGL3-basic vectors, and then luciferase activity was assessed after transfecting these constructed vectors into HEK-293 T cells. **l** Doman motifs for translational factor FOXC1. **m** Co-transfection of luciferase reporter containing truncations of *LINC00301* promoter and siRNA against FOXC1 into HEK-293 T cells to test the role of FOXC1 KD on *LINC00301* promoters’ activity. **n** ChIP assay was performed to show whether FOXC1 could directly bind to the site 6 (− 1395~− 1388 nt) on the *LINC00301* promoter in NSCLC cell lines. Assays were conducted in triplicate. *p < 0.05, means ± SD was shown. Statistical analysis was performed by Student’s *t*-test

Our results showed that LINC00301 acts as a crucial player in the tumor progression of NSCLC. Consequently, we then aimed to identify the regulators for LINC00301. Chromatin methylation and deacetylation may silence or activate gene expression. Hence, we first determined whether DNA methylation can regulate LINC00301 expression. No CpG islands were found in the *LINC00301* promoter, as shown by analyzing *LINC00301* promoter sequences via the online software MethPrimer (http://www.urogene.org/cgi-bin/methprimer/methprimer.cgi) and DBCAT (http://dbcat.cgm.ntu.edu.tw/) (Fig. 5a, b). Moreover, we further analyzed the correlation of DNA methylation of LINC00301 in LUAD (*n* = 458) vs. normal (*n* = 30) and LUSC (*n* = 364) vs. normal (*n* = 41) using the SMART App (http://www.bioinfo-zs.com/smartapp/) [25] that is based on TCGA Pan-Cancer cohort of UCSC Xena public data hubs (https://xenabrowser.net). The results indicated that DNA methylation of LINC00301 showed no significant difference between tumor and normal group both in LUAD (*p* = 0.1537) and LUSC (*p* = 0.0569) (Additional file 1: Figs. S4A-B). To validate the role of DNA methylation on the regulation of LINC00301 expression, A549 and SPC-A-1 cells were transfected with small interfering RNA (siRNA) for DNA (cytosine-5)-methyltransferase 1 (DNMT1), a crucial enzyme catalyzing the transfer of methyl groups to certain CpG structures of DNA. Our results demonstrated that silencing DNMT1 did not markedly influence LINC00301 expression, indicating that DNA methylation is not involved in LINC00301 upregulation in NSCLC cells (Fig. 5c, d). To further identify whether DNA methylation is exactly not affect LINC00301 expression, we also knocked down DNMT3A and DNMT3B in A549 and SPC-A-1 cells and used qPCR to examine LINC00301 expression level. And the results showed that silence of DNMT3A and DNMT3B did not significantly influence LINC00301 expression in A549 and SPC-A-1 cells (Additional file 1: Figs. S5A-D). Additionally, we further treated NSCLC cells with 5 μM 5-azacytidine (5-AZA), an inhibitor of DNA methylation, to test whether it could influence LINC00301 expression. And our results revealed that 5-AZA treatment did not affect LINC00301 levels in NSCLC cells (Additional file 1: Fig. S5E). Those results confirmed that DNA methylation is indeed not involved in LINC00301 upregulation in NSCLC cells. Histone deacetylases (HDACs) comprise a group of enzymes that eliminate acetyl groups from a histone, benefiting histones to tightly wrap with DNA. LINC00301 levels were not enhanced after hindering HDAC1 in A549 and SPC-A-1 cells, suggesting that deacetylation is not involved in the upregulation of LINC00301 in NSCLC (Fig. 5e, f). To confirm whether histone deacetylase involves in LINC00301 expression, we further treated NSCLC cells with 300 nM trichostatin A (TSA), a histone deacetylase inhibitor, and our results revealed that TSA treatment did not affect LINC00301 levels in NSCLC cells (Additional file 1: Fig. S5F). Histone methylation may also influence gene transcription. A549 and SPC-A-1 cells were transfected with si-EZH2 (EZH2 is a histone-lysine N-methyltransferase enzyme). Silencing EZH2 did not markedly influence LINC00301 expression, indicating that histone methylation is not involved in LINC00301 upregulation in NSCLC cells (Fig. 5g, h). In addition, to further identify whether histone methylation did exactly not affect LINC00301 expression, we knocked down LSD1 in A549 and SPC-A-1 cells, using qPCR to examine LINC00301 expression level. And the results showed that silence LSD1 did not significantly influence LINC00301 expression in A549 and SPC-A-1 cell (Additional file 1: Figs. S5G-H). In summary, our findings preliminarily demonstrated that methylation and deacetylation are not participating in the upregulation of LINC00301 in NSCLC.

### Transcription factor (TF) FOXC1 regulates LINC00301 expression

TFs are a fundamental player in regulating gene expression. To further identify the upstream of LINC00301, we first predicted the potential TFs that might interact with *LINC00301* promoters by the JARSPAR online databases (http://jaspar.genereg.net/). The results revealed that FOXC1, upregulated in NSCLC, was involved in those TFs. qPCR results demonstrated that silencing FOXC1 significantly contributed to LINC00301 downregulation (Fig. 5i, j).

To confirm that LINC00301 is a transcriptional target of FOXC1, we cloned serialized truncations of *LINC00301* promoters into the pGL3-basic vector and measured the luciferase activity after transfecting them into HEK-293 T cells. Our results demonstrated that the top two luciferase activities were presented in − 1395 to − 882 nt and − 2000 to − 1395 nt (Fig. 5k, l). These results indicated that the two fragments contain regulatory elements are crucial for LINC00301 transcription. Subsequently, we co-transfected the two luciferase-reporter vectors and si-FOXC1 into HEK-293 T cells, respectively. FOXC1 KD considerably diminished the luciferase activity of − 1395 to − 882 nt fragment. Nevertheless, the luciferase activity in the reporter containing − 2000 to − 1395 nt of *LINC00301* promoters was unaffected (Fig. 5m), which suggests that the regions between − 1395 to − 882 nt on the *LINC00301* promoter are responsible for the FOXC1-induced LINC00301 activation. Furthermore, the sequence analysis of − 1395 to − 882 nt fragment revealed six presumed FOXC1 binding sites located at − 1218 to − 1211 nt (site 1), − 1243 to − 1236 nt (site 2), − 1310 to − 1303 nt (site 3), − 1314 to − 1307 nt (site 4), − 1354 to − 1347 nt (site 5), and − 1395 to − 1388 nt (site 6). To clarify which FOXC1 binding sites are responsible for the FOXC1-induced transcriptional activation of LINC00301, chromatin immunoprecipitation (ChIP) assay was conducted to identify that FOXC1 could directly bind to site 6 (− 1395 to − 1388 nt) on the *LINC00301* promoter in NSCLC cell lines (Fig. 5n). Our results revealed that LINC00301 upregulation is indeed mediated by FOXC1 in NSCLC.

### Localization of LINC00301 in NSCLC cells and its role in histone modification

**Fig. 6:**
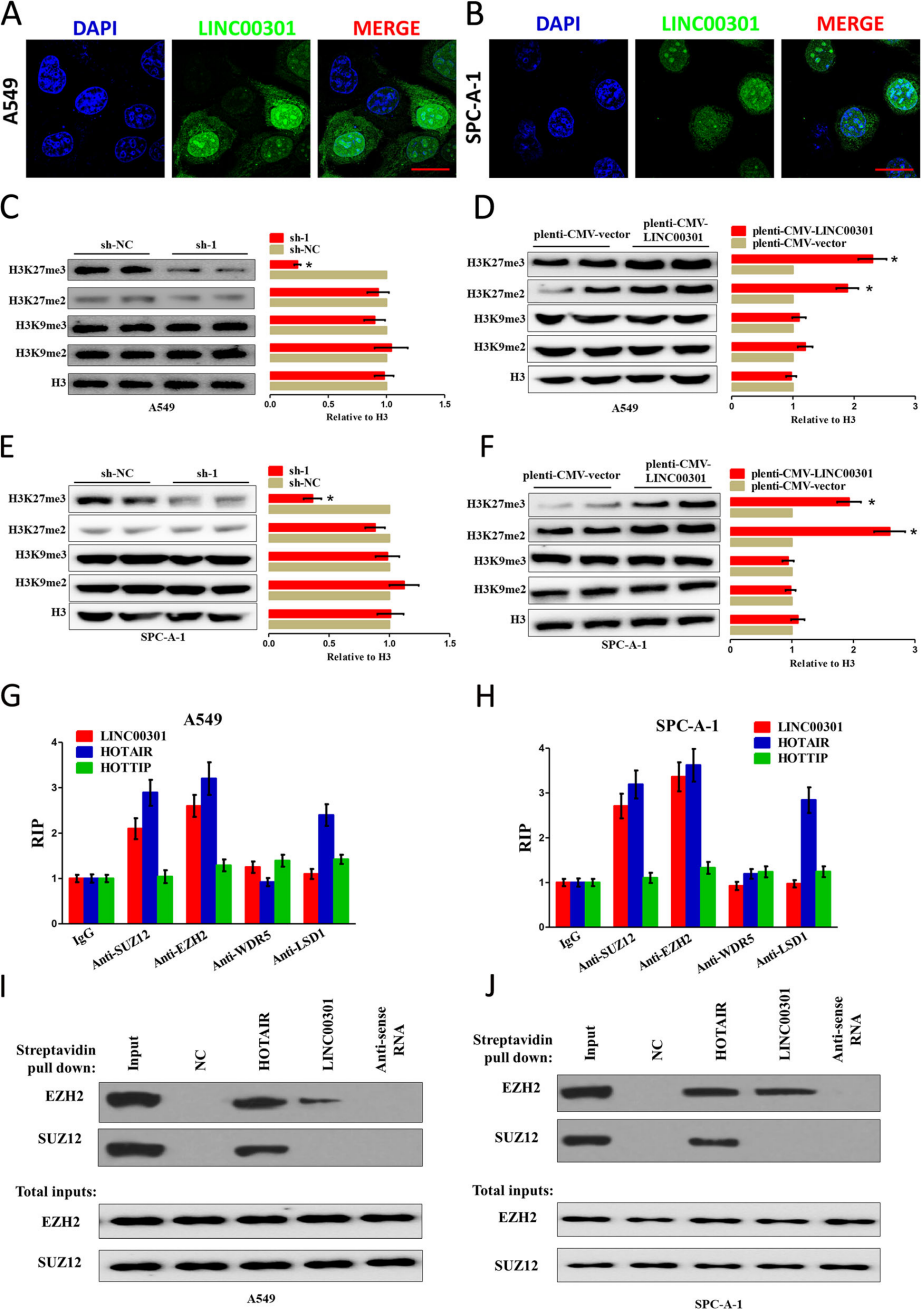
LINC00301 positively controls H3K27 trimethylation through interacting with the catalytic subunits for PRC2. **a**, **b** RNA FISH assay of LINC00301 in the location at A549 and SPC-A-1 cells. Bar = 30 μm. **c–f** Representative western blot analysis (left) and quantification of H3 methylation (right), as shown, in A549 and SPC-A-1 cells in LINC00301 KD or OE groups. Methylated H3 level was normalized to total H3 level. Data were shown as mean ± SD. * *p* < 0.05 by Student’s *t*-test (versus sh-NC or pLenti-CMV-vector). **g**, **h** RIP assay using indicated antibodies, followed by RT–qPCR for LINC00301, HOTAIR, and HOTTIP. Values were normalized to the related IgG RIP groups. Data were shown as mean ± SD. Assays were conducted in triplicate. **i**, **j** Representative images of western blot analysis (of *n* = 2) of a biotinylated RNA streptavidin pull-down assay. Biotinylated full-length (WT) LINC00301 was incubated with A549 or SPC-A-1 cell lysates in RNA-protein binding buffer, followed by streptavidin bead pull-down. PRC2 components such as SUZ12 and EZH2 were exposed by IB in the pull-down products (top set) and total inputs (bottom set). Assays were conducted in triplicate. *p < 0.05, means ± SD was shown. Statistical analysis was performed by Student’s *t*-test

Our results showed that LINC00301 exhibits oncogenic roles both in vitro and in vivo of NSCLC. However, the detailed molecular mechanism contributing to these roles has not been investigated yet. To clarify the molecular mechanism of LINC00301 referred to NSCLC cells, we first evaluated the subcellular localization of LINC00301 in NSCLC cells. FISH was executed to identify the localization of LINC00301 in A549 and SPC-A-1 cells. And the results indicated that LINC00301 locates in both cytoplasm and nucleus, but the ratio of LINC00301 in the nucleus is much higher compared to that in the cytoplasm (Fig. 6a, b). Hence, we suspected that LINC00301 may act as an oncogene through the nucleus and cytoplasm pathways.

Histone methylation is implicated in transcriptome reprogramming during NSCLC progression [33–35]. We observed that LINC00301 deficiency in NSCLC cells specifically decreased di- and trimethylation at H3K27 without affecting the levels of di- and trimethylation at H3K9 sites (Fig. 6c, e, Additional file 1: Figs. S6A and S6C). However, LINC00301 overexpression specifically increased H3K27 trimethylation without a detectable influence on other histone methylations (Fig. 6d, f, Additional file 1: Figs. S6B and S6D). Furthermore, we have also examined whether LINC00301 OE/KD affected H3K4 di-/trimethylation, H4K20 trimethylation, and H3K79 trimethylation level, and the results showed no change (data not shown). These results indicate a specific but positive regulation of H3K27 methylation by LINC00301 in NSCLC.

Di- and trimethylation at H3K27 are generally catalyzed by the histone methyltransferase polycomb repressive complex 2 (PRC2), an eminent molecular target of numerous regulatory lncRNAs [16, 33, 34]. Through RIP assay, we discovered a remarkable enrichment of LINC00301 in the interactome with the PRC2 elements EZH2 and SUZ12 but not in those with the TrxG/MLL components LSD1 and WDR5 in A549 and SPC-A-1 cells (Fig. 6g, h). As a positive control, HOTAIR was found to interact with both PRC2 TrxG/MLL components, as previously reported [16, 33, 34]. To deeply validate the function of LINC00301-PRC2 interaction, RNA pull-down assays using streptavidin-conjugated beads were conducted to verify that biotinylated LINC00301 binds with EZH2 but not with SUZ12 in NSCLC cells (Fig. 6i, j, Additional file 1: Figs. S5E and S5F).

**Fig. 7:**
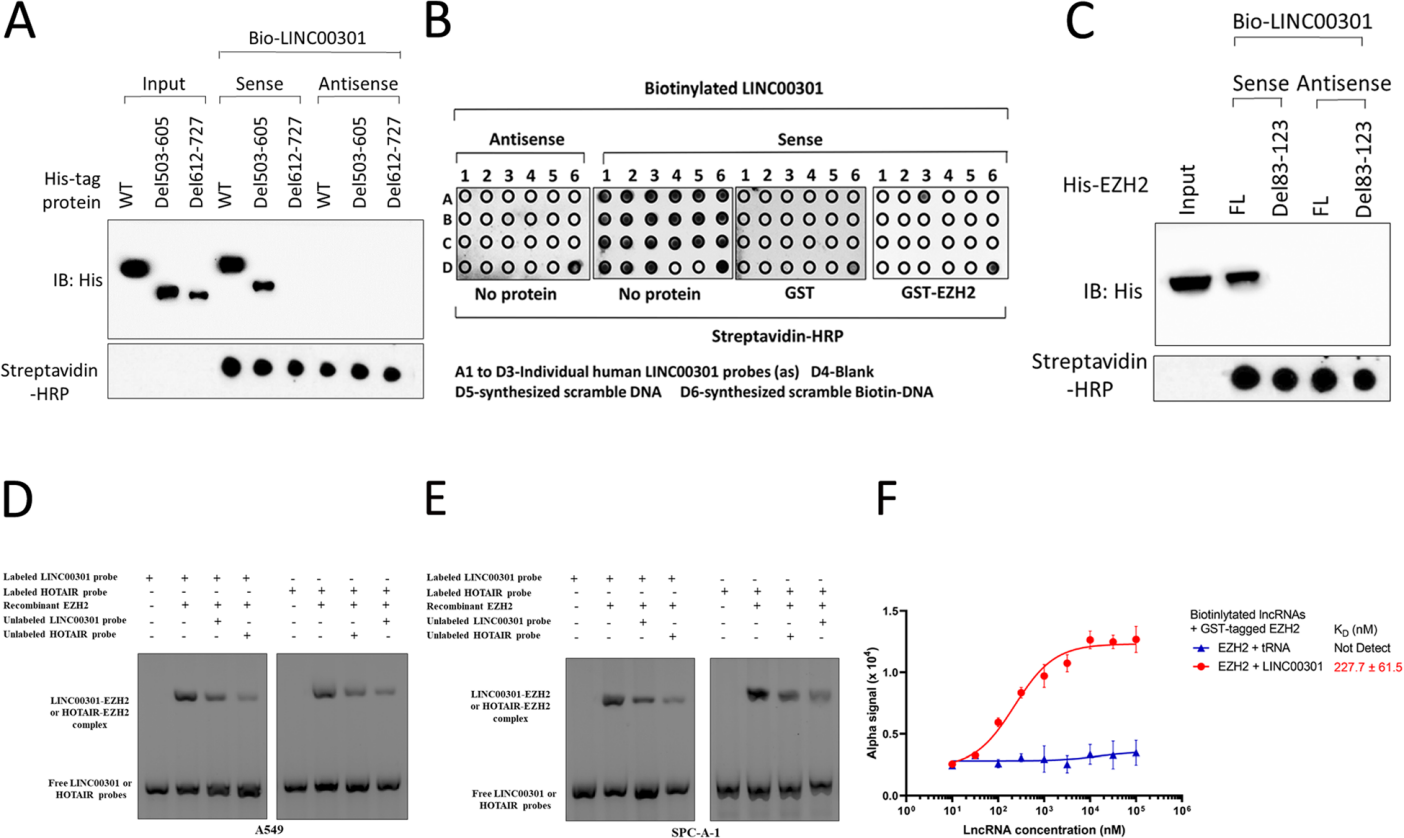
LINC00301 (83–123 nt) binds directly with EZH2 (612–717 aa) protein. **a** Immunoblot detection of his-tagged EZH2 (WT versus domain truncation mutants) retrieved by in vitro-transcribed biotinylated LINC00301. **b** In vitro RNA-protein binding pull-down, coupled by dot blot assays. **c** IB detection of proteins retrieved by in vitro-transcribed biotinylated LINC00301 (WT versus Δ83–Δ123). **d**, **e** RNA-EMSA of recombinant EZH2 purified from A549 and SPC-A-1 cells binding to BCAR4 83–123 nt. **f** Saturation curve KD determination of interaction between specified lncRNAs and recombinant GST-tagged EZH2 (*n* = 3 of independent experiments). Assays were performed in triplicate

Furthermore, we predicted the molecular structure of LINC00301 using the RNAfold web server (http://rna.tbi.univie.ac.at/cgi-bin/RNAWebSuite/RNAfold.cgi; Additional file 1: Fig. S7A). And then we predicted the interaction between LINC00301 and EZH2 using catRAPID (http://service.tartaglialab.com/page/catrapid_group). Those bioinformatics results showed that LINC00301 might directly bind with EZH2 at a relevant high potential (Additional file 1: Figs. S7B–S7G). In particular, EZH2 protein has two domains: CXC (503–605 amino acid [aa]) and SET (612–727 aa). To determine which domain LINC00301 directly binds with, we conducted protein domain mapping. The results revealed that LINC00301 directly binds with the 612–727 aa region of EZH2 (Fig. 7a). To map the LINC00301 sequence motifs responsible for EZH2 binding, we conducted in vitro RNA pull-down coupled by dot blot assay. The motif sequences of LINC00301 bound and protected by EZH2 is recognized to comprise 83–123 nt (Fig. 7b). Nevertheless, the glutathione S-transferase (GST) protein presented no particular attachment to any regions of LINC00301 (Fig. 7b). Eliminating the related sequences of LINC00301 (Δ83–Δ123) obliterated its interaction with EZH2 (Fig. 7c). To further validate the direct binding sites between LINC00301 with EZH2, we conducted RNA EMSA and identified a certain complex between LINC00301 and the recombinant EZH2 separately purified from A549 and SPC-A-1 (Fig. 7d, e). The incubation of the LINC00301 RNA probe (83–123 nt) or HOTAIR probes with recombinant EZH2 resulted in specific gel retardation (Fig. 7d, e). LINC00301 or HOTAIR EZH2-binding motifs effectively competed for this interaction, reinforcing the concept of a shared binding entity between the two lncRNAs (Fig. 7d, e). Nevertheless, the EZH2-binding motif from LINC00301 exhibited lower binding affinity compared with the motif from HOTAIR (Fig. 7d, e). Alpha assay was conducted to quantify the interaction between LINC00301 and EZH2 using biotinylated LINC00301 and glutathione S-transferase (GST)-tagged EZH2 as the donor and acceptor, respectively. Approximately 227.7 nM of LINC00301 reached 50% maximal binding with EZH2 (Fig. 7f). Therefore, we concluded that LINC00301 directly binds to EZH2 via a specific region.

### LINC00301 recruits EZH2 and mediates H3K27me3 at *EAF2* promoter to repress EAF2 transcription

**Fig. 8:**
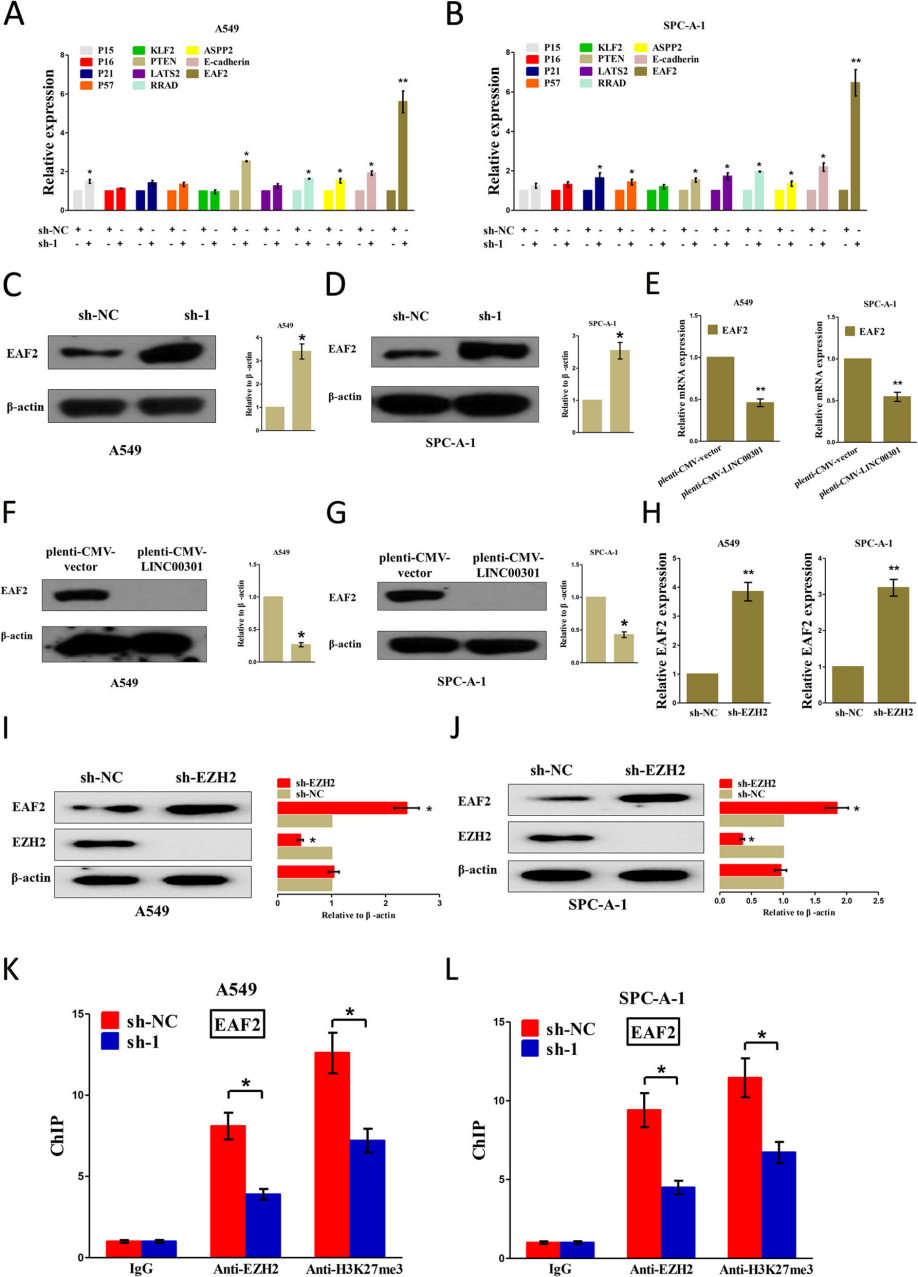
LINC00301 recruits EZH2 and mediates H3K27me3 to *EAF2* promoter to repress EAF2 transcription. **a**, **b** qPCR was conducted to test a series of tumor-suppressive mRNA levels in A549 and SPC-A-1 cells with LNC00301 KD. **c**, **d** IB tested EAF2 level in LINC00301 KD A549 and SPC-A-1 cells. **e** qPCR was conducted to test the EAF2 mRNA level in A549 and SPC-A-1 cells with LNC00301 OE. **f**, **g** IB tested EAF2 level in LINC00301 overexpressed A549 and SPC-A-1 cells. **h** qPCR was conducted to test the EAF2 mRNA level in A549 and SPC-A-1 cells with sh-EZH2. **i**, **j** IB tested EAF2 level in EZH2 overexpressed A549 and SPC-A-1 cells. **k**, **l** ChIP–qPCR analysis of EZH2 occupancy H3K27me3 binding in the *EAF2* promoter after LINC00301 KD. Assays were subjected to triplicate examination. *p < 0.05, means ± SD was exhibited. Statistical analysis was performed based on Student’s *t*-test

Experiments above have confirmed that LINC00301 binds directly with EZH2 in NSCLC cells; hence, we try to investigate their potential function and mechanism in NSCLC. Trimethylation at H3K27 is catalyzed by EZH2, which is widely reported as a molecular target of several regulatory lncRNA in NSCLC [16, 33, 34]. Herein, A549 and SPC-A-1 cells were transfected with sh-NC and sh-LINC00301, respectively. Then, qPCR was conducted to examine the mRNA levels of EZH2 target suppressive genes [20, 22, 33, 36–40], including p15, p16, p21, p57, KLF2, PTEN, LATS2, RRAD, ASPP2, E-cadherin, and EAF2. The results demonstrated that the silence of LINC00301 remarkably increased the EAF2 mRNA level in both A549 and SPC-A-1 cells (Fig. 8a, b). Western blot assay was then conducted to verify the influence of LINC00301 on the protein expression of EAF2. Results demonstrated that LINC00301 KD treatment significantly increased EAF2 protein level in A549 and SPC-A-1 cells (Fig. 8c, d). Subsequently, A549 and SPC-A-1 cells were transfected with pLenti-CMV-NC or LINC00301 OE, and the results showed that INC00301 overexpression markedly restrained the mRNA and protein levels of EAF2 in NSCLC (Fig. 8e–g).

To test whether EZH2 affected EAF2 expression in NSCLC, A549 and SPC-A-1 cells were transfected with sh-NC and EZH2 KD respectively, and then conducted qPCR and western blot assay to analyze EAF2 mRNA and protein levels. LINC00301 KD markedly increased the mRNA and protein levels of EAF2 in A549 and SPC-A-1 cells (Fig. 8h–j). To validate if EZH2 can directly bind with *EAF2* promoter regions, we constructed 4 pairs of primers targeting 2000 bp of its promoter regions. ChIP assay revealed that EZH2 can directly bind to the promoter regions of *EAF2* (Fig. 8k, l). Therefore, LINC00301 represses EAF2 expression by directly binding with EZH2 to mediate H3K27me3 at the *EAF2* promoter in NSCLC cells.

We also explored the crucial effect of EAF2 on the survival of NSCLC patients by using Kaplan-Meier plotter tools (http://kmplot.com/analysis/index.php?p=service&cancer=lung). The results showed that higher EAF2 mRNA levels in NSCLC patients significantly correlate with an improved OS, PFS, and PPS survival of patients (Additional file 1: Figs. S8A–S8C). These bioinformatic analyses further validated the tumor-suppressive role of EAF2 on NSCLC.

### LINC00301 facilitates NSCLC cell growth and migration/invasion via regulation of EAF2/VHL/HIF1α pathway

**Fig. 9:**
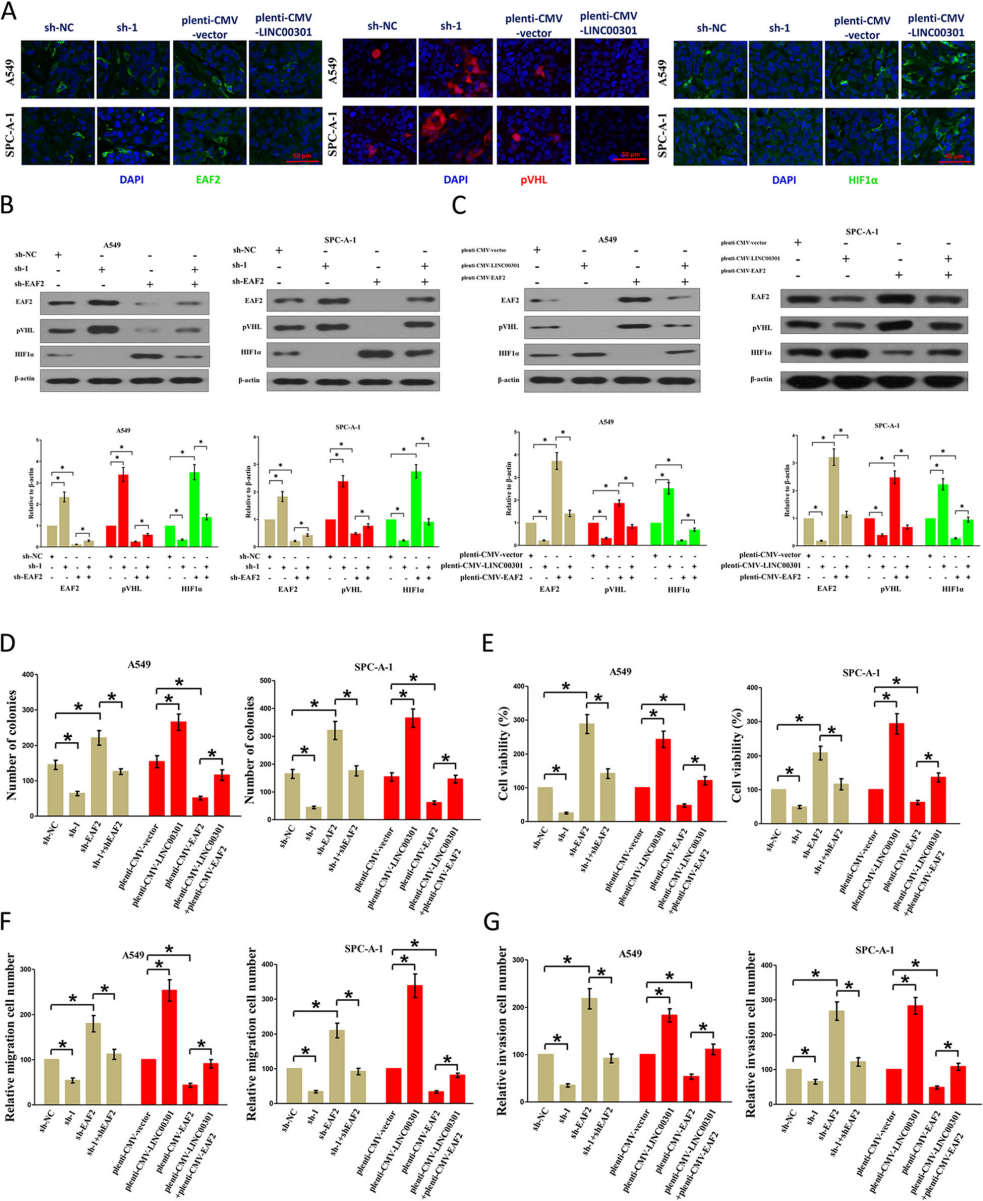
LINC00301 facilitates proliferation, migration, and invasion through regulation of EAF2/VHL/HIF1α pathway in NSCLC cells. **a** Represent images for EAF2, pVHL, HIF1α in LINC00301 KD, and LINC00301 OE A549 and SPC-A-1 cells analyzed by immunofluorescence. **b** IB tested EAF2, pVHL, HIF1α levels in sh-NC, sh-1, sh-EAF2, sh-1 + sh-EAF2 transfected A549, and SPC-A-1 cells. **c** IB tested EAF2, pVHL, HIF1α levels in pLenti-CMV-vector, pLenti-CMV-LINC00301, pLenti-CMV-EAF2 transfected in A549, and SPC-A-1 cells. **d** Colony formation assays (seeded in 6-well plate) tested cell proliferation in sh-NC, sh-1, sh-EAF2, sh-1 + sh-EAF2, pLenti-CMV-vector, pLenti-CMV-LINC00301, pLenti-CMV-EAF2 or pLenti-CMV-LINC00301 + pLenti-CMV-EAF2 transfected in A549 and SPC-A-1 cells. **e** Trypan blue staining tested cell viability in sh-NC, sh-1, sh-EAF2, sh-1 + sh-EAF2, pLenti-CMV-vector, pLenti-CMV-LINC00301, pLenti-CMV-EAF2 or pLenti-CMV-LINC00301 + pLenti-CMV-EAF2 transfected in A549 and SPC-A-1 cells. **g** Transwell migration assay tested migration activity in sh-NC, sh-1, sh-EAF2, sh-1 + sh-EAF2, pLenti-CMV-vector, pLenti-CMV-LINC00301, pLenti-CMV-EAF2 or pLenti-CMV-LINC00301 + pLenti-CMV-EAF2 transfected in A549 and SPC-A-1 cells. **f** Transwell migration assay tested migration activity in sh-NC, sh-1, sh-EAF2, sh-1 + sh-EAF2, pLenti-CMV-vector, pLenti-CMV-LINC00301, pLenti-CMV-EAF2 or pLenti-CMV-LINC00301 + pLenti-CMV-EAF2 transfected in A549 and SPC-A-1 cells. **g** Transwell invasion assay tested invasive activity in sh-NC, sh-1, sh-EAF2, sh-1 + sh-EAF2, pLenti-CMV-vector, pLenti-CMV-LINC00301, pLenti-CMV-EAF2 or pLenti-CMV-LINC00301 + pLenti-CMV-EAF2 transfected in A549 and SPC-A-1 cells. Assays were conducted in triplicate. *p < 0.05, means ± SD was shown. Statistical analysis was performed by Student’s *t*-test

The tumor suppressor EAF2 is downregulated in tumors, such as lung cancer [41]. Xiao et al. reported that EAF2 could bind to and stabilize pVHL, and then pVHL facilitates HIF1α degradation [42]. EAF2 shields cells from hypoxic-triggered cell death via interrupting the recruitment of p300 and inhibiting the activity of HIF1α [43]. The serial analysis of gene expression database shows that *EAF2* is a targeted gene for EZH2 [40]. Hence, EAF2 may be a key linking factor between EZH2 upregulation and HIF1α activation. To prove this hypothesis, A549 and SPC-A-1 cells were transfected with sh-NC, EAF2 KS, LINC00301 KD, pLenti-CMV-NC, EAF2 OE, or LINC00301 OE, respectively. The results demonstrated that LINC00301 KD treatment significantly increased EAF2 and pVHL protein expression and decreased HIF1α protein expression. LINC00301 OE treatment significantly repressed EAF2 and pVHL protein expression and increased HIF1α protein expression. EAF2 KD treatment significantly suppressed EAF2 and pVHL protein expression and increased HIF1α protein expression. EAF2 OE treatment significantly increased EAF2 and pVHL protein expression and reduced HIF1α protein levels in A549 and SPC-A-1 cells (Fig. 9a–c).

Subsequently, we examined the LINC00301/EAF2 pathway in the proliferation, migration, and invasion of NSCLC cells. The results indicated that LINC00301 KD and EAF2 OE obstructed cell proliferation, migration, and invasion. However, LINC00301 OE and EAF2 KD accelerated the proliferation, migration, and invasion of A549 and SPC-A-1 cells (Fig. 9d–g).

### HIF1α is highly expressed and closely correlated with prognosis in patients with NSCLC

HIF1α plays a crucial regulatory effect on the occurrence and development of various categories of cancers including lung cancer [26, 44, 45]. To examine the expression of HIF1α in NSCLC patients, we performed western blot and immunohistochemistry (IHC) assay. The results revealed that HIF1α was higher expressed in cancer tissues than in the paired normal lung tissues of NSCLC patients. (Additional file 1: Figs. S9A-9C).

Then, we explored the crucial effect of HIF1α in the survival of NSCLC patients by an online database (http://kmplot.com/analysis/index.php?p=service&cancer=lung). Results revealed that high HIF1α mRNA level in NSCLC patients is significantly correlated with an improved OS and PFS survival of NSCLC patients (Additional file 1: Figs. S10A-S10I).

### LINC00301’s oncogenic roles partially involve in sponging miR-1276 and then activating HIF1α

**Fig. 10:**
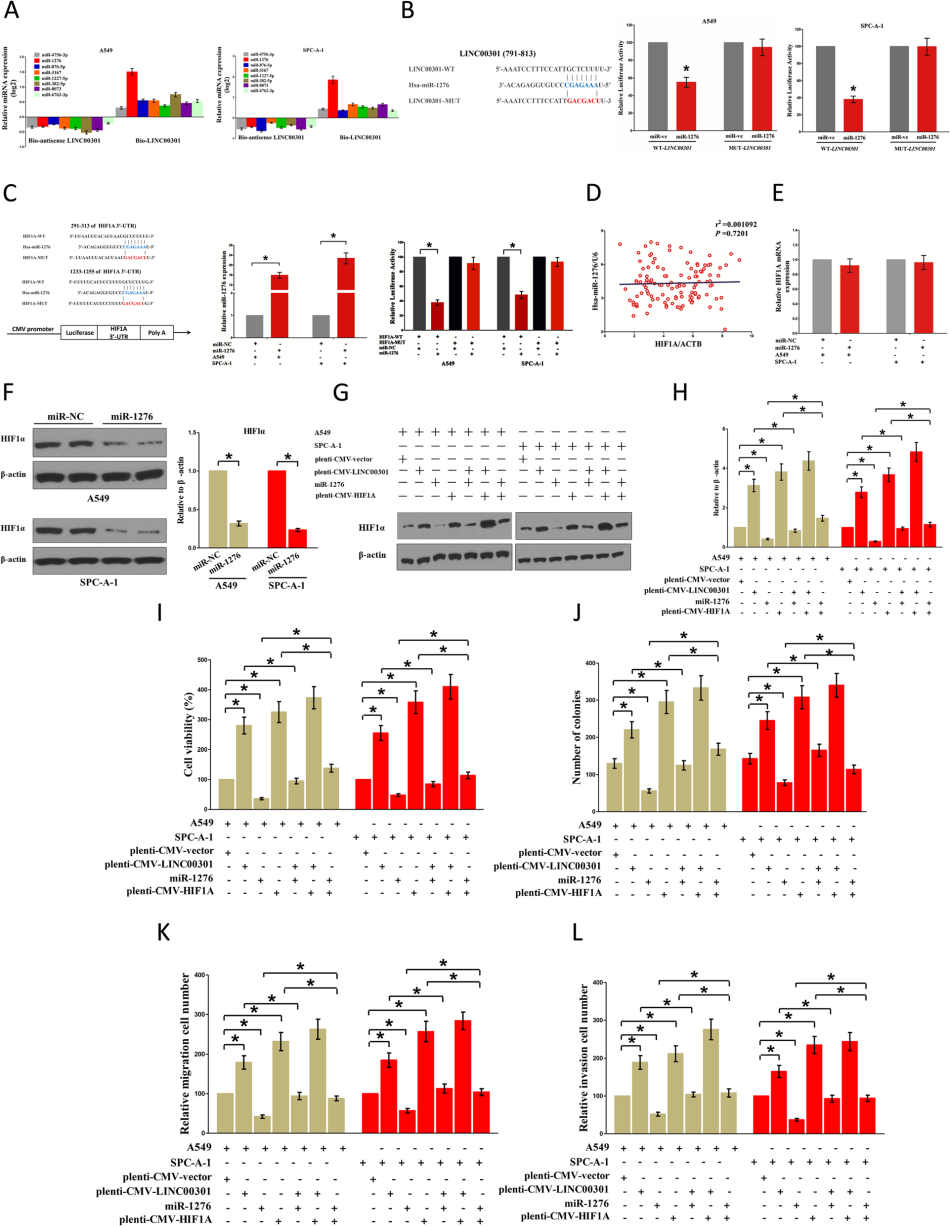
LINC00301’s oncogenic effects are also in part attributed to sponging miR-1276, and then triggering HIF1α. **a** Detection of targeted miRNAs using qPCR in the sample pulled down by biotinylated LINC00301 (sense and antisense). **b** Left: Sequence alignment of miR-1276 with the presumed binding sites within the WT or MUT LINC00301 regions. The luciferase report assay proved that miR-1276 overexpression could decrease the intensity of fluorescence in A549 (middle) and SPC-A-1 (right) cells transfected with the LINC00301-WT vector, whereas it had no impact on the LINC00301-MUT vector. **c** The 3′-UTR of HIF1A harbors 2 miR-1276 equivalent sites. Relative luciferase activity of reporter vectors containing HIF1A WT or MUT 3′UTR in A549 and SPC-1-A cells co-transfected with miR-NC or miR-1276. **d** The relationship between HIF1A mRNA expression and miR-1276 expression in NSCLC tissues. **e** The role of miR-1276 on HIF1A mRNA expression in A549 and SPC-A-1 cells. **f** The role of miR-1276 on HIF1α protein expression in A549 and SPC-A-1 cells. **g**, **h** The role of pLenti-CMV-LINC00301, miR-1276, pLenti-CMV-HIF1A, or their interaction roles on HIF1α protein expression in A549 and SPC-A-1 cells. **i** Trypan blue staining tested cell viability in pLenti-CMV-LINC00301, miR-1276, pLenti-CMV-HIF1A, or their interaction transfected in A549 and SPC-A-1 cells. **j** Colony formation assays tested cell proliferation (seeded at 6-well plate) in pLenti-CMV-LINC00301, miR-1276, pLenti-CMV-HIF1A, or their interaction transfected in A549 and SPC-A-1 cells. **k** Transwell migration assay tested migration activity in pLenti-CMV-LINC00301, miR-1276, pLenti-CMV-HIF1A or their interaction transfected in A549 and SPC-A-1 cells. **l** Transwell invasion assay tested invasive activity in pLenti-CMV-LINC00301, miR-1276, pLenti-CMV-HIF1A, or their interaction transfected in A549 and SPC-A-1 cells. Assays were conducted in triplicate. *p < 0.05, means ± SD was shown. Statistical analysis was performed by Student’s *t*-test

Given that LINC00301 is located in the nucleus (primary) and cytoplasm (secondary), we speculated that LINC00301 may also play its oncogenic role through the cytoplasm pathway. LncRNA might act as a ceRNA on the regulation of the biological effects for miRNA. To clarify the specific miRNAs interrelating with LINC00301, we evaluated the predicted results of miRDB (http://mirdb.org/cgi-bin/custom_predict/customDetail.cgi) to summarize potential miRNAs (Additional file 1: Table S5). In miRDB, miRNAs with a target score of ≥ 50 was selected. To distinguish which miRNA is the most enriched one binding with LINC00301, RNA pull-down assay was conducted by a biotin-labeled sense LINC00301 RNA. A biotin-labeled antisense LINC00301 RNA was performed as a negative control. Subsequently, qPCR was performed after precipitation. We found that miR-1276 was considerably richer in the precipitate of the sense LINC00301 RNA than that in the antisense LINC00301 RNA and other predicted miRNAs (including miR-4756-3p, miR-876-5p, miR-3167, miR-1227-5p, miR-382-5p, miR-8073, and miR-6762-3p; Fig. 10a). Additionally, a dual-luciferase reporter assay was conducted to further determine if LINC00301 is a practical target for miR-1276. Our results showed that miR-1276 significantly suppressed the luciferase activity of pmirGLO-LINC00301-WT. However, it did not affect the luciferase activity of pmirGLO-LINC00301-MUT (Fig. 10b). Those results suggested that miR-1276 could directly bind to LINC00301 at the recognized sites.

To examine the role of miR-1276 on NSCLC, we screened miRanda (http://www.microrna.org/), Targetscan (http://www.targetscan.org/vert_72/), and PicTar (https://pictar.mdc-berlin.de/) to select the probable targets for miR-1276. We clarified the top 100 underlying targets. We uncovered a well-known oncogene, HIF1α, that is highly expressed in various malignancies. Our data suggested that HIF1α may be a direct target of miR-1276 (Fig. 10c). Subsequently, dual-luciferase-reporter assays were conducted to verify if HIF1α is controlled by miR-1276. The data showed that miR-1276 inhibited luciferase activity in A549 and SPC-A-1 cells at the reporter vector with a wild type HIF1A 3′-UTR mRNA. However, no substantial inhibition was detected at the reporter vector with a mutant HIF1A 3′-UTR (Fig. 10c). Our findings revealed that the HIF1A mRNA level did not significantly correlate with miR-1276 expression in the NSCLC samples (*r*^2^ = 0.001092, *p* = 0.7201; Fig. 10d). Furthermore, miR-1276 did not affect HIF1A mRNA expression level (Fig. 10e) but suppressed HIF1α protein expression level (Fig. 10f). This finding indicated that miR-1276 influences HIF1α protein expression at the posttranscriptional level.

Additionally, we explored the roles of LINC00301 and miR-1276 on HIF1α expression. Those results validated that miR-1276 treatment suppressed HIF1α protein expression. LINC00301 OE and HIF1A OE treatment significantly enhanced HIF1α protein levels in A549 and SPC-A-1 cells (Fig. 10g, h), separately. Nevertheless, when A549 and SPC-A-1 cells were treated with LINC00301 OE plus miR-1276 mimic, the advantageous role of LINC00301 on HIF1α protein level was repressed by miR-1276. Furthermore, the negative efficiency of miR-1276 was alleviated by LINC00301 OE (Figs. 10g, h). Moreover, when A549 and SPC-A-1 cells were treated with HIF1A OE plus miR-1276 mimic, the favorable role of HIF1A in HIF1α protein levels was suppressed by miR-1276, and the negative role of miR-1276 was alleviated by HIF1A OE (Figs. 10g, h). Our results demonstrated that the oncogenic function of LINC00301 is partially attributed to the miR-1276-HIF1α axis in NSCLC.

Next, we investigated the function of the LINC00301/miR-1276/HIF1α axis in NSCLC cell proliferation and migration/invasion. Trypan blue staining and colony formation assays demonstrated miR-1276 mimic or HIF1A OE treatment suppressed or facilitated the growth of A549 and SPC-A-1 cells, separately (Fig. 10i, j). However, when A549 and SPC-A-1 cells were treated with miR-1276 mimic plus HIF1A OE, the beneficial role of HIF1α in cell proliferation was inversed by miR-1276, and the growth-inhibitory efficiency of miR-1276 was reversed by HIF1A OE (Fig. 10i, j). These results validated that miR-1276 suppressed cell proliferation through directly targeting the 3′-UTR of HIF1A mRNA. Transwell migration/invasion experiments revealed that miR-1276 mimic or HIF1A OE treatment suppressed or facilitated the migration/invasion in A549 and SPC-A-1 cells, respectively (Fig. 10i, j). Nevertheless, when A549 and SPC-A-1 cells were treated with miR-1276 mimic plus HIF1A OE, the advantageous role of HIF1α in cell migration and invasion was inversed by miR-1276, and the growth inhibitory function of miR-1276 was reversed by HIF1A OE (Fig. 10i, j). These results proved that miR-1276 concealed cell migration/invasion by directly targeting the 3′-UTR of HIF1A mRNA. Therefore, LINC00301’s oncogenic efficiency was partially attributed to sponging miR-1276 and then triggering HIF1α in NSCLC.

In addition, we also tried to analyze the efficiency of integrated prognostic biomarkers including FOXC1, LINC00301, EZH2, HIF1A, and miR-1276, and our results indicated that the higher FOXC1 expression subgroup showed worse prognosis than the lower FOXC1 expression subgroup but with no significance (*p* = 0.1156) in the lower LINC00301 expression group (Additional file 1: Fig. S11A), while we found that the higher FOXC1 expression subgroup significantly exhibited a poorer prognosis than the lower FOXC1 expression subgroup in the higher LINC00301 expression group (*p* < 0.0001) (Additional file 1: Fig. S11B). Furthermore, we also demonstrated that the higher EZH2 expression subgroup showed a poorer prognosis than the lower EZH2 expression subgroup but with no significance (*p* = 0.0526) in the lower LINC00301 expression group (Additional file 1: Fig. S11C), while we found that the higher EZH2 expression subgroup significantly exhibited a poorer prognosis than the lower EZH2 expression subgroup in the higher LINC00301 expression group (*p* = 0.0010) (Additional file 1: Fig. S11D). Furthermore, our analysis revealed that there was no difference between the higher HIF1A expression subgroup and the lower HIF1A expression subgroup for prognosis (*p* = 0.4479) in the lower LINC00301 expression group (Additional file 1: Fig. S11E), while we found that the higher HIF1A expression subgroup significantly exhibited a poorer prognosis than the lower HIF1A expression subgroup in the higher LINC00301 expression group (*p* = 0.0121) (Additional file 1: Fig. S11F). Moreover, our data also exposed that there was no difference between the higher miR-1276 expression subgroup and the lower miR-1276 expression subgroup for prognosis (*p* = 0.8366) in the lower LINC00301 expression group (Additional file 1: Fig. S11G), while we found that the higher miR-1276 expression subgroup significantly exhibited a poorer prognosis than the lower miR-1276 expression subgroup in the higher LINC00301 expression group (*p* = 0.0084) (Additional file 1: Fig. S11H). In addition, we also analyzed the integrated prognostic biomarkers among LINC00301, EZH2, and miR-1276, and our data exposed that there was no difference between the higher miR-1276 expression subgroup and the lower miR-1276 expression subgroup for prognosis (*p* = 0.2785) in the lower LINC00301 plus lower EZH2 expression group (Additional file 1: Fig. S11I). And there was no difference between the higher miR-1276 expression subgroup and the lower miR-1276 expression subgroup for prognosis (*p* = 0.2124) in the lower LINC00301 plus higher EZH2 expression group (Additional file 1: Fig. S11J). Interestingly, our data also uncovered that there was no difference between the higher miR-1276 expression subgroup and the lower miR-1276 expression subgroup for prognosis (*p* = 0.6197) in the higher LINC00301 plus lower EZH2 expression group (Additional file 1: Fig. S11K). And there was no difference between the higher miR-1276 expression subgroup and the lower miR-1276 expression subgroup for prognosis (*p* = 0.1448) in the higher LINC00301 plus higher EZH2 expression group (Additional file 1: Fig. S11L). To classify whether there is a crosstalk between FOXC1/LINC00301/EZH2/EAF2/pVHL/HIF1α and FOXC1/LINC00301/miR-1276/HIF1α pathways, we silenced EZH2, EAF2, and VHL in A549 and SPC-A-1 cells and examined whether miR-1276 expression was changed in comparison to the negative control group. And results indicated that silencing EZH2, EAF2, and VHL had no significant effect on miR-1276 expression in A549 and SPC-A-1 cells, which indicated that FOXC1/LINC00301/EZH2/EAF2/pVHL/HIF1α and FOXC1/LINC00301/miR-1276/HIF1α pathways are at least relatively independent (Additional file 1: Figs. S12A-F). In addition, to validate whether there is a crosstalk between FOXC1/LINC00301/EZH2/EAF2/pVHL/HIF1α and FOXC1/LINC00301/miR-1276/HIF1α pathways in vivo, we constructed a BALB/c nude mice xenograft model with A549 and SPC-A-1 cells. And our data showed that the tumor volume in nude mice treated with LINC00301 OE was markedly increased than that of in CTL group (Additional file 1: Figs. S13A–B), while LINC00301 OE plus EZH2 KD significantly repressed tumor growth derived in nude mice when compared with LINC00301 OE group (Additional file 1: Figs. S13A–B). By contrast, LINC00301 OE plus miR-1276 treatment showed no difference in tumor growth derived in nude mice when compared with LINC00301 OE group (Additional file 1: Figs. S13A–B).

## Discussion

LncRNAs are a kind of > 200 nt-long RNA molecules that are located in the nucleus or cytoplasm and unable to encode proteins. With the vigorous advancement of high throughput technology, a growing amount of lncRNAs are being discovered. LncRNAs have been reported to involve in pathological and physiological processes in various human cancers [19, 27, 40, 46, 47]. The biological function of lncRNAs participates in many processes, including participating in X chromosome inactivation, serving as the skeleton of a nuclear substructure, moderating mRNA degradation, and regulating chromatin remodeling [16, 26, 48].

LncRNAs play essential roles in tumor progression. Recent researches indicated that CAMP/creb-regulated LINC00473 was highly expressed in LKB1-inactivated lung cancer and promoted lung cancer growth [16]. UCA1 endorses the NSCLC cell proliferation and metastasis by functioning as ceRNA to alleviate the targeted inhibition role of miR-193a-3p against its target gene *ERBB4* [49]. LINK-A promotes HIF1α phosphorylation at tyrosine 565 and tryptophan 797 loci by regulating BRK and LRRK2; this process leads to the activation of HIF1α signals under normoxia conditions in triple-negative breast cancer (TNBC), resulting in glycolysis restructuring and breast cancer tumorigenesis [26]. AGAP2-AS1 mediates H3K27me3 at LATS2 and *KLF2* gene promoters by specifically combining with LSD1 and EZH2 to inhibit LATS2 and KLF2 expression levels and promote cell proliferation and migration/invasion but suppress cell apoptosis in NSCLC [50]. To date, the role and mechanism of LINC00301 in NSCLC have not been reported yet. In our current study, we discovered that LINC00301 level is considerably upregulated in NSCLC tissues and cell lines relative to their matched normal lung tissues or bronchial epithelial cells. High LINC00301 expression indicates poor OS in patients with NSCLC. We also discovered that LINC00301 facilitates NSCLC cell proliferation and migration/invasion, represses cell cycle arrest and apoptosis, and accelerates tumor growth in vitro and in vivo. LncRNAs play crucial functions in cancer immunology. It has been reported that lncRNA UCA1 stimulates cell proliferation, migration, immune escape, and apoptosis inhibition in gastric cancer (GC) by acting as a ceRNA for antitumor miRNAs [51]. Hu et al. reported that LINK-A is an oncogenic lncRNA specific in TNBC, and its locked nucleic acid (LNA) treatment considerably improves CD8^+^/CD3^+^ T cell infiltration and cytotoxicity while minimally affects the expression of PD-L1 in TNBC [29]. Moreover, PVT1 controls the immunosuppressive activity of granulocytic MDSC (G-MDSC) in tumor-bearing mice [30]. Furthermore, NKILA endorses tumor immune evasion via inducing T cells to activation-mediated cell death [31]. In the present study, LINC00301 can significantly accumulate Treg and repress CD8^+^ T cell infiltration in the microenvironment of tumors isolated from nude mice burdened with LA-4 and KLN-205 mouse NSCLC cell lines through targeting TGF-β1. These findings indicate that high LINC00301 expression act as a key player in the pathogenesis of NSCLC. In addition, we investigated the mechanism of high LINC00301 expression in NSCLC and discovered that its transcription factor FOXC1, not methylation or deacetylation, regulates LINC00301 expression in NSCLC.

Different cellular localizations may lead to various mechanisms by which lncRNAs exert its function. In general, lncRNAs are participated in the supervision of cancer cell phenotypes through moderating targeted gene expression via various mechanisms, such as genomic imprinting, chromatin modification, RNA decay, and sponging miRNA. FISH experiments showed that LINC00301 is primarily located in the nucleus but is also distributed in the cytoplasm of A549 and SPC-A-1 cells. This finding suggests that LINC00301 could be an essential regulatory player in the nucleus and cytoplasm of A549 and SPC-A-1 cells. LncRNAs could bind with EZH2 in the nucleus and then exhibit oncogenic roles [20, 22]. However, no specific binding sites between LINC00301 and EZH2 protein have been identified yet. In our present study, in vitro RNA pull-down, RIP, RNA EMSA, dot blot, and domain deletion assays demonstrated that LINC00301 (83–123 nt) directly binds with EZH2 (612–727 aa). EZH2 is a key content of the PRC2 complex and mediates H3K27me2 or H3K27me3 in the handles of promoter districts in genes [52]. Herein, we found that LINC00301 directly binds with EZH2 and facilitates H3K27me2/H3K27me3 at the *EAF2* promoter to suppress its transcription. EAF2 represses HIF1α transcriptional activity by interrupting its interaction with the coactivator CBP/p300 [43]. In the present study, LINC00301 inhibited EAF2 expression and then impeded pVHL expression to upregulate HIF1α expression. Further mechanism analysis demonstrated that LINC00301 could also function as ceRNA in the cytoplasm for miR-1276 to alleviate miR-1276’s inhibitory role against HIF1α in the cytoplasm of A549 and SPC-A-1 cells. Hence, LINC00301 facilitates HIF1α expression in the nucleus and cytoplasm and then plays its oncogenic role in NSCLC.

## Conclusions

**Fig. 11:**
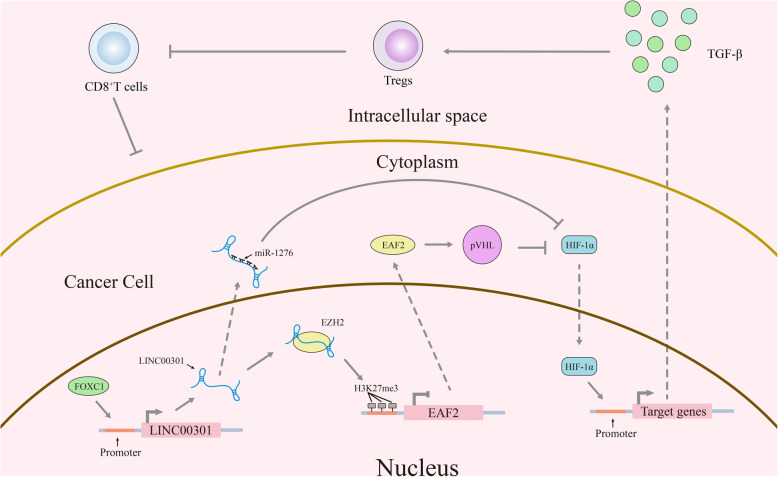
Overview of the involvement of LINC00301 in NSCLC. Schematic representation of our proposed model of FOXC1 upregulates LINC00301 expression, the latter partially mediates oncogenic effects through its epigenetic silencing of EAF2 expression by directly binding with EZH2 to mediate HIF1α activation in the nucleus and by acting as ceRNA for miR-1276 to increase HIF1α expression in the cytoplasm in NSCLC

In summary, our present study established that LINC00301 is upregulated in both NSCLC tumorous tissues and cell lines and is correlated with worse prognosis in NSCLC patients. LINC00301 facilitates cell proliferation and migration/invasion, suppresses cell cycle arrest and apoptosis in vitro, and accelerates tumorigenesis and Treg while represses CD8 T cell infiltration in vivo. Furthermore, we found that it is transcription factor FOXC1, not methylation nor deacetylation, which regulates LINC00301 expression in NSCLC. LINC00301 (83–123 nt) partially mediates oncogenic effects through its epigenetic silence of EAF2 expression through directly interacting with EZH2 (612–727 aa, a part of PRC2) to mediate HIF1α activation in the nucleus and by deeding as a ceRNA for miR-1276 to increase HIF1α expression in the cytoplasm (Fig. 11). Our findings illuminate a potential mechanism that underlies the oncogenic effect of LINC00301 on NSCLC and reveal that LINC00301 may be a valuable biomarker and probable therapeutic target in NSCLC.

## Supplementary information

**Additional file 1.** Supplementary Tables S1-S5 and Figs. S1-S13.

## Data Availability

All data generated or analyzed during this study are included in this published article and its supplementary information files. The relationship between LINC00301, EAF2, and HIF1A expression and prognosis in 2437 lung tumors was analyzed by Kaplan-Meier plotter (http://kmplot.com/analysis/index.php?p=service&cancer=lung) [24]. DNA methylation analysis of lung adenocarcinoma (LUAD) and squamous cell carcinoma (LUSC) samples was conducted using the SMART App (http://www.bioinfo-zs.com/smartapp/) [25] that is based on TCGA Pan-Cancer cohort of UCSC Xena public data hubs (https://xenabrowser.net).

## Abbreviations

LINC00301: Long intergenic non-coding RNA 00301
EZH2: Enhancer of zeste homolog 2
PRC2: Polycomb repressive complex 2
NSCLC: Non-small cell lung cancer
OS: Overall survival
PFS: Progression-free survival
PPS: Post-progression survival
EAF2: ELL protein-associated factor 2
ceRNA: Competing endogenous RNA
Treg: Regulatory T cells
HIF1α: Hypoxia-induced factor 1α
TNBC: Triple-negative breast cancer
TME: Tumor microenvironment
pVHL: von Hippel–Lindau protein
EMT: Epithelial-mesenchymal transition
KD: Knockdown
OE: Overexpression
ICI: Immune checkpoint inhibitors
TAMs: Tumor-associated macrophages
MDSCs: Myeloid-derived suppressor cells
TGF-β1: Transforming growth factor-beta 1
siRNA: Small interfering RNA
DNMT1: DNA (cytosine-5)-methyltransferase 1
5-AZA: 5-Azacytidine
TSA: Trichostatin A
TF: Transcription factor
FISH: Fluorescence in situ hybridization
ChIP: Chromatin immunoprecipitations
RIP: RNA immunoprecipitation
aa: Amino acid
EMSA: Electrophoresis mobility shift assay
GST: Glutathione S-transferase
