# Correction to: FOXC1-mediated LINC00301 facilitates tumor progression and triggers an immune-suppressing microenvironment in non-small cell lung cancer by regulating the HIF1α pathway

**DOI:** 10.1186/s13073-021-00834-w

**Published:** 2021-02-11

**Authors:** Cheng-Cao Sun, Wei Zhu, Shu-Jun Li, Wei Hu, Jian Zhang, Yue Zhuo, Han Zhang, Juan Wang, Yu Zhang, Shao-Xin Huang, Qi-Qiang He, De-Jia Li

**Affiliations:** 1 Department of Preventive Medicine, School of Health Sciences, Wuhan University, No.115 Donghu Road, Wuchang District, Wuhan, 430071 Hubei People’s Republic of China; 2 Department of Molecular and Cellular Oncology, The University of Texas MD Anderson Cancer Center, Houston, TX 77030 USA; 3 Wuhan Hospital for the Prevention and Treatment of Occupational Diseases, Wuhan, 430022 Hubei People’s Republic of China; 4 School of Basic Medicine, Jiujiang University, Jiujiang, 332005 Jiangxi People’s Republic of China

**Correction to: Genome Med 12, 77 (2020)**

**https://doi.org/10.1186/s13073-020-00773-y**

It was highlighted that the original article [1] contained an error in Fig. 2i. Due to the error, during the assembly of Fig. 2i, the cell image at the group plenti-CMV-LINC00301 of A549 invasion part was wrong represented. There was also an inaccurate description for Fig. 4. Due to the error, it was demonstrated that “LINC00301 facilitates regulatory T cells infiltration in tumors isolated from NSCLC cell lines planted C57BL/6 J mice”, actually, it should be “LINC00301 facilitates regulatory T cells infiltration in tumors isolated from NSCLC cell lines planted A/He or DBA/2 mice, respectively”. All related incorrect description for “C57BL/6 J mice” should be “A/He or DBA/2 mice”. This Correction article contains the incorrect and correct Fig. 2 and Fig. 4 and the correct data. The original article has been updated.

**Correction for Fig. 2i and a wrong description for Fig. 4**

1. **Correction for Fig. 2i**

Incorrect Fig. 2

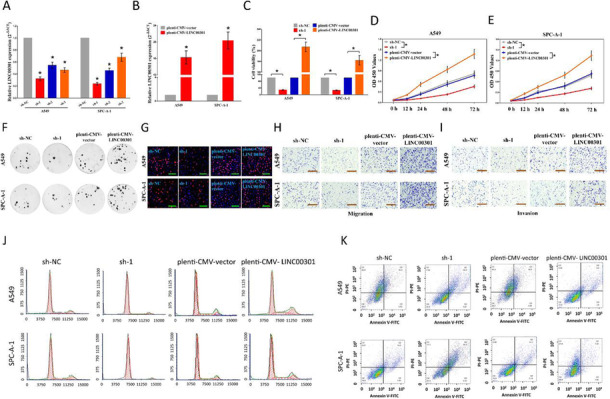



Correct Fig. 2

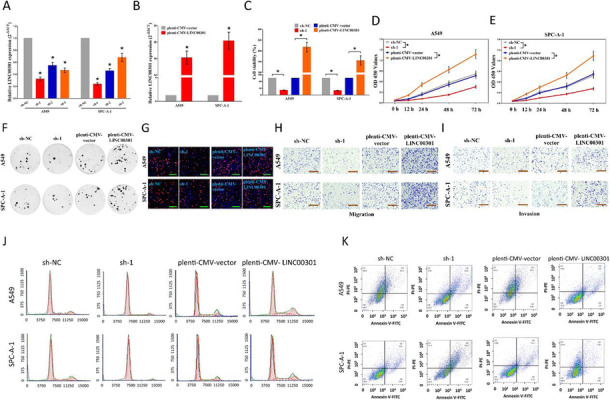



Incorrect data of the group plenti-CMV-LINC00301 of A549 invasion part in Fig. 2i

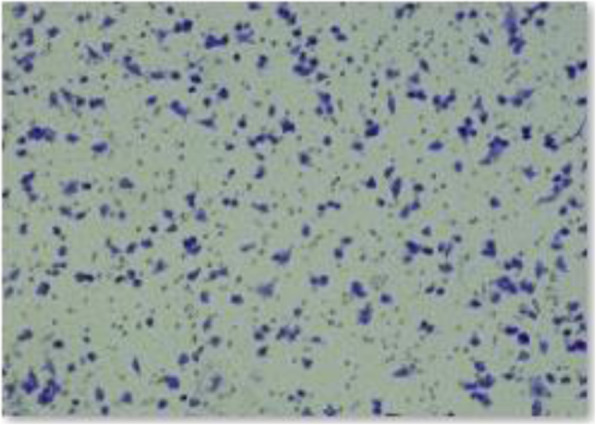



Correct data of the group plenti-CMV-LINC00301 of A549 invasion part in Fig. 2i

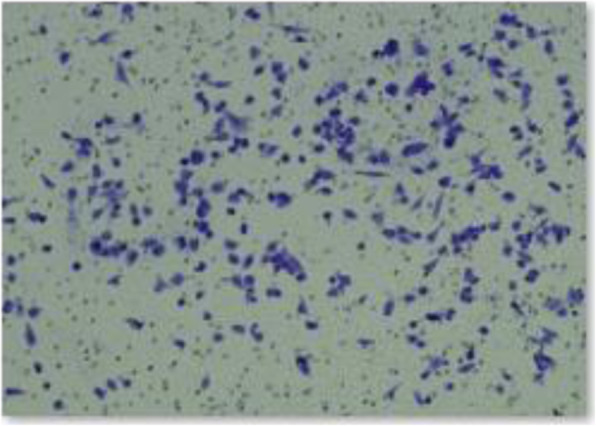



**Fig. 2** LINC00301’s effect on NSCLC cell proliferation, migration and invasion, cell cycle, and cell apoptosis in vitro. **a**, **b** The efficiency of LINC00301 overexpressed and knockout vector transfection. **c**–**e** Trypan blue staining was used to test LINC00301 on NSCLC cell vitality. And CCK8 assay indicated LINC00301 on NSCLC cell proliferation. **f** Colony formation assay (seeded at 24-well plate) indicated LINC00301 on NSCLC cell proliferation. **g** BrdU staining assay indicated LINC00301 on NSCLC cell proliferation. Bar = 100 μm. **h**, **i** Representative images of transwell migration/invasion assay for LINC00301’s role in NSCLC cell migration and invasion ability. **j**, **k** Representative images for flow cytometry analysis of A549 and SPC-A-1 cells after transfection. Cell cycle analysis discovered that LINC00301 has affected the A549 and SPC-A-1 cells proliferation (**j**), and cell apoptosis analysis showed that LINC00301 has affected the cell apoptosis of A549 and SPC-A-1 cells (**k**). *p < 0.05, means ± SD was shown. Statistical analysis was performed by Student’s t-test analysis.

2 **Correction for a wrong description of Fig. 4**

**Moreover**, we also make a wrong description for Fig. 4, in the original version, we demonstrated that “LA-4 and KLN 205 cells were transfected with sh-1/sh-NC, pLenti-CMV-LINC00301/pLenti-CMV-vector, and then transplanted to C57BL/6 J mice”, actually, it should be “LA-4 and KLN 205 cells were transfected with sh-1/sh-NC, pLenti-CMV-LINC00301/pLenti-CMV-vector, and then transplanted to A/He or DBA/2 mice, respectively” . Hence, we need correct those wrong descriptions. Here are detailed sites that needed revision.

1. **In the method parts**
  “CyTOF run and sample normalization
  Sample run and normalization
  LA-4 and KLN 205 cells were transfected with sh-1/sh-NC, pLenti-CMV-LINC00301/pLenti-CMV-vector, and then transplanted to C57BL/6 J mice.”
  Should be replaced by
  “CyTOF run and sample normalization
  Sample run and normalization
  LA-4 and KLN 205 cells were transfected with sh-1/sh-NC, pLenti-CMV-LINC00301/pLenti-CMV-vector, and then transplanted to A/He or DBA/2 mice, respectively.”
2. **In the result parts**

- “Hence, we examined the roles of LINC00301 OE and KD of LA-4 and KLN 205 cells on immune cell infiltration in tumors isolated from C57BL/6 J mice.”
  Should be replaced by
  “Hence, we examined the roles of LINC00301 OE and KD of LA-4 and KLN 205 cells on immune cell infiltration in tumors isolated from A/He or DBA/2 mice, respectively.”
- “The results demonstrated that LINC00301 KD repressed Treg but facilitated CD8+ T cell infiltration while LINC00301 OE accumulated Treg but suppressed CD8+ T cell infiltration in LA-4/KLN-205 burdened tumors in C57BL/6 J mice (Fig. 4c–f), suggesting LINC00301 exerts an immune-suppressive role in NSCLC by recruiting Treg cells.”
  Should be replaced by
  “The results demonstrated that LINC00301 KD repressed Treg but facilitated CD8+ T cell infiltration while LINC00301 OE accumulated Treg but suppressed CD8+ T cell infiltration in LA-4/KLN-205 burdened tumors in A/He- /DBA/2- mice, respectively (Fig. 4c–f), suggesting LINC00301 exerts an immune-suppressive role in NSCLC by recruiting Treg cells.”

3. **Figure 4 legends part**
  “LINC00301 facilitates regulatory T cells infiltration in tumors isolated from NSCLC cell lines planted C57BL/6 J mice. a, b Representing images for CyTOF analysis of tumors by PhenoGraph analysis, identifying 22 clusters, colored by cluster identification numbers, and plotted by tSNE1 and tSNE2, clarified various clusters with obvious alterations between groups. c Flow cytometry gating method used in the analysis of CD3+/CD4+/CD25+ regulatory T cells infiltration was presented. Shown is a pLenti-CMV-vector tumor cell preparation. Following a preliminary assessment of a lymphocyte gate according to forward versus side scatter, feasible cells (FITC positive) were identified as CD3+ cells. The resulting CD3+ population was following gated according to positivity for CD4 +/CD25+. The frequencies of cells in these populations were normalized by background values and then submitted and evaluated directly. d Flow cytometry measurement of percentage of CD3+/CD4+/CD25+ Treg cells. e Representative images of CD4 and CD25 of tumors isolated from sh-NC, sh-1, pLenti-CMV-vector, or pLenti-CMV-LINC00301 treated LA-4 and KLN 205 cells planting in C57BL/6 J mice (*n* = 5 animals for each group). f Representative images of CD8 of tumors isolated from sh-NC, sh-1, pLenti-CMV-vector, or pLenti-CMV-LINC00301-treated LA-4 and KLN 205 cells planting in C57BL/6 J mice (*n* = 5 animals for each group). Scale bar = 50 μm, p values were established by unpaired two-tailed Student’s t-test”
  Should be replaced by
  “LINC00301 facilitates regulatory T cells infiltration in tumors isolated from mouse NSCLC cell lines planted A/He or DBA/2 mice, respectively. a, b Representing images for CyTOF analysis of tumors by PhenoGraph analysis, identifying 22 clusters, colored by cluster identification numbers, and plotted by tSNE1 and tSNE2, clarified various clusters with obvious alterations between groups. c Flow cytometry gating method used in the analysis of CD3+/CD4+/CD25+ regulatory T cells infiltration was presented. Shown is a pLenti-CMV-vector tumor cell preparation. Following a preliminary assessment of a lymphocyte gate according to forward versus side scatter, feasible cells (FITC positive) were identified as CD3+ cells. The resulting CD3+ population was following gated according to positivity for CD4 +/CD25+. The frequencies of cells in these populations were normalized by background values and then submitted and evaluated directly. d Flow cytometry measurement of percentage of CD3+/CD4+/CD25+ Treg cells. e Representative images of CD4 and CD25 of tumors isolated from sh-NC, sh-1, pLenti-CMV-vector, or pLenti-CMV-LINC00301 treated LA-4 and KLN 205 cells planting in A/He or DBA/2 mice, respectively (*n* = 5 animals for each group). f Representative images of CD8 of tumors isolated from sh-NC, sh-1, pLenti-CMV-vector, or pLenti-CMV-LINC00301-treated LA-4 and KLN 205 cells planting in A/He or DBA/2 mice, respectively (*n* = 5 animals for each group). Scale bar = 50 μm, p values were established by unpaired two-tailed Student’s t-test”
