# Correction: FOXC1-mediated LINC00301 facilitates tumor progression and triggers an immune-suppressing microenvironment in non-small cell lung cancer by regulating the HIF1α pathway

**DOI:** 10.1186/s13073-023-01172-9

**Published:** 2023-04-12

**Authors:** Cheng-Cao Sun, Wei Zhu, Shu-Jun Li, Wei Hu, Jian Zhang, Yue Zhuo, Han Zhang, Juan Wang, Yu Zhang, Shao-Xin Huang, Qi-Qiang He, De-Jia Li

**Affiliations:** 1 Department of Preventive Medicine, School of Health Sciences, Wuhan University, No.115 Donghu Road, Wuchang District, Wuhan, 430071 Hubei People’s Republic of China; 2 Department of Molecular and Cellular Oncology, The University of Texas MD Anderson Cancer Center, Houston, TX 77030 USA; 3 Wuhan Hospital for the Prevention and Treatment of Occupational Diseases, Wuhan, 430022 Hubei People’s Republic of China; 4 School of Basic Medicine, Jiujiang University, Jiujiang, 332005 Jiangxi People’s Republic of China

**Correction: Genome Med 12, 77 (2020)**

**https://doi.org/10.1186/s13073-020-00773-y**

The original publication of this article [1] contained the following errors:

1. Figures 2 & 9 & S9 were mislabeled.
2. There was a wrong description for Fig. 9E. Due to the error, the Y axis in left and right panel of Fig. 9E should be “Cell viabilities”, but it was written as “Cell viability”. The statistical analysis for Fig. S9B-E was added and presented in Fig. S9F.

The old and new figures are shown in this correction article as Figs. 1(Original/incorrect Fig. 2), 2 (Correct Fig. 2), 3 (Original/incorrect Fig. 9), 4 (Correct Fig. 9), 5 (Original/incorrect Fig. S9), and 6 (Correct Fig. S9). The original article has been updated. The authors declare that this does not affect the results and conclusion of the publication.

**Fig. 1:**
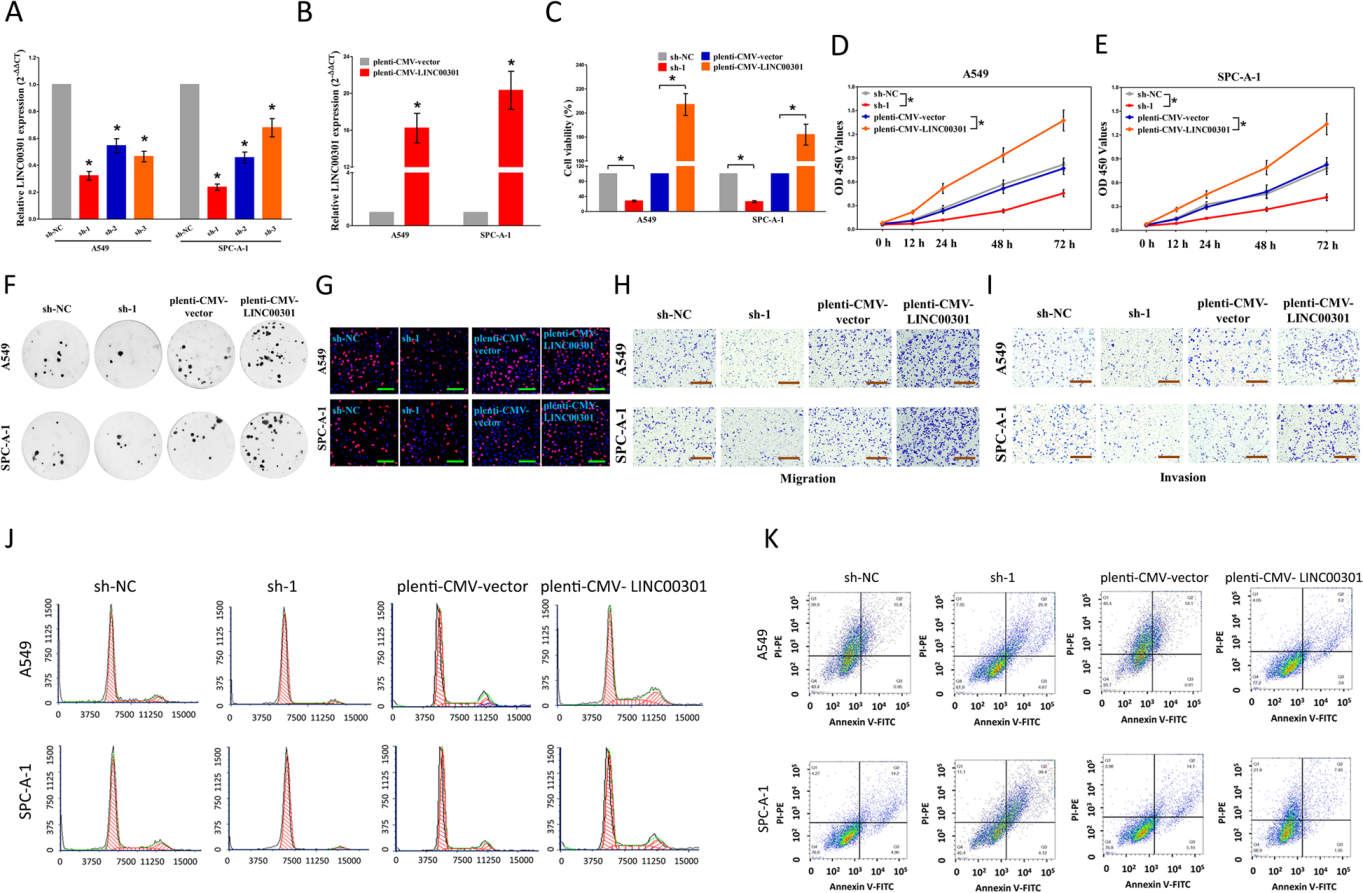
Original (incorrect) version of figure 2

**Fig. 2:**
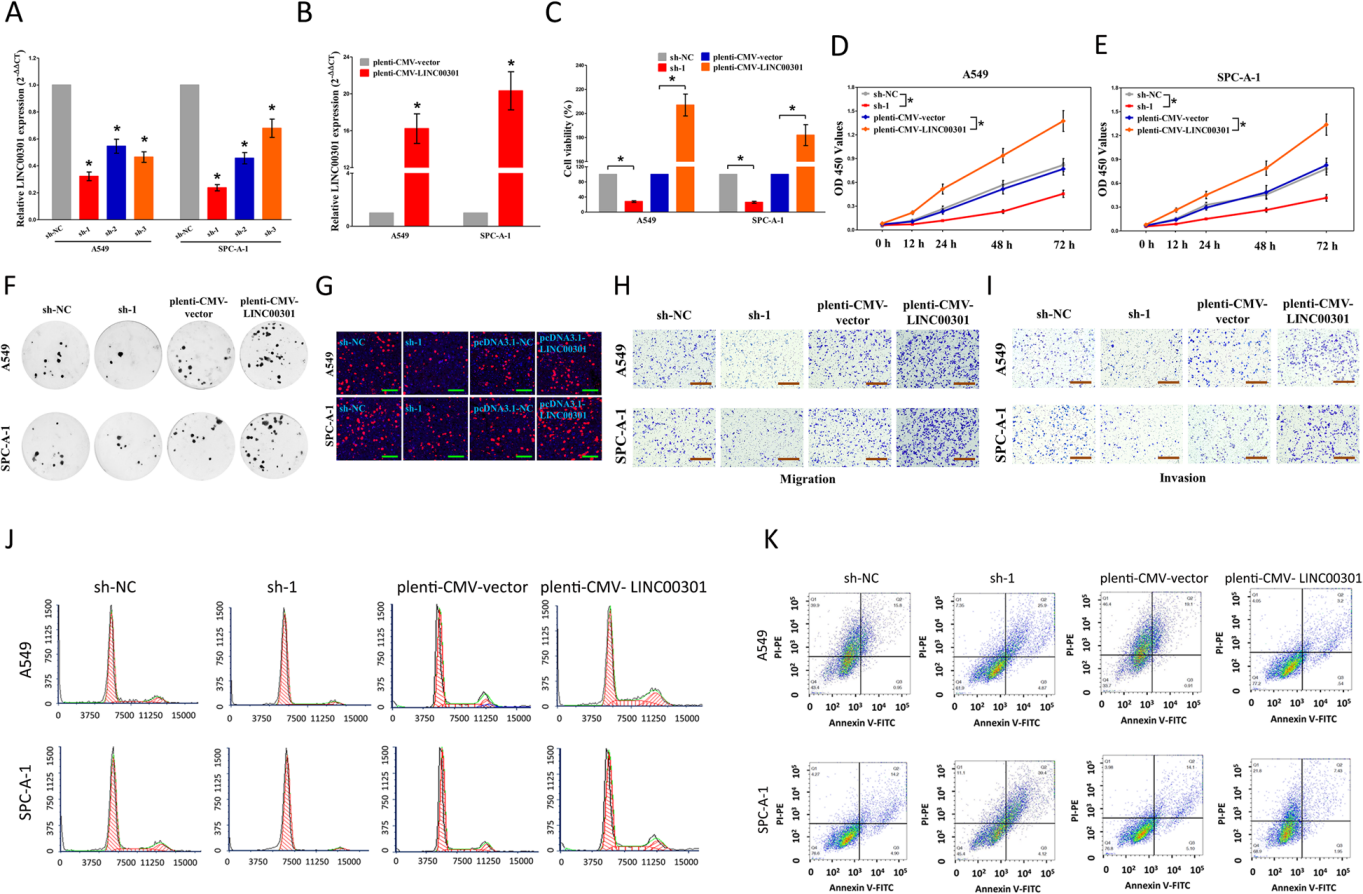
Correct version of figure 2

**Fig. 3:**
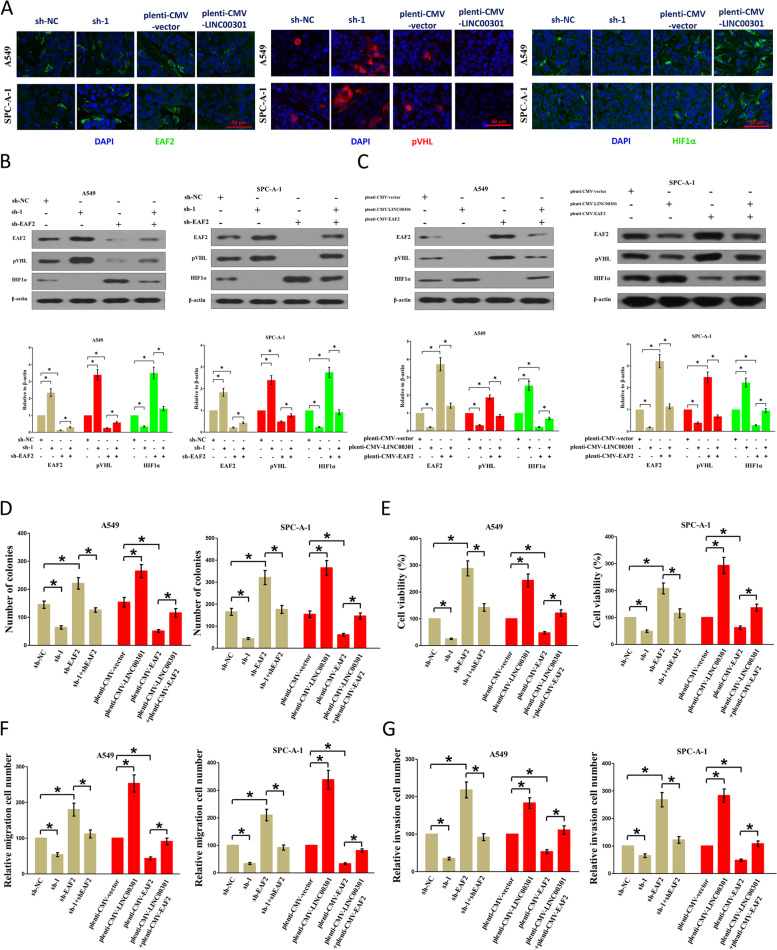
Original (incorrect) version of figure 9

**Fig. 4:**
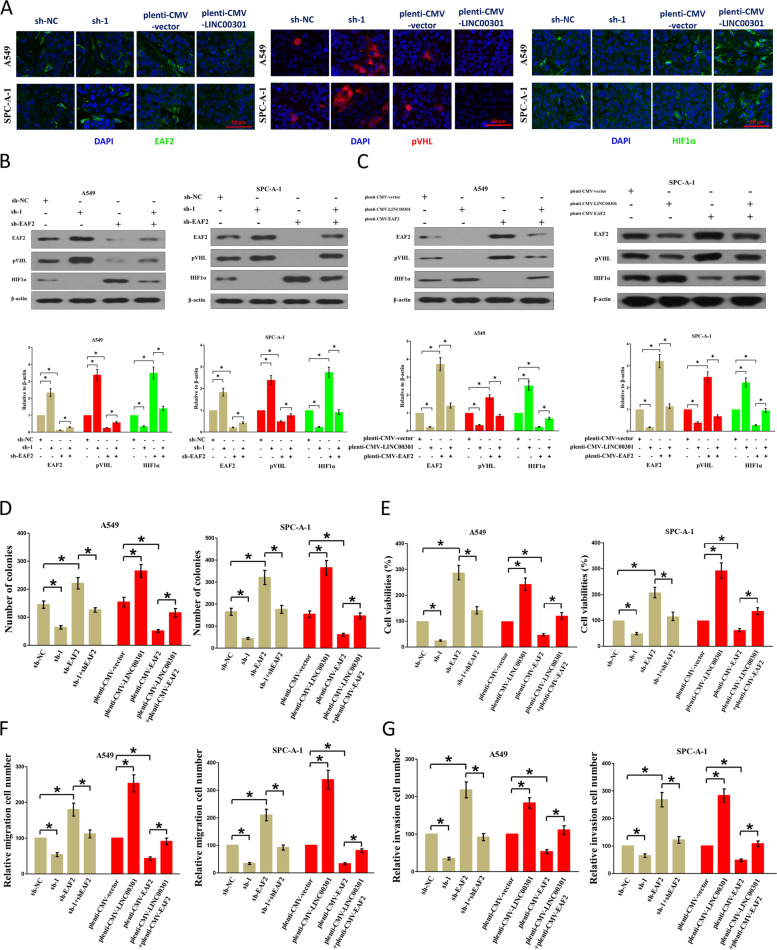
Correct version of figure 9

**Fig. 5:**
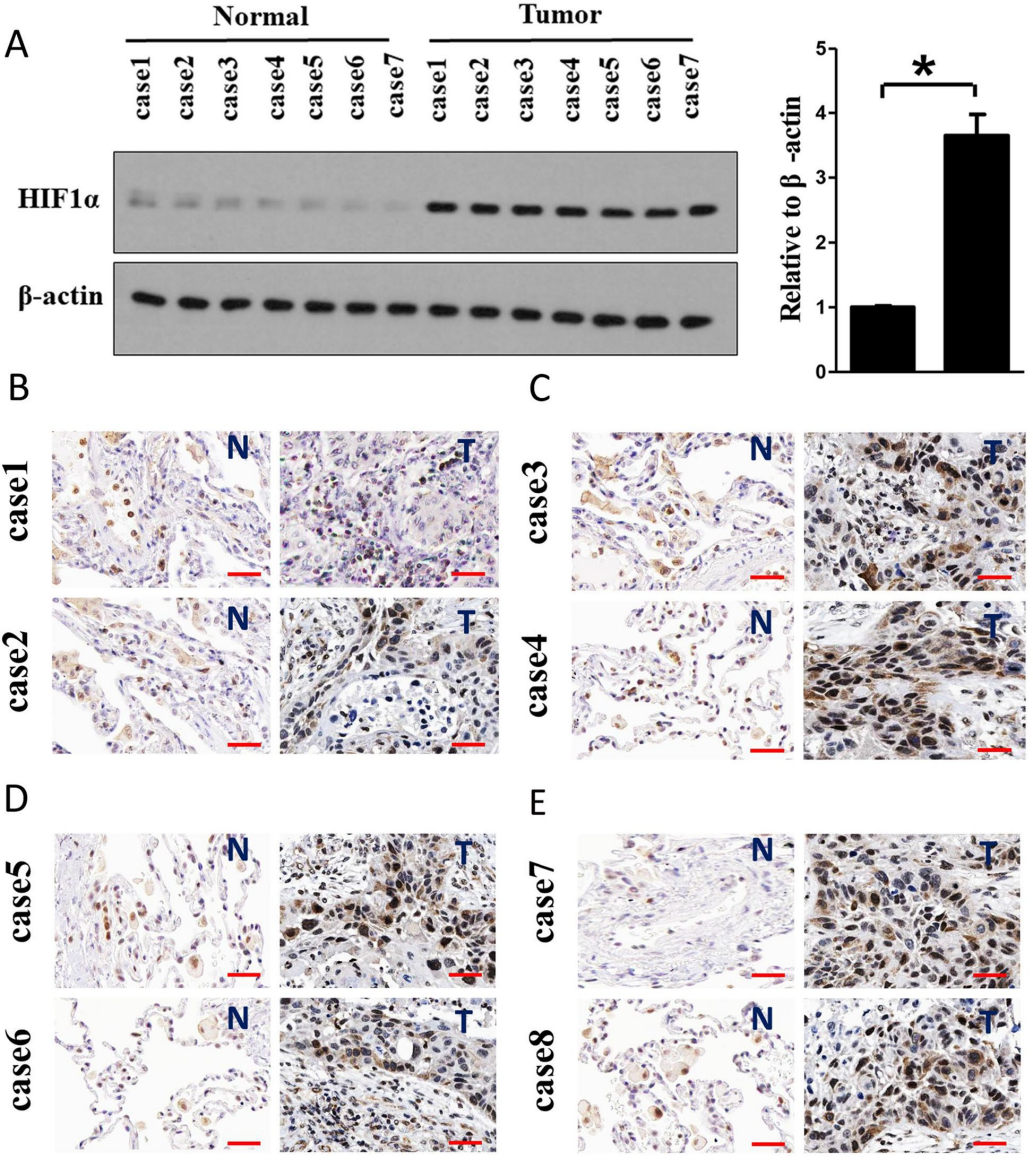
Original (incorrect) version of figure S9

**Fig. 6:**
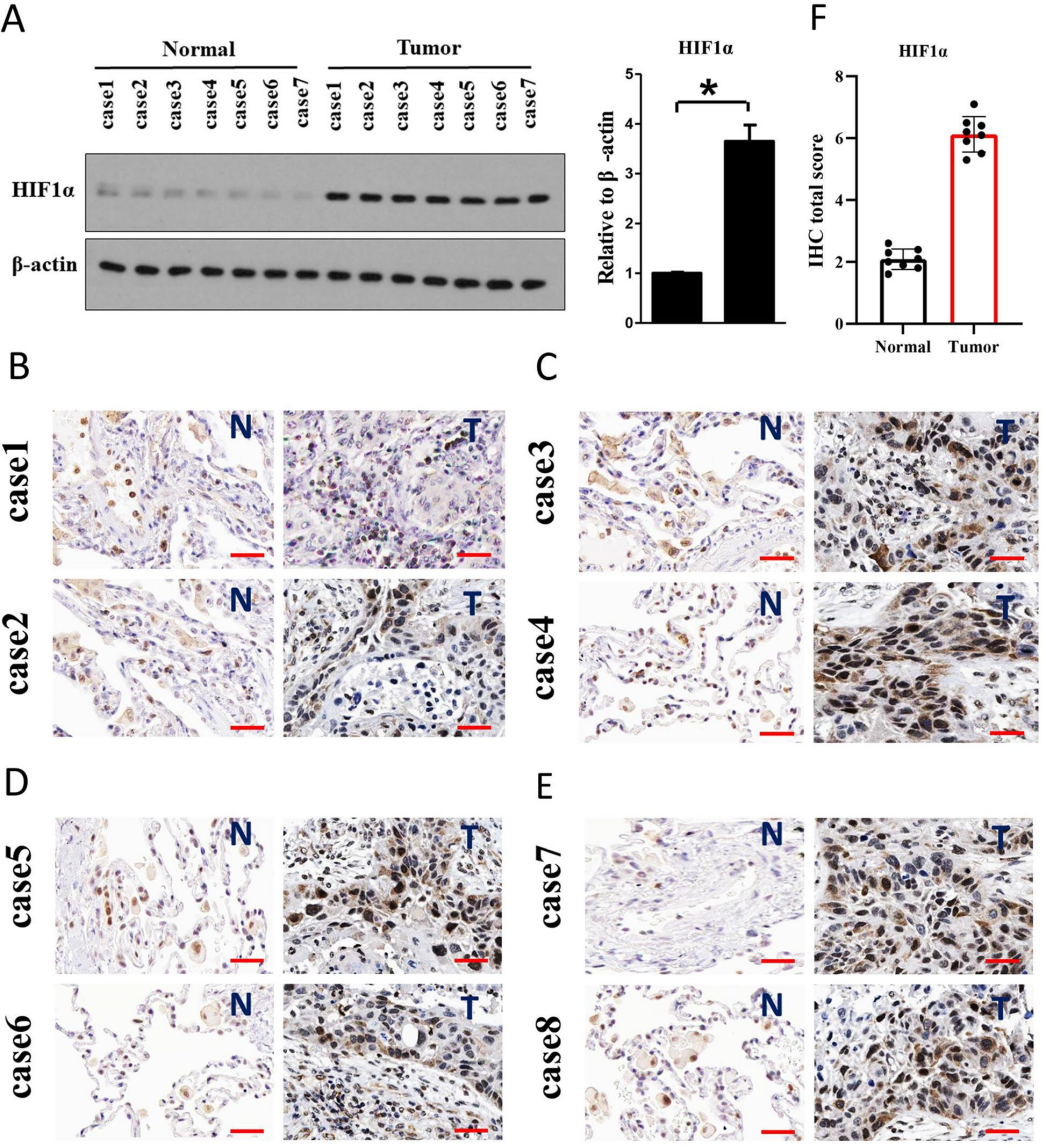
Correct version of figure S9
