# Supplementary material for: FOXC1-mediated LINC00301 facilitates tumor progression and triggers an immune-suppressing microenvironment in non-small cell lung cancer by regulating the HIF1α pathway
Source: Genome Med. 2020 Sep 2;12:77. doi: 10.1186/s13073-020-00773-y (PMC7466809; doi:10.1186/s13073-020-00773-y)
Supplement: Supplementary file 1 — Additional file 1. Supplementary Tables S1-S5 and Figs. S1-S13. [file 13073_2020_773_MOESM1_ESM.docx]

**FOXC1-mediated LINC00301 facilitates tumor progression and triggers immune-suppressing microenvironments in non-small cell lung cancer by regulating the HIF1α pathway**

Cheng-Cao Sun ^1,2, *,#^, Wei Zhu ^1,#^, Shu-Jun Li ^3,#^, Wei Hu ^1^, Jian Zhang ^1^, Yue Zhuo ^1^, Han Zhang ^1^, Juan Wang ^1^, Yu Zhang ^1^, Shao-Xin Huang ^4^, Qi-Qiang He ^1,*^, and De-Jia Li ^1,*^

1. Department of Preventive Medicine, School of Public Health, Wuhan University, Wuhan, Hubei 430071, P. R. China.
2. Department of Molecular and Cellular Oncology, The University of Texas MD Anderson Cancer Center, Houston, TX 77030, USA.
3. Wuhan Hospital for the Prevention and Treatment of Occupational Diseases, Wuhan, Hubei 430022, P. R. China.
4. School of Basic Medicine, Jiujiang University, Jiujiang, Jiangxi 332005, P. R. China

* Corresponding Author

# Cheng-Cao Sun, Wei Zhu, and Shu-Jun Li contributed equally to this work.

**Supplementary Tables S1-S5 and Figures S1-S13.**

| **Table S1. Oligos used in this study** | | |
| --- | --- | --- |
| **Oligos** | **note** | **Sequences** |
| H 301-sh324-F | sh-LINC00301#1-F | CCGGCCCAGTAACAGAGGGAGCAAAGCAACTCG  AGTTGCTTTGCTCCCTCTGTTACTGGGTTTTTG |
| H 301-sh324-R | sh-LINC00301#1-R | AATTCAAAAACCCAGTAACAGAGGGAGCAAAGC  AACTCGAGTTGCTTTGCTCCCTCTGTTACTGGG |
| H 301-sh226-F | sh-LINC00301#2-F | CCGGTAAACAGTTTCGGCCTTGCAATAATCTCGAG  ATTATTGCAAGGCCGAAACTGTTTATTTTTG |
| H 301-sh226-R | sh-LINC00301#2-R | AATTCAAAAATAAACAGTTTCGGCCTTGCAATAATC  TCGAG ATTATTGCAAGGCCGAAACTGTTTA |
| H 301-sh117-F | sh-LINC00301#3-F | CCGGCATCCTTTCTAATGTCTACACCATACTCGAGTA  TGGTGTAGACATTAGAAAGGATGTTTTTG |
| H 301-sh117-R | sh-LINC00301#3-R | AATTCAAAAACATCCTTTCTAATGTCTACACCATACTC  GAGTATGGTGTAGACATTAGAAAGGATG |
| H 301-qPCR-F |  | GGCCACATGTACCCAGTAACA |
| H 301-qPCR-R |  | TTTGCTGAGCTCCGGGATAG |
| H GAPDH-qPCR-F |  | CTCTGCTCCTCCTGTTCGAC |
| H GAPDH-qPCR-R |  | ACCAAATCCGTTGACTCCGA |
| H 301-FL-pENTR221 F |  | CACCAGGGTGAGGGGCGCCACCGA |
| H 301-FL-pENTR221 R |  | TGTGATCAAATCGCATGCTTTATT |
| H EAF2-FL-pENTR221 F |  | CACCATGAATAGCGCAGCGGGATT |
| H EAF2-FL-pENTR221 R |  | TCAGTCATCACTGTCACTTCCTGA |
| H HIF1A-FL-pENTR221 F |  | CACCATGGAGGGCGCCGGCGGCGC |
| H HIF1A-FL-pENTR221 R |  | TCAGTTAACTTGATCCAAAGCTCT |
| H 301-FL-pGEM3Z-KpnI F |  | GGTACCAGGGTGAGGGGCGCCACCGA |
| H 301-FL-pGEM3Z-NheI R |  | GCTAGCTGTGATCAAATCGCATGCTTTA |
| Antisense-H301-oligo 1 |  | GGGAGCCCGGTGGGCGCTTCCTCGGTGGCGCCCCTCACCCT |
| Antisense-H301-oligo 2 |  | ATCTGGCAGTCAACTTCCTCAATTTGGGGCCTGGCAGCCCC |
| Antisense-H301-oligo 3 |  | AAGGATGAATGGAATCTATCTGCGTGGGCCAACAGAGTGTG |
| Antisense-H301-oligo 4 |  | AGATGATGGCATGTTATCCAAAATATGGTGTAGACATTAGA |
| Antisense-H301-oligo 5 |  | ATCAACCCCAACATTCTGGTTTCTTCTTTCAACACAATGTG |
| Antisense-H301-oligo 6 |  | TTGCAAGGCCGAAACTGTTTAAATTCATCCATATGGCATAC |
| Antisense-H301-oligo 7 |  | AAAGAGTATAATTTCACATGCAATTAGAAGTATACCAATTA |
| Antisense-H301-oligo 8 |  | CTGGGTACATGTGGCCACTGAATAGTTTCATCTTTCGTTAA |
| Antisense-H301-oligo 9 |  | GCAGTTCTTTGAGGATAAGATTTGCTTTGCTCCCTCTGTTA |
| Antisense-H301-oligo 10 |  | AGTTTCTCTATGGAACCACCAGGTAGATCTAACAAGCAGCT |
| Antisense-H301-oligo 11 |  | ATAGTTCTGTTTTAGGCGAGGTAAAGGCAGGTTCTCTGAGC |
| Antisense-H301-oligo 12 |  | CAGTTATAGAGGATGAATGGACTCCTTTGCTGAGCTCCGGG |
| Antisense-H301-oligo 13 |  | AGGATAAATCTTCTCAGCATTTTAACAGAGATATTGTATAG |
| Antisense-H301-oligo 14 |  | ATCCTGAGTTGATGAACCCCATTGCTTTGGTGCCTTCTTTT |
| Antisense-H301-oligo 15 |  | TGCCTTATATCTCGTAACAGGCTTCTGTATTACACTACTGG |
| Antisense-H301-oligo 16 |  | GATTTCCAAGGATTCCGACAGTGAGATTTCCGAGGGCAAAG |
| Antisense-H301-oligo 17 |  | TTCTTTGCAAATTATGAAGAAATAGAAGTTGTGCGTGGTGT |
| Antisense-H301-oligo 18 |  | ATTCACAATTAATCTTTTGTTCTTGACTGGAGTCACACTGG |
| Antisense-H301-oligo 19 |  | TTTTCCATGTGGCTGCTTGAAAAAATTAGAGTTCTTCGCTA |
| Antisense-H301-oligo 20 |  | CTAAGAAAAAAGAGCAATGGAAAGGATTTCATTAGAATGCG |
| Antisense-H301-oligo 21 |  | TGTGATCAAATCGCATGCTTTATTGAAGAA |
| H EZH2-sh558-F |  | CCGGGGATGGTACTTTCATTGAAGACTCGAGTCTT  CAATGAAAGTACCATCCTTTTTG |
| H EZH2-sh558-R |  | AATTCAAAAAGGATGGTACTTTCATTGAAGACTCG  AGTCTTCAATGAAAGTACCATCC |
| H EZH2-sh657-F |  | CCGGGGTGAATGCCCTTGGTCAATACTCGAGTATT  GACCAAGGGCATTCACCTTTTTG |
| H EZH2-sh657-R |  | AATTCAAAAAGGTGAATGCCCTTGGTCAATACTCG  AGTATTGACCAAGGGCATTCACC |
| H EZH2-sh1051-F |  | CCGGGCAACACCCAACACTTATAAGCTCGAGCTTA  TAAGTGTTGGGTGTTGCTTTTTG |
| H EZH2-sh1051-R |  | AATTCAAAAAGCAACACCCAACACTTATAAGCTCG  AGCTTATAAGTGTTGGGTGTTGC |
| H EAF2-sh678-F |  | CCGGGATTGCAAATCCTCTACTTCTCTCGAGAGAA  GTAGAGGATTTGCAATCTTTTTG |
| H EAF2-sh678-R |  | AATTCAAAAAGATTGCAAATCCTCTACTTCTCTCGA  GAGAAGTAGAGGATTTGCAATC |
| H EAF2-sh707-F |  | CCGGGAATTGTGTCTCAGGACATCCCTCGAGGGAT  GTCCTGAGACACAATTCTTTTTG |
| H EAF2-sh707-R |  | AATTCAAAAAGAATTGTGTCTCAGGACATCCCTCGA  GGGATGTCCTGAGACACAATTC |
| H EAF2-sh792-F |  | CCGGGGCCTTCTGATGAATACTTTACTCGAGTAAAG  TATTCATCAGAAGGCCTTTTTG |
| H EAF2-sh792-R |  | AATTCAAAAAGGCCTTCTGATGAATACTTTACTCGAG  TAAAGTATTCATCAGAAGGCC |

| **Table S2. antibodies list used for cy-TOF analysis** | | | | | | | |
| --- | --- | --- | --- | --- | --- | --- | --- |
| TaggedAbs.description | Target | label | IntracellularStaining | clone | specificities | Source | cat |
| CD3 174Yb | CD3 | 174Yb | FALSE | 17A2 | Ms | BioLegend | 100201 |
| CD4(Ms) 115In | CD4(Ms) | 115In | FALSE | RM4-5 | Ms | BioLegend | 100506 |
| CD25(Ms) 150Nd | CD25 | 150Nd | FALSE | 3C7 | Ms | DVS-Fluidigm | 3150002B |
| Foxp3(Ms) 158Gd | Foxp3 | 158Gd | TRUE | FJK-16s | Ms, Rt, Bv, Cn, Po, Fe | DVS-Fluidigm | 3158003A |
| CD11b 143Nd | CD11b | 143Nd | FALSE | M1/70 | Ms, Hu | DVS-Fluidigm | 3143015B |
| CD45(Ms) 89Y | CD45(Ms) | 89Y | FALSE | 30-F11 | Ms | DVS-Fluidigm | 3089005B |
| CX3CR1 176Yb | CX3CR1 | 176Yb | FALSE | SA011F11 SA011F11 SA011F11 | Ms | BioLegend | 149002 |
| CD33(Ms/Hu) 173Yb | CD33 | 173Yb | FALSE | 6C5/2 | Ms, Hu | Abcam | ab11032 |
| F4/80(Ms) 159Tb (MDA) | F4/80 | 159Tb | FALSE | BM8 | Ms | BioLegend | 123102 |
| Ly-6G/C 175Lu | Ly-6G/Ly-6C, Gr-1 | 175Lu | FALSE | RB6-8C5 | Ms | BioLegend | 108402 |
| CD19(Ms) 166Er | CD19 | 166Er | FALSE | 6D5 | Ms | DVS-Fluidigm | 3166015B |
| NK1.1 170Er (MDA) | NK1.1 | 170Er | FALSE | PK136 | Ms | BioLegend | 108702 |
| CD279(Ms) 159Tb | CD279, PD-1 | 159Tb | FALSE | 29F.1A12 | Ms | DVS-Fluidigm | 3159024B |
| CD11c(Ms) 209Bi | CD11c | 209Bi | FALSE | N418 | Ms | DVS-Fluidigm | 3209005B |
| CD44 160Gd | CD44 | 160Gd | FALSE | IM7 | Hu, Ms, Ch, Rh | BioLegend | 103002 |
| CD62L(Ms) 164Dy | CD62L | 164Dy | FALSE | MEL-14 | Ms | DVS-Fluidigm | 3164003B |
| CD8a(Ms) 146Nd (MDA) | CD8a | 146Nd | FALSE | 53-6.7 | Ms | BioLegend | 100702 |
| CD68(Ms) 145Nd | CD68 | 145Nd | TRUE | FA-11 | Ms | BioLegend | 137002 |
| iNOS 141Pr | iNOS | 141Pr | TRUE | SP126 | Hu, Ms | Abcam | ab239990 |
| Arginase-1 166Er (MDA) | Arg-1, Arginase-1 | 166Er | TRUE | O94E6/ARG1 | Hu, Ms | BioLegend | 678802 |
| CD152(Ms) 163Dy | CD152, CTLA-4 | 163Dy | FALSE | 9H10 | Ms | BioLegend | 106202 |
| CD86(Ms) 172Yb | CD86 | 172Yb | FALSE | GL1 | Ms | DVS-Fluidigm | 3172016B |

| **Table S3**. Correlation between LINC00301 expression and clinicopathological parameters of NSCLC patients **(n=120)** | | | | | | | | | |  |
| --- | --- | --- | --- | --- | --- | --- | --- | --- | --- | --- |
| Parameter | | n | | Relative LINC00301 expression | | | | | |  |
|  |  |  |  | Low (60) | | High(60) | | p value[a] | |  |
| Age/years | |  | |  | |  | | 0.5764 | |  |
| ≤ 65 | | 48 | | 22 | | 26 | |  | |  |
| > 65 | | 72 | | 38 | | 34 | |  | |  |
| Gender | |  | |  | |  | | 0.3479 | |  |
| Male | | 74 | | 34 | | 40 | |  | |  |
| Female | | 46 | | 26 | | 20 | |  | |  |
| Differentiation | |  | |  | |  | | 0.8549 | |  |
| Well, moderate | | 64 | | 31 | | 33 | |  | |  |
| Poor | | 56 | | 29 | | 27 | |  | |  |
| Tumor size (maximum diametercm) | |  | |  | |  | | 0.0360[*] | |  |
| ≤ 3cm | | 31 | | 21 | | 10 | |  | |  |
| > 3cm | | 89 | | 39 | | 50 | |  | |  |
| Smoking history | |  | |  | |  | | 0.5361 | |  |
| Smokers | | 88 | | 46 | | 42 | |  | |  |
| Never smokers | | 32 | | 14 | | 18 | |  | |  |
| Lymph node metastasis | |  | |  | |  | | <0.0001[*] | |  |
| Positive | | 78 | | 24 | | 54 | |  | |  |
| Negative | | 42 | | 36 | | 6 | |  | |  |
| TMN stage | |  | |  | |  | | 0.0142 [*] | |  |
| I | | 40 | | 25 | | 15 | |  | |  |
| II/III/IV | | 80 | | 35 | | 55 | |  | |  |
| Histological tumor type | |  | |  | |  | | 0.2105 | |  |
| Squamous cell carcinoma | | 31 | | 12 | | 19 | |  | |  |
| Adenocarcinoma | | 89 | | 48 | | 41 | |  | |  |
| **Table S4.** Influence of LINC00301 expression and clinical characteristics on overall survival in NSCLC patients | | | | | | | | | | |
| Factors | Subset | | Univariate analysis | | | | Multivariate analysis | | | |
|  |  |  | HR (95%CI) | | p value | | HR (95%CI) | | p value | |
| Age(years) | >60/≤60 | | 1.81 (1.01-3.52) | | 0.213 | | 2.17 (0.98-1.63) | | 0.258 | |
| Sex | Male/Female | | 1.76 (0.87-1.58) | | 0.674 | |  | |  | |
| Differentiation | Poor /Well, moderate | | 1.65 (1.19-1.78) | | 0.428 | |  | |  | |
| Tumor size | >3cm/≤3cm | | 2.01 (0.88-2.66) | | 0.533 | |  | |  | |
| Smoking | Yes/No | | 1.35 (0.86-2.15) | | 0.474 | |  | |  | |
| Lymph node metastasis | Positive/ Negative | | 3.24 (1.74-3.42) | | 0.013 | | 3.85 (1.26-5.78) | | 0.006 | |
| TMN stage | (II/III/IV)/I | | 2.16 (1.22-3.76) | | 0.008 | | 2.98 (1.94-4.46) | | 0.003 | |
| LINC00301 | High/Low | | 4.75 (2.12-5.27) | | <0.001 | | 5.51 (3.05-12.15) | | <0.001 | |
| Histological tumor type | Squamous cell carcinoma/ Adenocarcinoma | | 1.24 (0.67-1.82) | | 0.642 | |  | |  | |
| HR, hazard ratio; CI, confidence interval. | | |  | |  | |  | |  | |

**Table S5.** Predicted results (LINC00301) using miRDB (target score≥50)

| Target Rank | Target Score | miRNA Name | Gene Symbol |
| --- | --- | --- | --- |
| 1 | 78 | hsa-miR-4756-3p | submission |
| 2 | 73 | hsa-miR-1276 | submission |
| 3 | 70 | hsa-miR-876-5p | submission |
| 4 | 70 | hsa-miR-3167 | submission |
| 5 | 68 | hsa-miR-1227-5p | submission |
| 6 | 66 | hsa-miR-382-5p | submission |
| 7 | 65 | hsa-miR-8073 | submission |
| 8 | 62 | hsa-miR-6762-3p | submission |


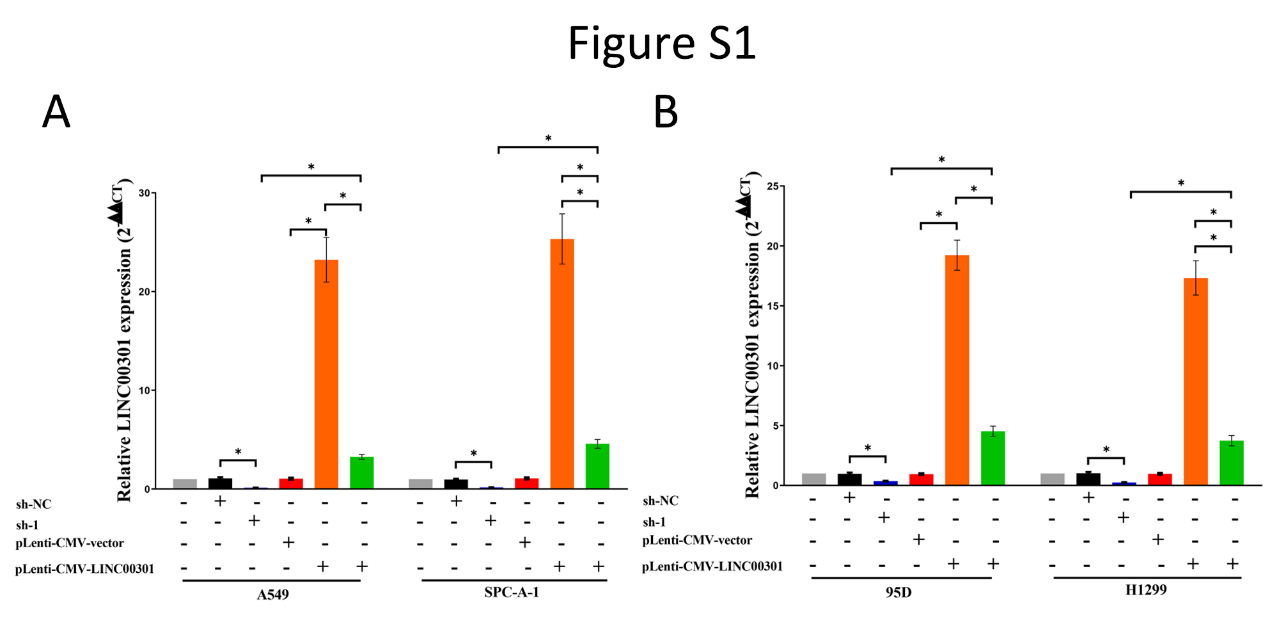


**Fig S1.** Knockdown and rescue experiments for LINC00301 were conducted in four NSCLC cancer cell lines, including A549 and SPC-A-1 **(A)**, 95D and H1299 **(B)**. We treated the cells for 48 hrs with shRNA against LINC00301 and rescued the cells with 24 hrs of plasmid overexpressing LINC00301.


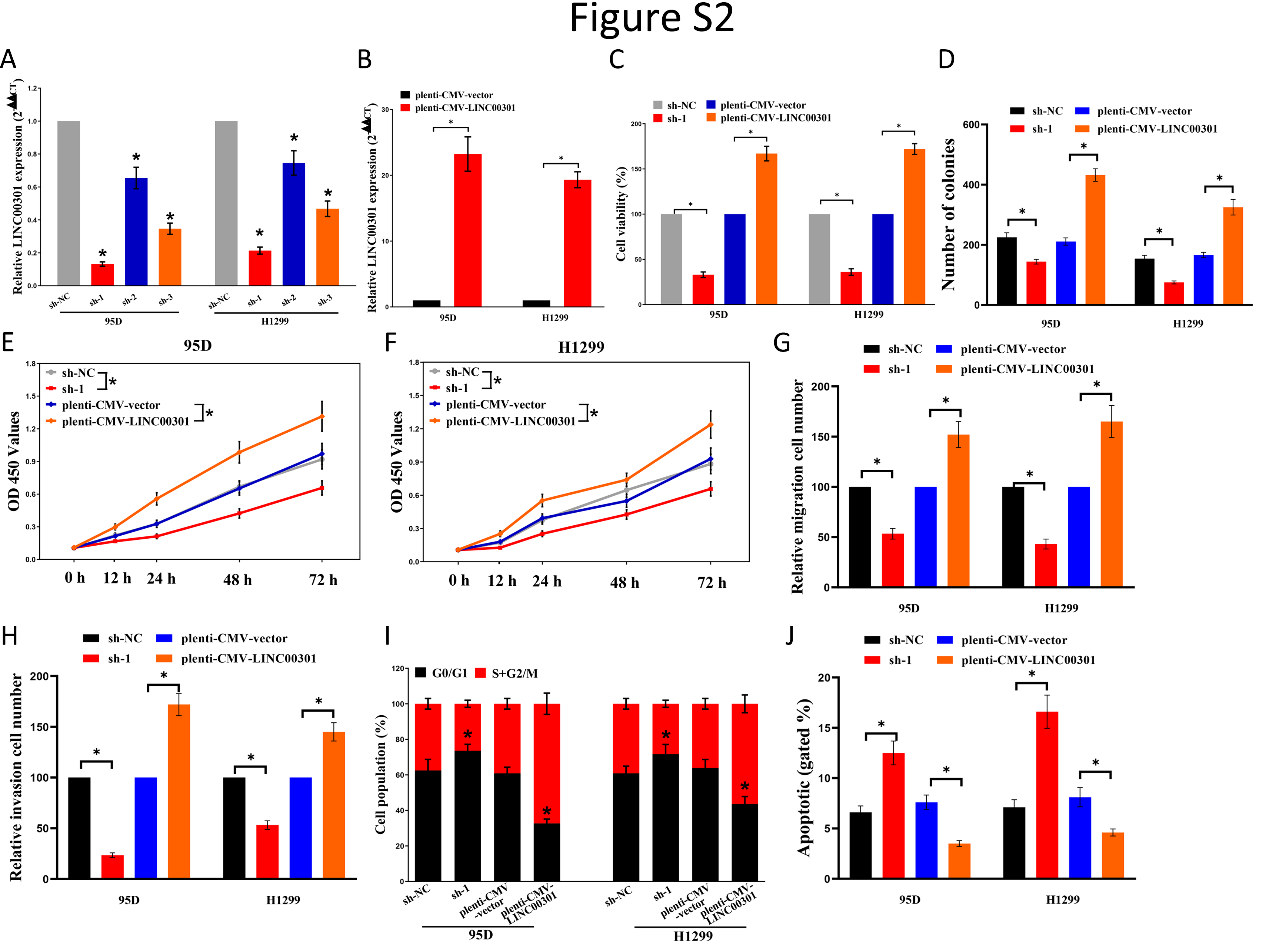


**Fig S2.** LINC00301’s effect on NSCLC cell proliferation, migration and invasion, cell cycle, and cell apoptosis *in vitro*. **A-B**. The efficiency of LINC00301 KD and OE vector transfection. **C**. Trypan blue staining is used to test LINC00301 on NSCLC cell vitality. **D.** Colony formation assay indicates LINC00301 on NSCLC cell proliferation. **E-F.** CCK8 assay indicates LINC00301 on NSCLC cell proliferation. **G-H.** Statistics of transwell migration/invasion assay for LINC00301’s role in NSCLC cell migration and invasion ability. **I-J.** Statistics of flow cytometry analysis of 95D and H1299 cells after transfection. Cell cycle analysis revealed that LINC00301 has influenced the proliferation of 95D and H1299 cells by regulating its cell cycle **(I)**, and cell apoptosis analysis revealed that LINC00301 has influenced the cell apoptosis of 95D and H1299 cells **(K)**. ***p < 0.05, Means ± SD was shown. Statistical analysis was conducted using student t-test analysis.


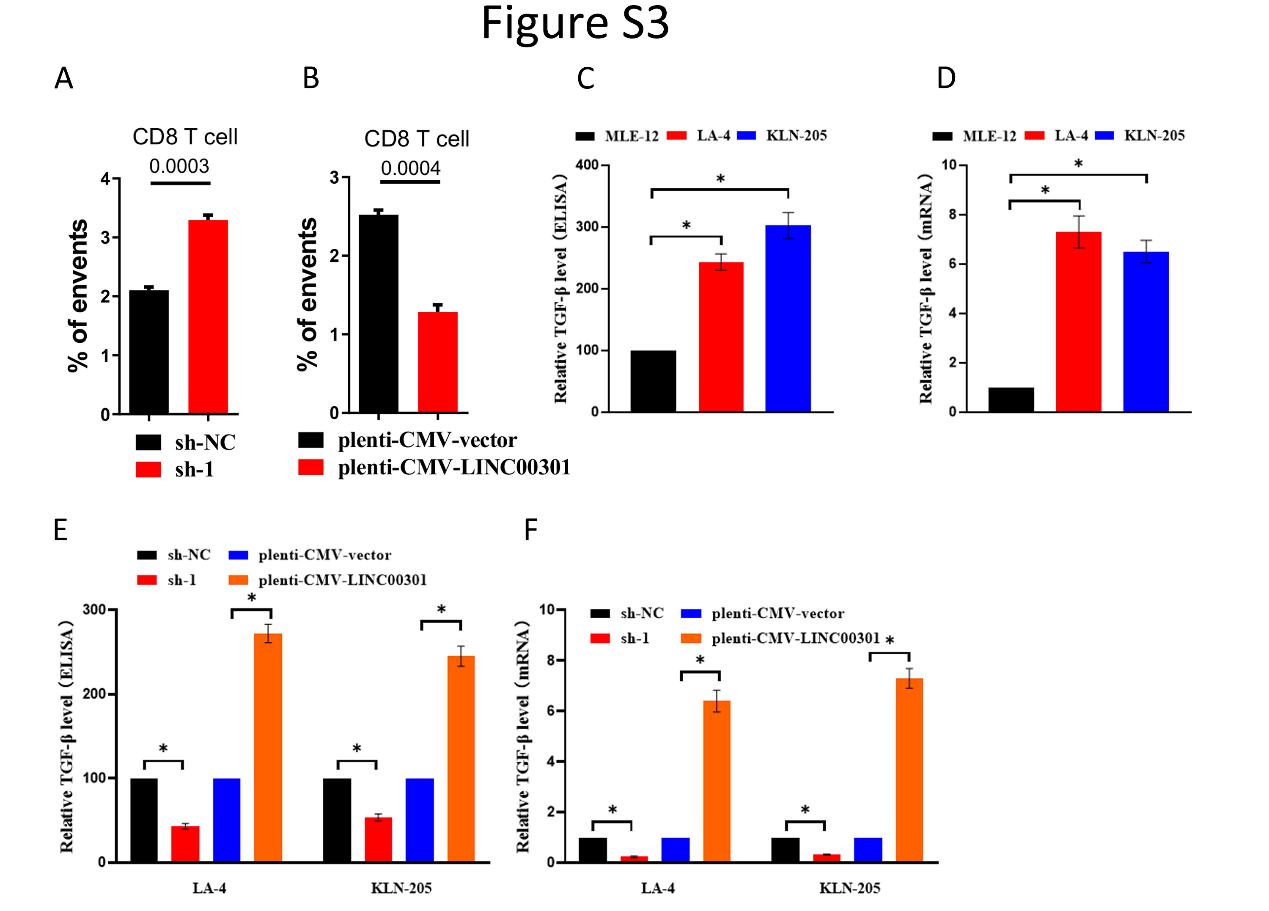


**Fig S3.** LINC00301 facilitates regulatory T cells infiltration in tumors isolated from NSCLC cell lines planted C57BL/6J mice through targeting TGF-β. **A-B**. Representing images for cyTOF analysis of tumors by PhenoGraph analysis, identifying 22 clusters, colored by cluster identification number, and plotted according to tSNE1, tSNE2, identified multiple clusters with apparent changes between groups. **C.** ELISA for TGF-β in the supernatant of MLE-12, LA-4, and KLN-205 cells. **D.** qPCR for TGF-β in the MLE-12, LA-4, and KLN-205 cells. **E.** ELISA for TGF-β in the supernatant of LA-4, and KLN-205 cells treated with LINC00301 KD or OE. **F.** qPCR for TGF-β in the MLE-15, LA-4, and KLN-205 cells treated with LINC00301 KD or OE. Assays were performed in triplicate. ***p < 0.05, Means ± SD are shown. Statistical analysis was conducted using the student t-test.

**
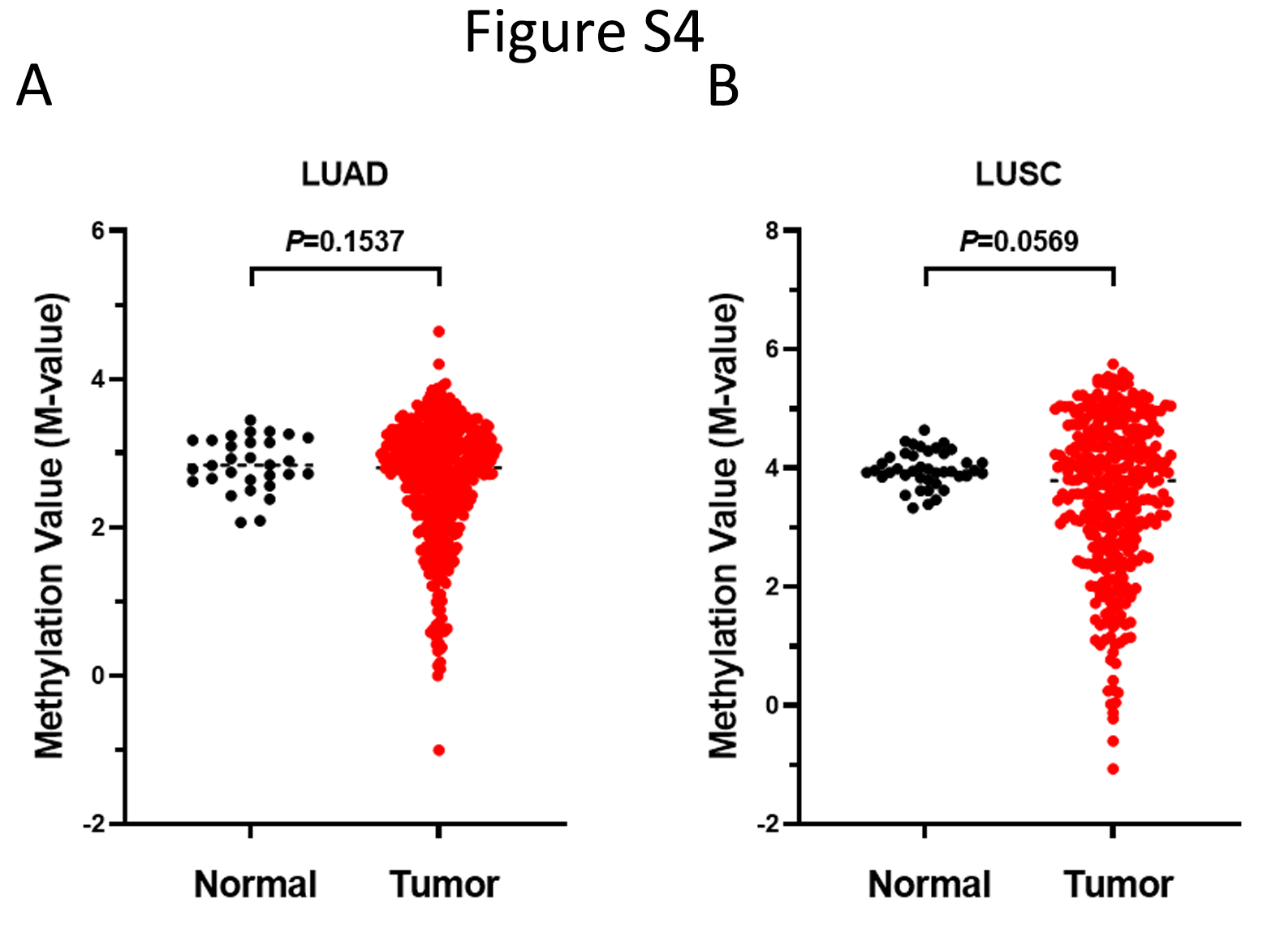
**

**Fig S4.** Correlation of DNA methylation of LINC00301 in LUAD vs normal and LUSC vs normal was conducted using the SMART App (<http://www.bioinfo-zs.com/smartapp/>) that based on TCGA Pan-Cancer cohort of UCSC Xena public data hubs (https://xenabrowser.net). Results indicated that DNA methylation of LINC00301 showed no significant difference between tumor and normal group both in LUAD (normal (n =30); tumor (n =458); p =0.1537) **(A)**and LUSC (normal (n =41); tumor (n= 364); p =0.0569) **(B)**. Means ± SD was shown. Statistical analysis was performed by student’s t-test analysis.


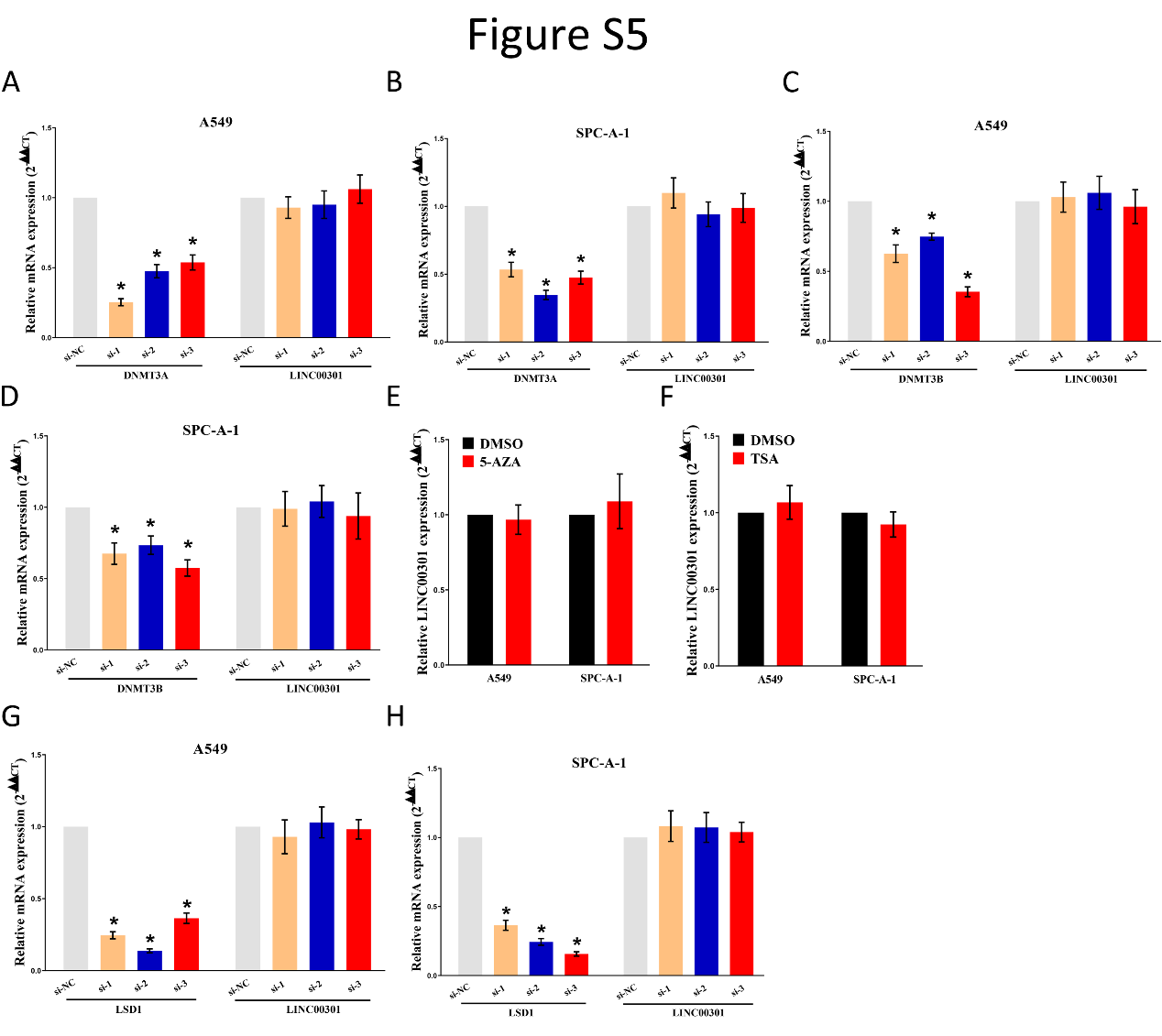


**Fig S5.** Transcription factor FOXC1, not methylation and deacetylation regulated the expression of LINC00301. **A-B.** The role of silence DNMT3A on the expression of LINC00301 in A549 and SPC-A-1 cells. **C-D.** The role of silence DNMT3B on the expression of LINC00301 in A549 and SPC-A-1 cells. **E.** The role of 5-AZA (5-Azacytidine 5 uM) on LINC00301 expression in A549 and SPC-A-1 cells. **F.** The role of TSA (trichostatin A 300 nM) on LINC00301 expression in A549 and SPC-A-1 cells. **G-H.** The role of silence LSD1 on the expression of LINC00301 in A549 and SPC-A-1 cells. Assays were performed in triplicate. **P <* 0.05, Means ± SD are shown. Statistical analysis was conducted using the student t-test.

**
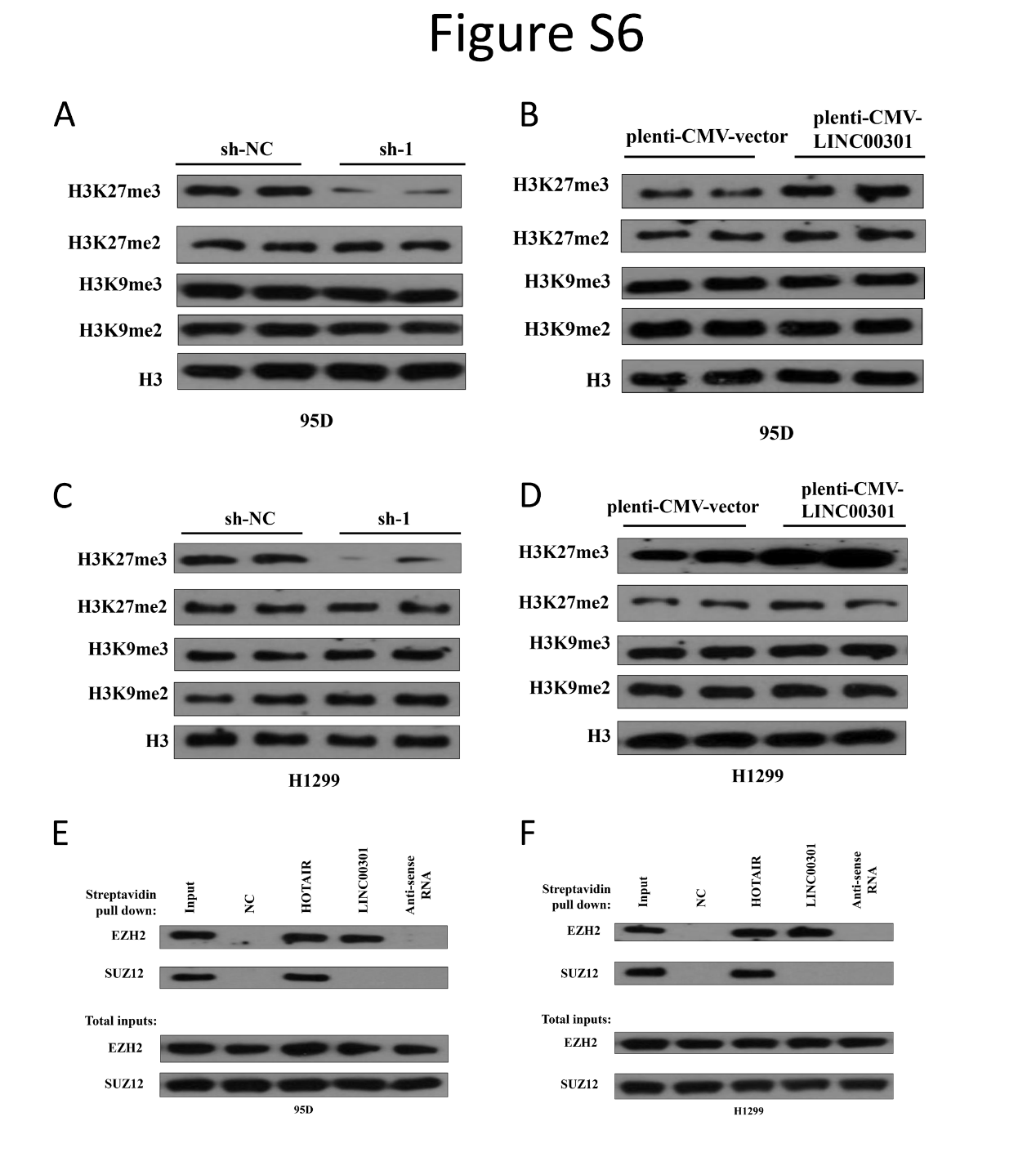
Fig S6.** LINC00301 positively regulates H3K27 trimethylation via its interaction with the catalytic subunit of PRC2. **A-D.** Representative western blot analyses of H3 methylation, as indicated, in95D and H1299 cells in which LINC00301 was knocked down or overexpressed. Methylated H3 levels were normalized to total H3 levels. **E-F.** Representative western blot analysis of a tagged-RNA streptavidin pulldown assay. Biotinylated full-length (WT) LINC00301 was incubated with 95D and H1299 cell lysates in RNA-protein binding buffer, followed by streptavidin-bead pulldown. PRC2 components including SUZ12 and EZH2 were detected by immunoblotting in the pulldown products (top set) and total inputs (bottom set). Assays were performed in triplicate.


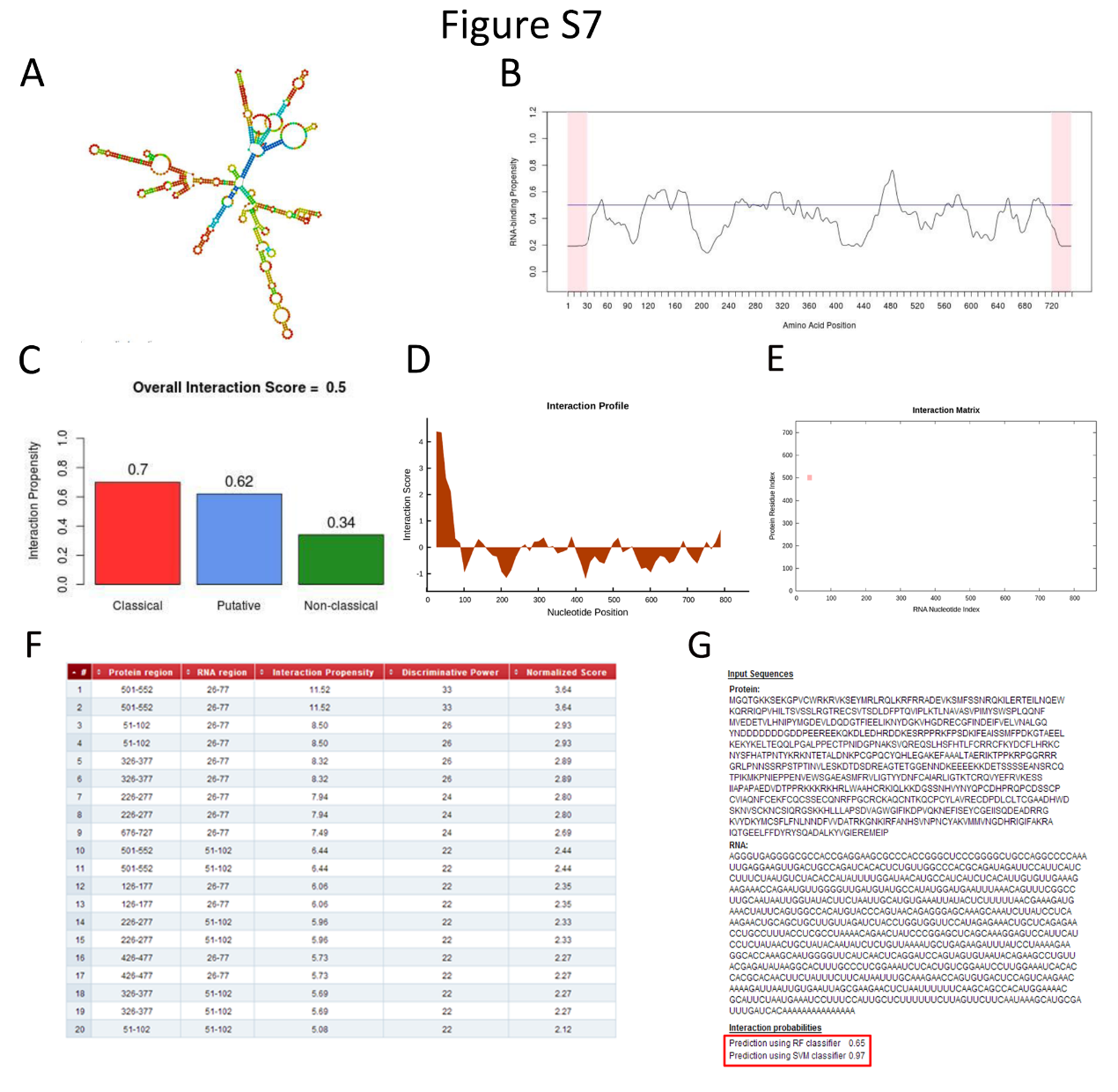


**Fig S7**. Bioinformatics predicts the binding potential of LINC00301 to EZH2 protein. **A**. RNAfold web server predicted the RNA structure of LINC00301. **B-F**. catRAPID predicted the binding potential of LINC00301 to EZH2 protein. **G**. RPISeq predicted the binding potential of LINC00301 to EZH2 protein.


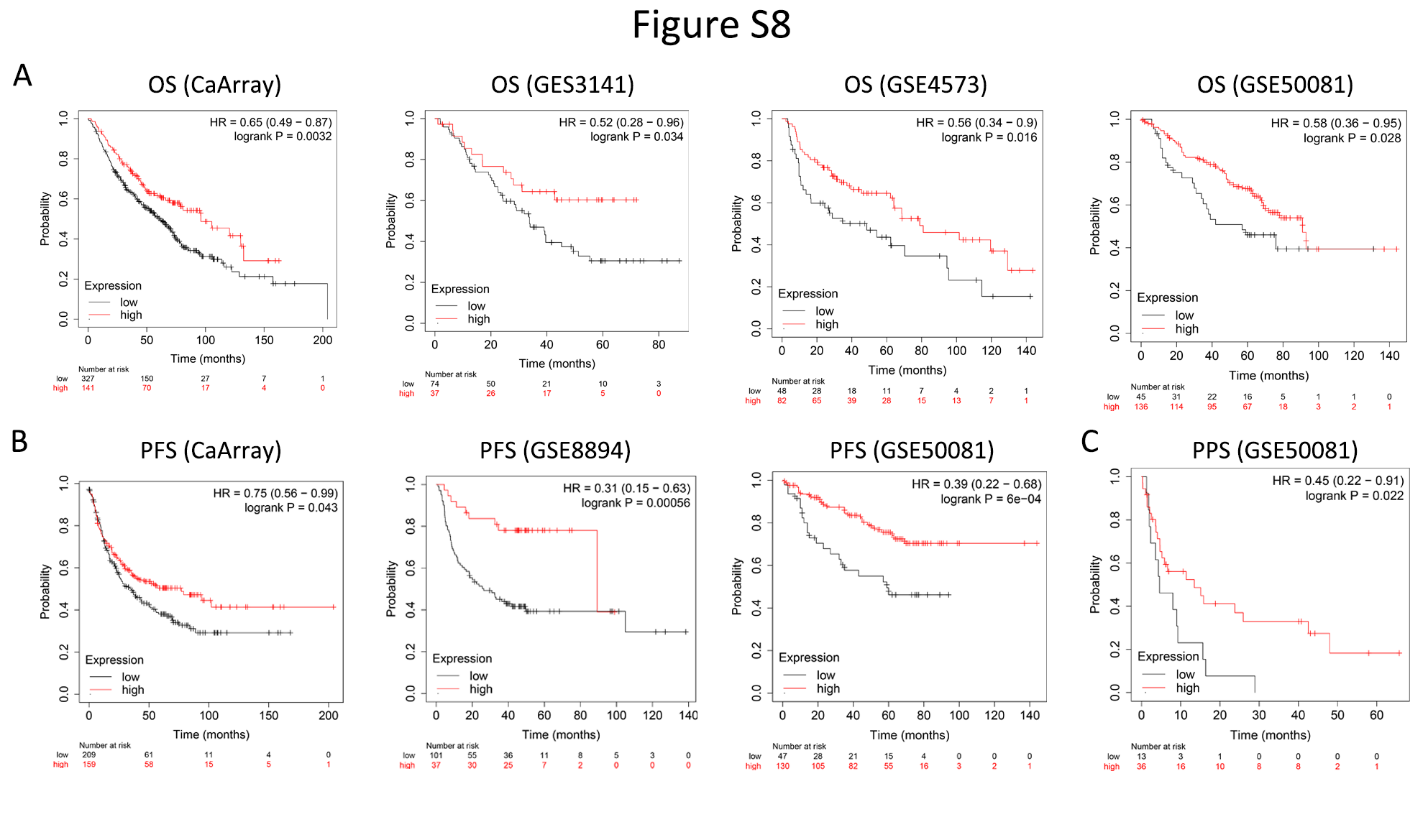


**Fig S8**. Prognostic significance of EAF2 in lung cancer. **A-C**. The effect of EAF2 mRNA expression level on the overall survival (OS), progression-free survival (PFS), and post-progression survival (PPS) in lung cancer patients was analyzed and the Kaplan-Meier plots were generated by Kaplan-Meier Plotter (http://www.kmplot.com).


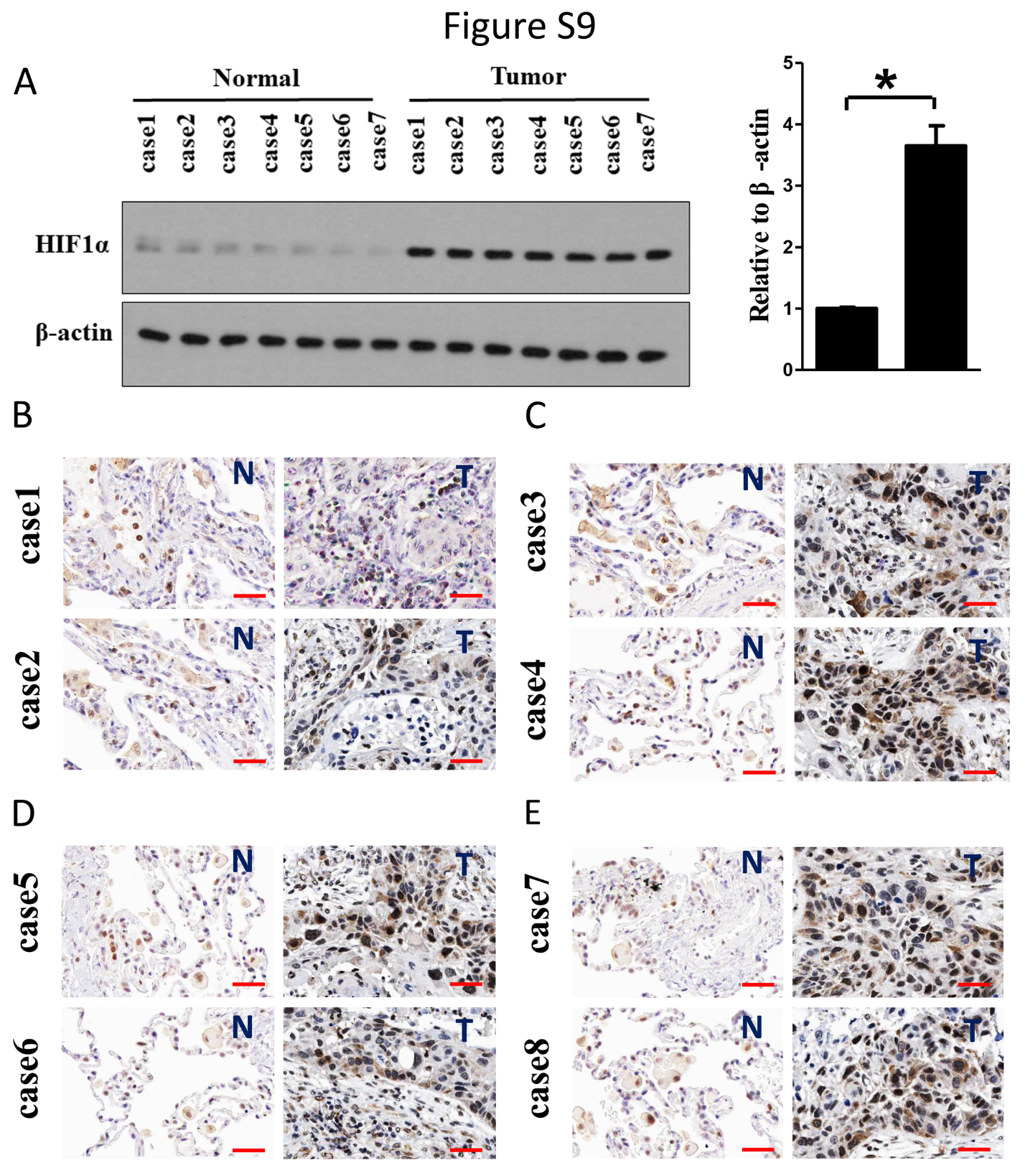


**Fig S9.** HIF1α is higher expressed in NSCLC tumor tissues than adjacent normal lung tissues. **A**. IB tests HIF1α expression in NSCLC tumor tissues and adjacent normal lung tissues. **B-E**. IHC examines HIF1α expression in NSCLC tumor tissues and adjacent normal lung tissues. Assays were performed in triplicate. * p < 0.05, Means ± SD was shown. Statistical analysis was conducted using the student t-test.


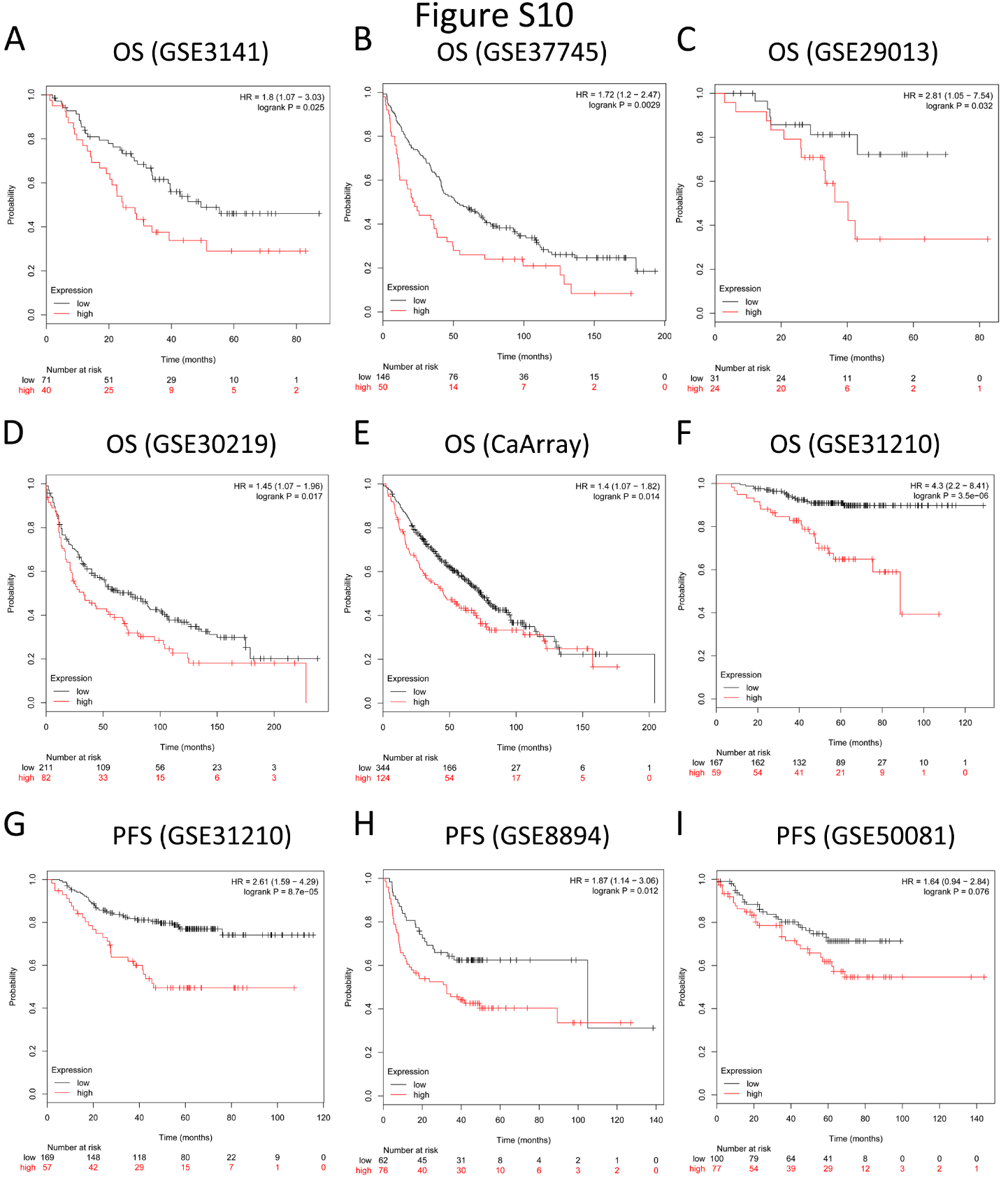


**Fig S10.** Prognostic significance of HIF1α in lung cancer. **A-C**. The effect of HIF1α mRNA expression level on the OS, PFS, and PPS in lung cancer patients were analyzed and the Kaplan-Meier plots were generated by Kaplan-Meier Plotter (http://www.kmplot.com). **D-F**. The effect of HIF1α mRNA expression level on the OS, PFS, and PPS in lung adenocarcinoma patients were analyzed and the Kaplan-Meier plots were generated by Kaplan-Meier Plotter (http://www.kmplot.com). **G-I**. The effect of HIF1α mRNA expression level on the OS, PFS, and PPS in lung squamous cell carcinoma patients were analyzed and the Kaplan-Meier plots were generated by Kaplan-Meier Plotter (<http://www.kmplot.com>).


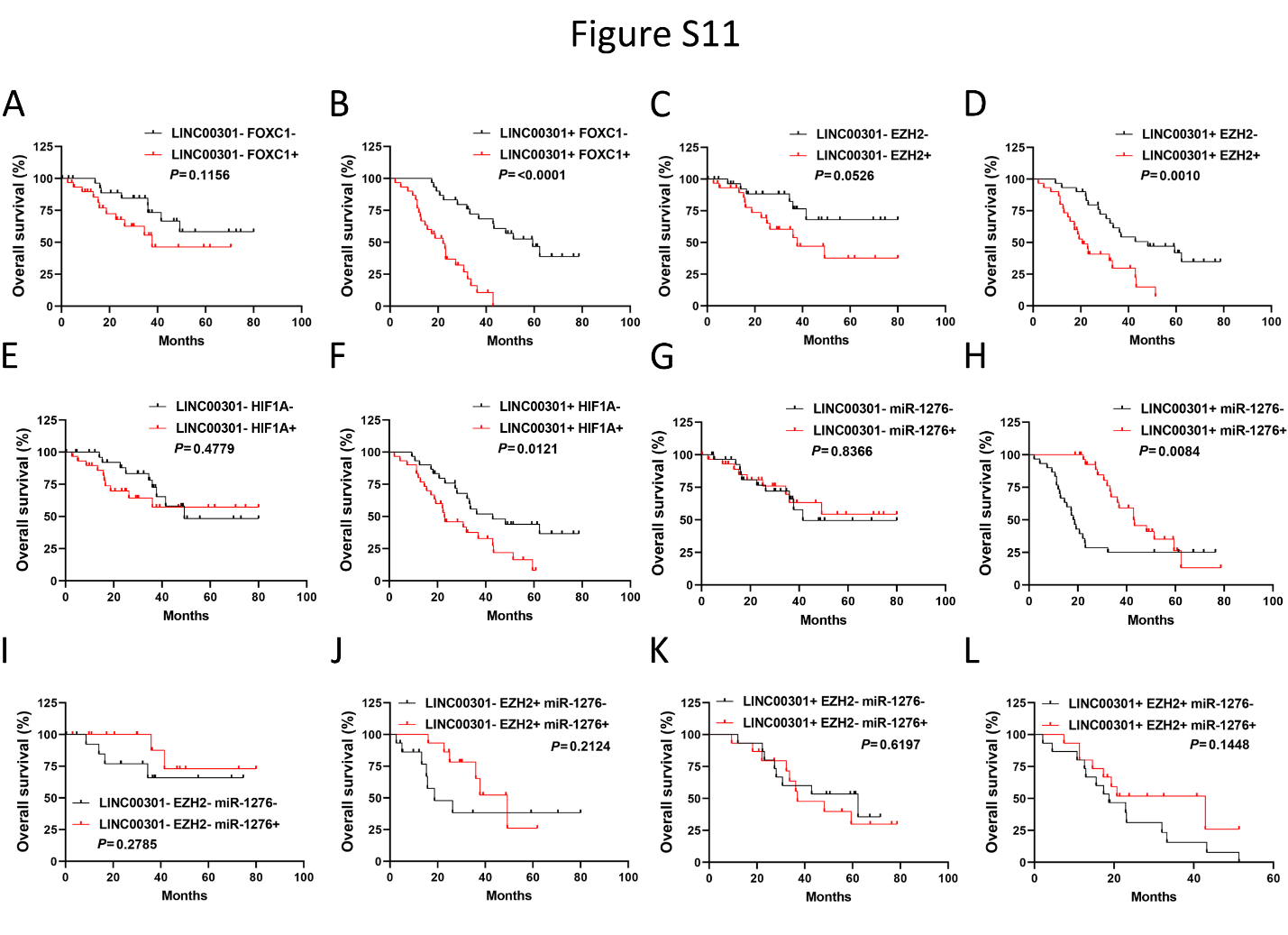


**Fig S11.** Prognostic significance of FOXC1/LINC00301/EZH2/HIF1α, and LINC00301/miR-1276/HIF1A in lung cancer. **A**. The effect of FOXC1 expression level on the overall survival in LINC00301 lower expressed lung cancer patients. **B**. The effect of FOXC1 expression level on the overall survival in LINC00301 higher expressed lung cancer patients. **C**. The effect of EZH2 expression level on the overall survival in LINC00301 lower expressed lung cancer patients. **D**. The effect of EZH2 expression level on the overall survival in LINC00301 higher expressed lung cancer patients. **E**. The effect of HIF1A expression level on the overall survival in LINC00301 lower expressed lung cancer patients. **F**. The effect of HIF1A expression level on the overall survival in LINC00301 higher expressed lung cancer patients. **G**. The effect of miR-1276 expression level on the overall survival in LINC00301 lower expressed lung cancer patients. **H**. The effect of miR-1276 expression level on the overall survival in LINC00301 higher expressed lung cancer patients. **I**. The effect of miR-1276 expression level on the overall survival in LINC00301 lower and EZH2 lower expressed lung cancer patients. **J**. The effect of miR-1276 expression level on the overall survival in LINC00301 lower and EZH2 higher expressed lung cancer patients. **K**. The effect of miR-1276 expression level on the overall survival in LINC00301 higher and EZH2 lower expressed lung cancer patients. **L**. The effect of miR-1276 expression level on the overall survival in LINC00301 higher and EZH2 higher expressed lung cancer patients.


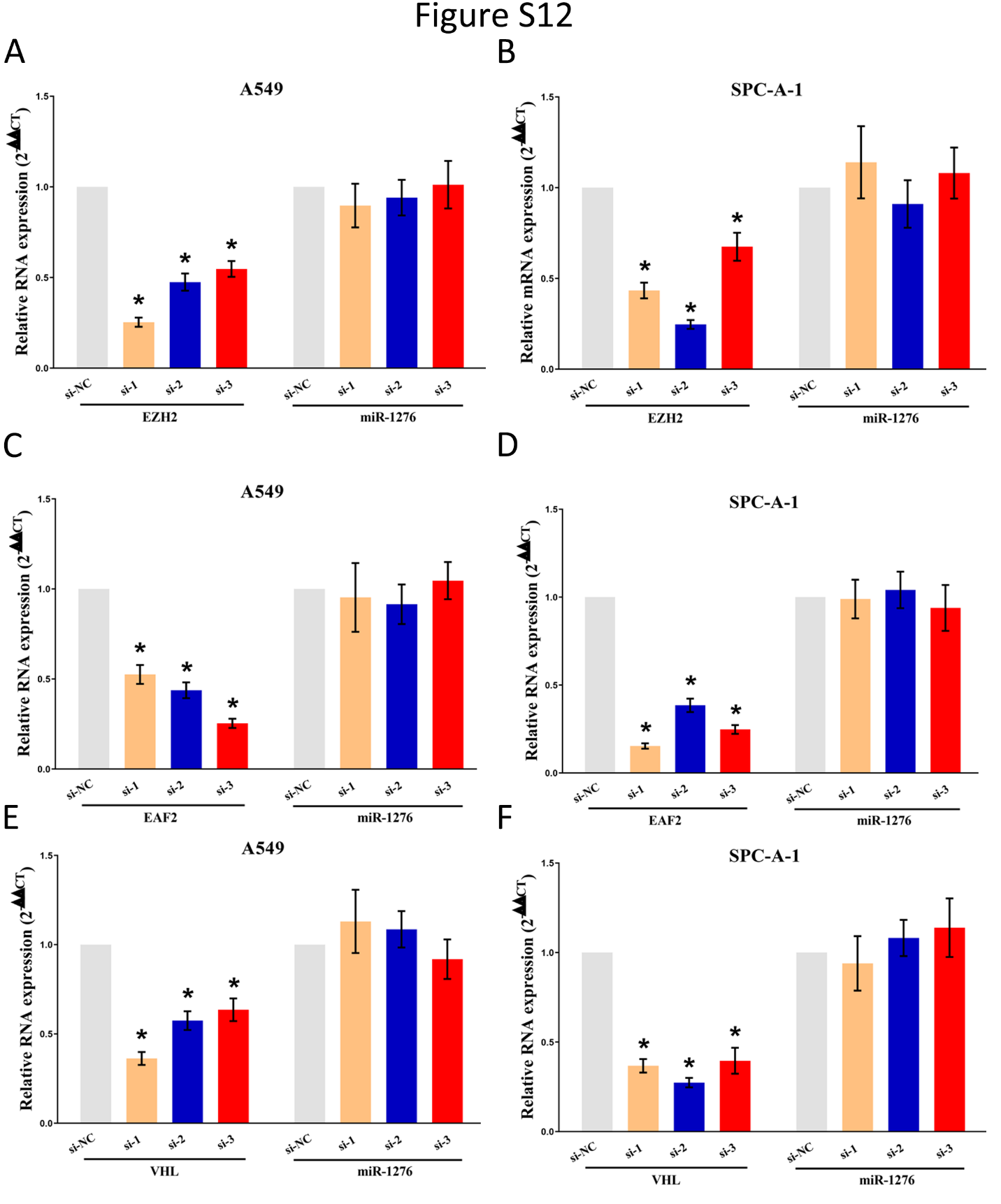


**Fig S12.** Roles of EZH2, EAF2, VHL on miR-1276 expression. **A-B.** Silencing EZH2 in A549 and SPC-A-1 cells, and then tested miR-1276 expression. **C-D.** Silencing EAF2 in A549 and SPC-A-1 cells, and then tested miR-1276 expression. **E-F.** Silencing VHL in A549 and SPC-A-1 cells, and then tested miR-1276 expression. Assays were performed in triplicate. ***p *<* 0.05, Means ± SD are shown. Statistical analysis was conducted using the student t-test.

**
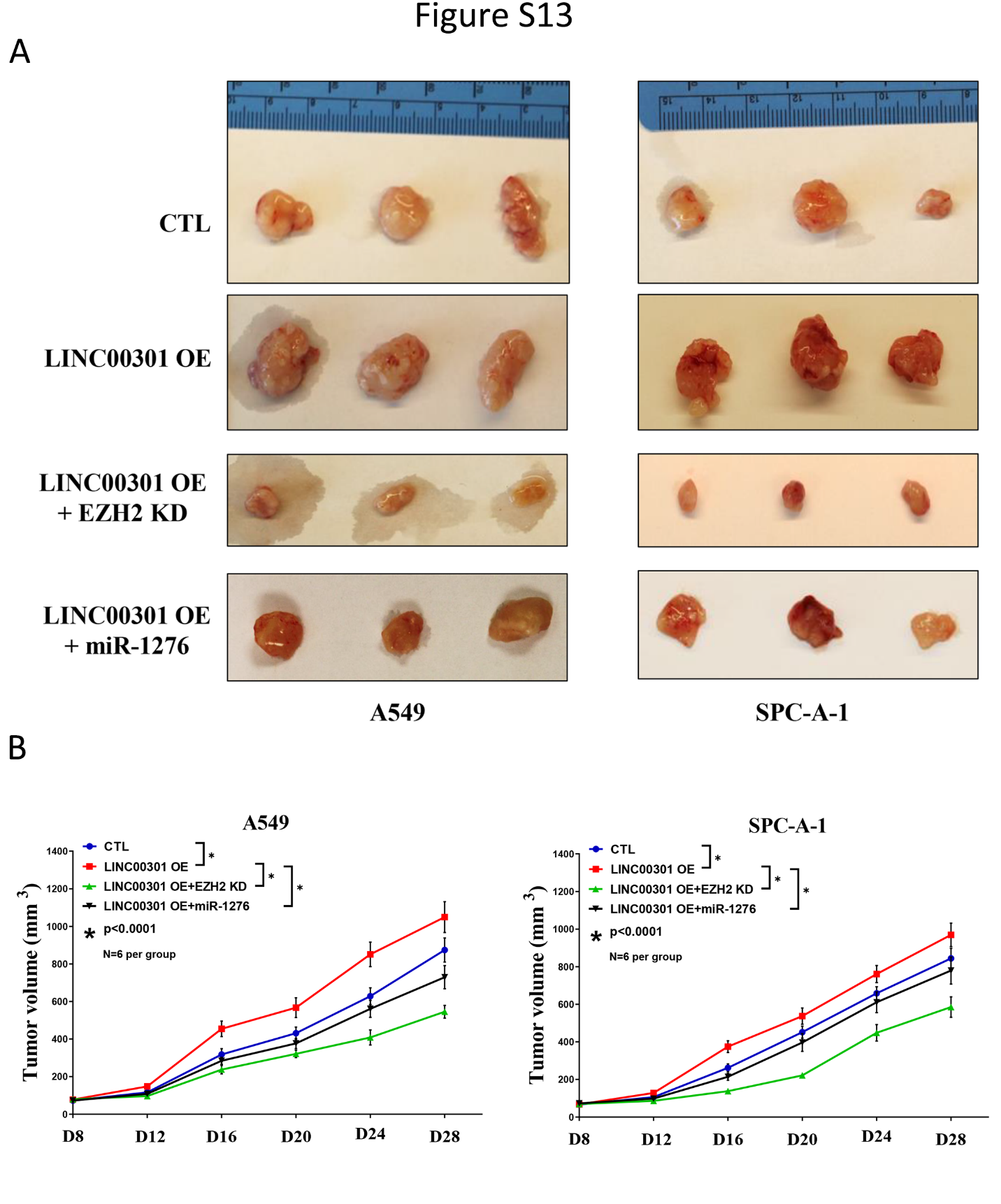
**

**Fig S13.** The roles of LINC00301/EZH2, and LINC00301/miR-1276 axis on tumor growth *in vivo*. **A**. Representative images tumors isolated from nude mice. **B**. Tumor volume in nude mice. Each group contained six mice (n = 6).
